# Bis‐Cyclometalated Indazole Chiral‐at‐Rhodium Catalyst for Asymmetric Photoredox Cyanoalkylations

**DOI:** 10.1002/chem.201903369

**Published:** 2019-11-13

**Authors:** Philipp S. Steinlandt, Wei Zuo, Klaus Harms, Eric Meggers

**Affiliations:** ^1^ Fachbereich Chemie Philipps-Universität Marburg Hans-Meerwein-Strasse 4 35043 Marburg Germany

**Keywords:** asymmetric catalysis, bis-cyclometalated, chiral-at-metal, metal-centered-chirality, photoredox catalysis, rhodium

## Abstract

A new class of bis‐cyclometalated rhodium(III) catalysts containing two inert cyclometalated 6‐*tert*‐butyl‐2‐phenyl‐2*H*‐indazole ligands and two labile acetonitriles is introduced. Single enantiomers (>99 % *ee*) were obtained through a chiral‐auxiliary‐mediated approach using a monofluorinated salicyloxazoline. The new chiral‐at‐metal complex is capable of catalyzing the visible‐light‐induced enantioselective α‐cyanoalkylation of 2‐acyl imidazoles in which it serves a dual function as the chiral Lewis acid catalyst for the asymmetric radical chemistry and at the same time as the photoredox catalyst for the visible‐light‐induced redox chemistry (up to 80 % yield, 4:1 d.r., and 95 % *ee*, 12 examples).

## Graphical abstract

**A new chiral‐at‐metal rhodium(III) complex** composed of two cyclometalated 2‐phenyl‐2*H*‐indazole ligands and two labile acetonitrile ligands permits the photoinduced enantioselective cyanoalkylation of 2‐acyl imidazoles. The new catalyst serves a dual function as a chiral Lewis acid catalyst for the asymmetric radical chemistry and at the same time as the photoredox catalyst for the visible‐light‐induced redox chemistry.

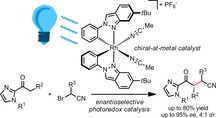

## Introduction

Chiral transition‐metal complexes are a prominent and powerful class of asymmetric catalysts, traditionally assembled from chiral organic ligands and metal salts or organometallic precursor complexes.^1^ The chiral organic ligands are typically involved in the asymmetric induction but also control the relative and absolute configuration of the transition metal complexes. Following a different strategy, we and others have recently demonstrated that chiral transition metal complexes composed from entirely achiral ligands can be exquisite transition‐metal catalysts for a wide variety of asymmetric conversions, including asymmetric photocatalysis.^2^, ^3^, ^4^ Such chiral‐at‐metal complexes rely on a configurationally stable stereogenic metal center^5^ for generating metal‐centered chirality which at the same time must be a reactive metal center for performing the asymmetric catalysis.^6^

**Figure 1:**
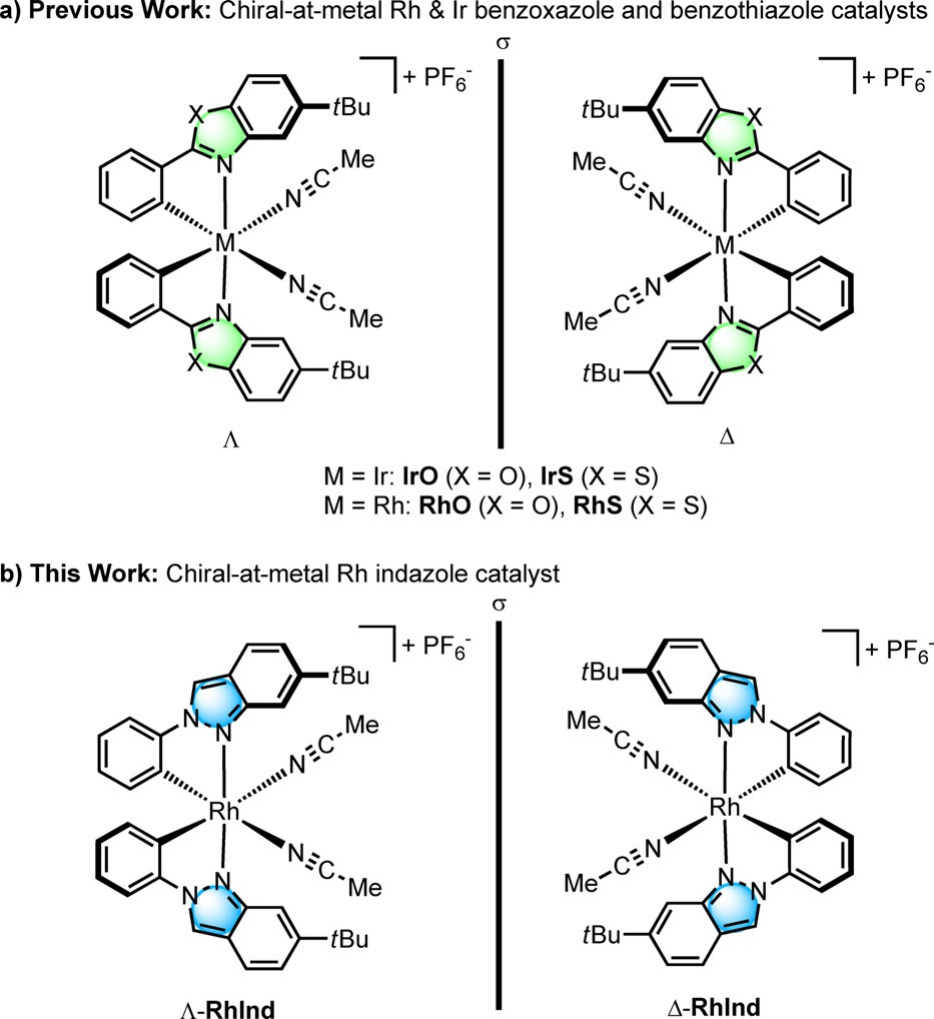
Previous catalyst design and this work regarding bis‐cyclometalated chiral‐at‐metal rhodium and iridium catalysts for asymmetric conversions.

Our initial design was based on bis‐cyclometalated iridium(III) and rhodium(III) complexes, in which two 5‐*tert*‐butyl‐2‐phenylbenzoxazoles (**IrO** 7 and **RhO** 8) or 5‐*tert*‐butyl‐2‐phenylbenzothiazoles (**IrS** 9 and **RhS** 10) implement a stereogenic metal center with either a left‐handed (Λ‐configuration) or right‐handed (Δ‐configuration) overall helical topology (Figure 1). These two cyclometalated ligands are configurationally inert so that the overall stereochemical information is retained in these complexes once they are generated in a non‐racemic fashion. Two additional monodentate acetonitrile ligands are labile and provide access of substrates or reagents to the metal center.

We found that the nature of the cyclometalating ligand has a profound influence on the reactivity and stereoselectivity of the bis‐cyclometalated iridium(III) and rhodium(III) complexes^2^ and we were therefore seeking to investigate ligands that differ from our previous benzoxazole and benzothiazole systems. Here we now introduce a new class of bis‐cyclometalated chiral‐at‐metal rhodium(III) catalysts which are based on two cyclometalated 6‐*tert*‐butyl‐2‐phenyl‐2*H*‐indazole ligands (Λ‐ and Δ‐**RhInd**). We demonstrate that this **RhInd** catalyst is superior for the visible‐light‐induced enantioselective α‐cyanoalkylation of 2‐acyl imidazoles in which **RhInd** serves a dual function as the chiral catalyst but is also involved in the photochemical induction.

## Results and Discussion

### Design and synthesis of the rhodium catalyst

**Scheme 1:**
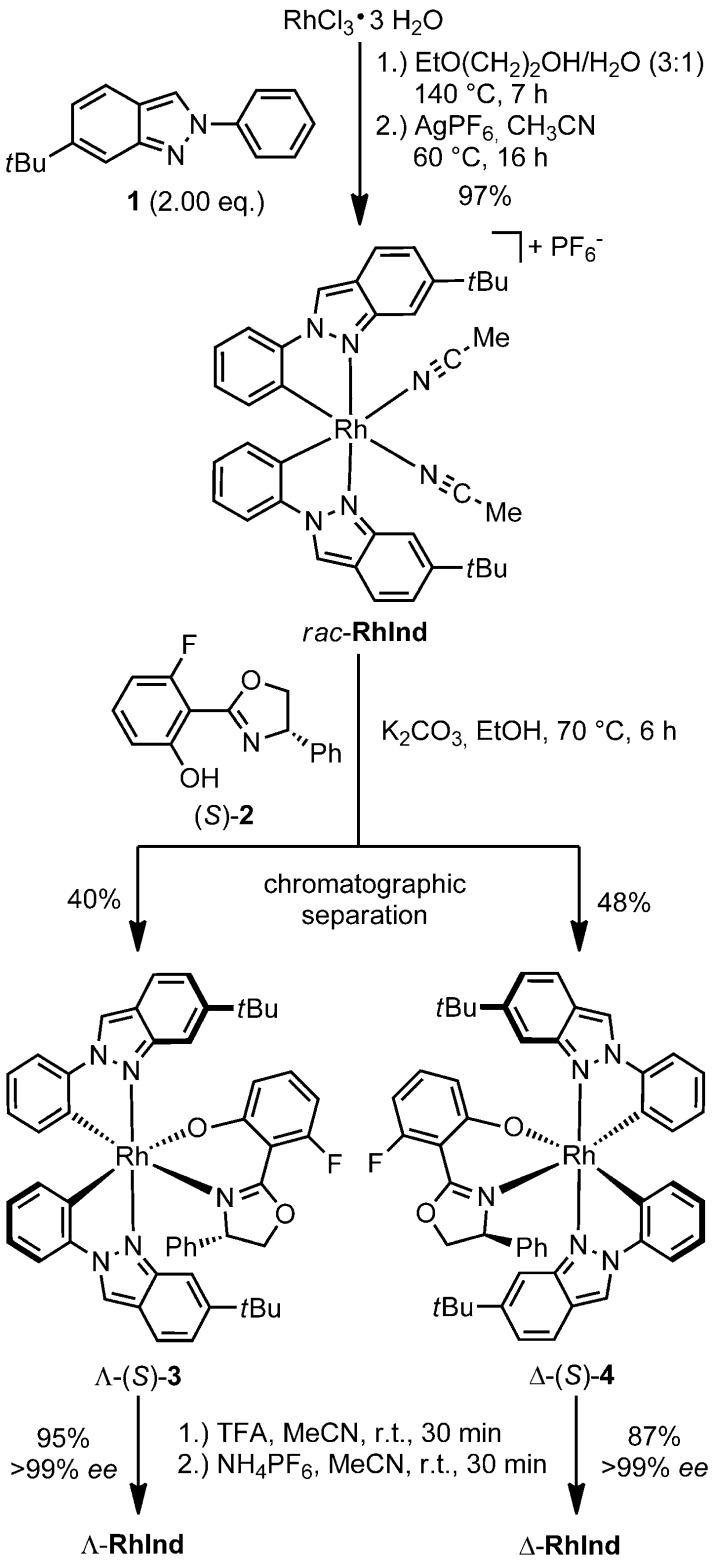
Auxiliary‐mediated synthesis of enantiopure Λ‐ and Δ‐**RhInd**.

As part of our ongoing interest in expanding the structural diversity of bis‐cyclometalated rhodium‐complexes, we chose 2‐phenyl‐2*H*‐indazole as an interesting candidate. Bis‐cyclometalated iridium complexes with 2‐phenyl‐2*H*‐indazoles are well established^11^ but the analogous rhodium(III) complexes have not been reported. The overall geometry of this ligand is comparable to our previously applied benzoxazole and benzothiazole ligands, however, the electron‐rich aromatic system of 2*H*‐indazoles provides significantly distinct electronics that might enable new catalytic transformations. The chiral‐auxiliary‐mediated synthesis^12^, ^13^, ^14^ of the enantiopure catalyst **RhInd** started with the reaction of rhodium trichloride hydrate with 2.0 equivalents of 6‐*tert*‐butyl‐2‐phenyl‐2*H*‐indazole (**1**), followed by addition of 2.0 equivalents of AgPF_6_ in MeCN to obtain bis‐cyclometalated *rac*‐**RhInd** in 97 % yield (Scheme 1). Afterwards, the racemic product was reacted with the monofluorinated salicyloxazoline (*S*)‐**2** 10, 15, 16 to provide the two diastereomers Λ‐(*S*)‐**3** and Δ‐(*S*)‐**4** in 40 % and 48 % yield, respectively, which were separated by column chromatography on deactivated silica gel. The required high diastereomeric purity of the isolated auxiliary complexes was evaluated by ^1^H NMR and ^19^F NMR spectroscopy. Cleavage of the auxiliary ligand was subsequently performed under acidic conditions using trifluoroacetic acid (TFA), followed by anion exchange with NH_4_PF_6_ to provide the individual enantiomers Λ‐**RhInd** (95 % yield) and Δ‐**RhInd** (87 % yield).

**Figure 2:**
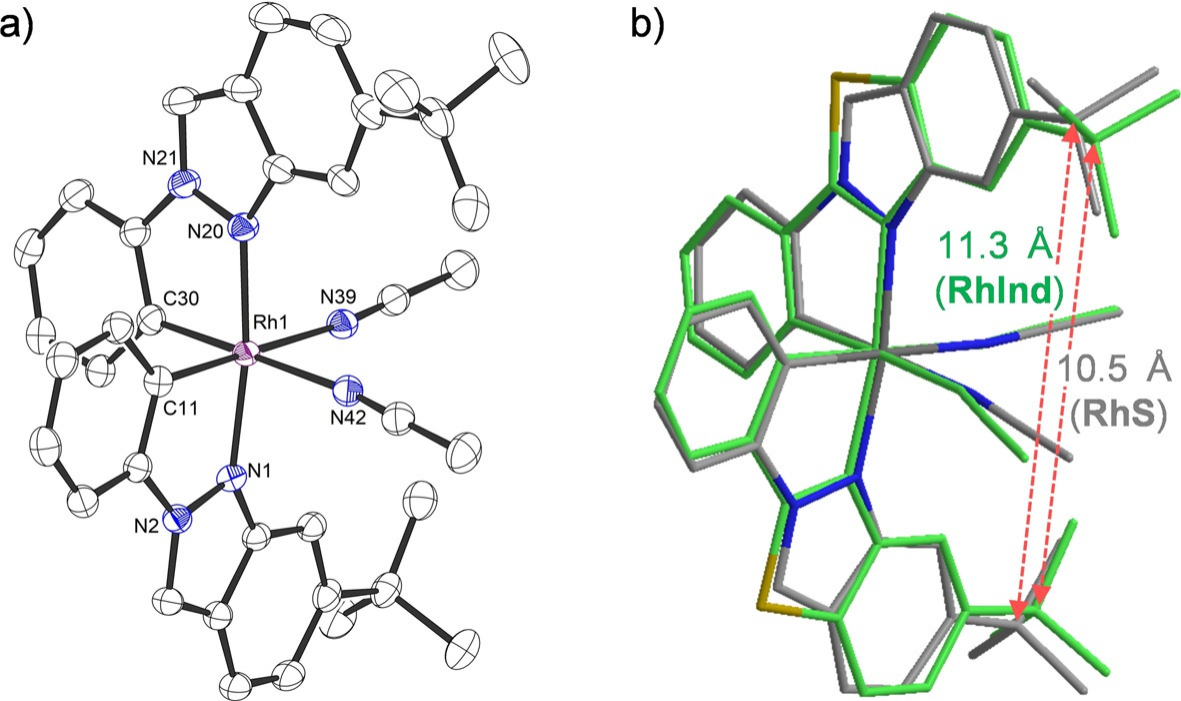
a) Crystal structure of Λ‐**RhInd**. ORTEP drawing with 50 % probability thermal ellipsoids. Hexafluorophospate counterion and solvent molecules are omitted for clarity. b) Superimposed crystal structure of Λ‐**RhInd** (grey) with Λ‐**RhS** (green). Fitted are the central metals together with the metal‐coordinated atoms.

**Figure 3:**
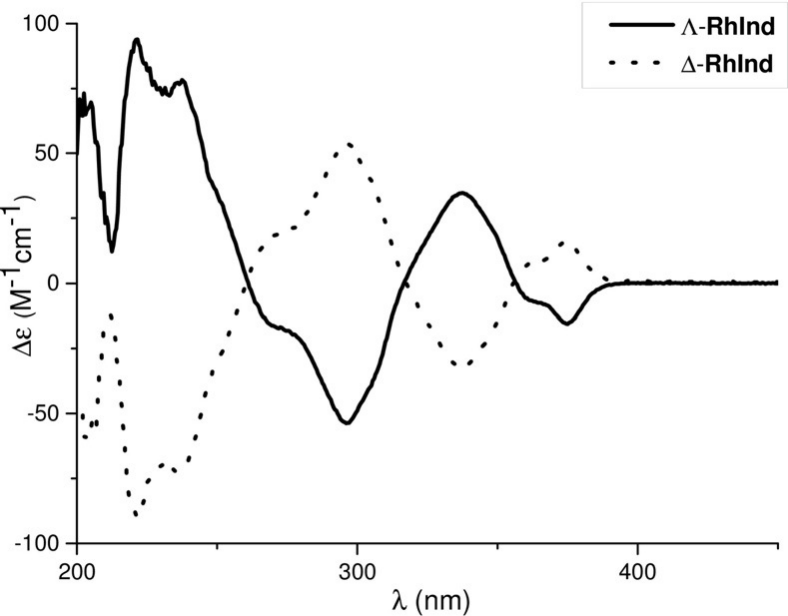
CD spectra of Λ‐ and Δ‐**RhInd** measured in MeOH (0.20 mm).

**Figure 4:**
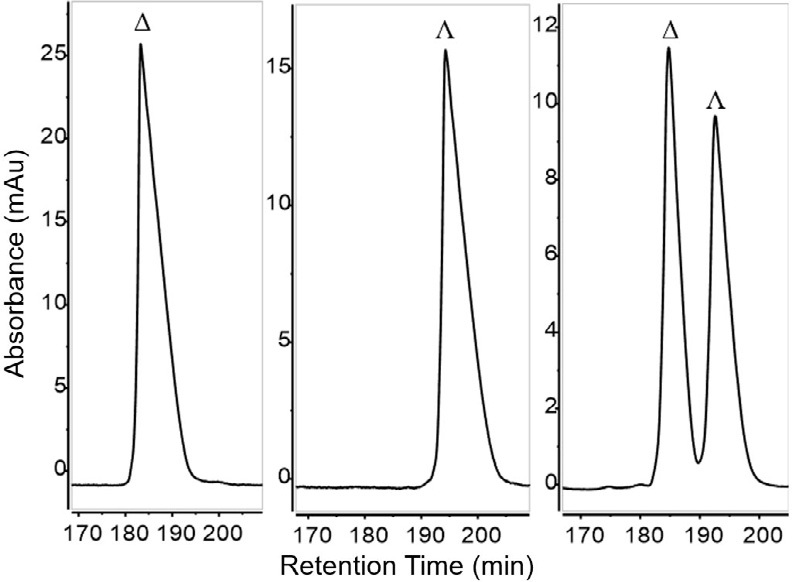
HPLC traces of Δ, Λ, and racemic **RhInd**. HPLC conditions: Daicel CHIRALPAK IB‐N5, 250×4.6 mm, column temp.=25 °C, λ_abs_=254 nm, flow rate=0.6 mL min^−1,^ solvent A=0.1 % aqueous TFA, solvent B=MeCN, gradient=40 % to 50 % B in 180 min.

The absolute configuration was assigned based on a crystal structure of Λ‐**RhInd** (Figure 2 a). The CD spectra of Λ‐ and Δ‐**RhInd** are shown in Figure 3 and confirm their mirror‐imaged structures. HPLC analysis on a chiral stationary phase exhibited an *ee* of >99 % for both the Λ‐ and the Δ‐**RhInd** complex (Figure 4). Superimposition of the crystal structures of **RhInd** and **RhS** reveals a slightly larger distance between the two quaternary carbon atoms of the *tert*‐butyl groups for **RhInd** (11.3 Å) making the catalytic site slightly larger compared to **RhS** (10.5 Å) (Figure 2 b).10a

### Initial experiments and optimization

With the new enantiopure complexes in hand, we next investigated the application of **RhInd** in asymmetric photoredox catalysis.^17^, ^18^ After some initial reaction screening, we were delighted to find that Λ‐**RhInd** (2.0 mol %) catalyzes the α‐cyanomethylation of 2‐acyl imidazole **5 a** with bromoacetonitrile (**6 a**) in the presence of Na_2_HPO_4_ and under irradiation with blue LEDs to provide (*R*)‐**7 a** with a high *ee* value of 94 % but in only 22 % yield (Table 1, entry 1).^19^ Table 1 shows the stepwise optimization of this enantioselective, visible‐light‐induced cyanoalkylation. First, different solvents were investigated (entries 1–6) and it was found that MeOH/THF 4:1 provided the best results. Changing the base from Na_2_HPO_4_ to 2,6‐lutidine or Cs_2_CO_3_ provided higher yields of 50 % and 73 % but the enantioselectivity dropped to 87 % and 4 % *ee*. (entries 7 and 8). *N*,*N*‐Diisopropylethylamine (DIPEA) as base only provided 2 % yield and 42 % *ee* (entry 9). Despite the low yield, Na_2_HPO_4_ was selected as the most suitable base with respect to enantioselectivity. A higher catalyst loading afforded improved yields but a significantly lower enantioselectivity (entries 10 and 11). The reduced enantioselectivity can be rationalized with a slow **RhInd**‐catalyzed racemization of the product upon coordination to the catalyst, followed by deprotonation and reprotonation (see Supporting Information for more details). Increasing the amount of bromoacetonitrile to 6.0 equivalents improved the yield while maintaining a high *ee* (entry 12). Increasing the amount of base from 1.1 to 1.5 equivalents (entry 13) or 2.0 equivalents (entry 14) provided further improved yields of 78 % or 80 %, respectively. Increasing the amount of base to 2.5 equivalents led to a sharp drop in the yield to 27 %, probably due to the resulting turbidity from the low solubility of Na_2_HPO_4_ having a negative effect on the penetration by the light into the reaction suspension (entry 15). Finally, it is worth noting that we found that small amounts of water provide a beneficial effect, probably by facilitating rapid proton transfer, and therefore stoichiometric amounts of water were added to each reaction shown in Table 1.

**Table 1:** Initial experiments and optimization.^[a]^

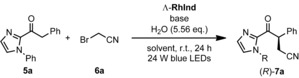						
Entry	Solvent	Base (equiv)	Catalyst (mol %)	Equiv of **6 a**	Yield [%]	*ee* [%]^[b]^
1	MeOH/THF 4:1	Na_2_HPO_4_ (1.1)	2	3.0	22	94
2	MeCN	Na_2_HPO_4_ (1.1)	2	3.0	–	–
3	THF	Na_2_HPO_4_ (1.1)	2	3.0	–	–
4	MeOH	Na_2_HPO_4_ (1.1)	2	3.0	15	80
5	MeOH/CH_2_Cl_2_ 1:1	Na_2_HPO_4_ (1.1)	2	3.0	17	79
6	MeOH/MeCN 4:1	Na_2_HPO_4_ (1.1)	2	3.0	–	–
7	MeOH/THF 4:1	2,6‐Lutidine (1.1)	2	3.0	50	87
8	MeOH/THF 4:1	Cs_2_CO_3_ (1.1)	2	3.0	73	4
9	MeOH/THF 4:1	DIPEA (1.1)^[c]^	2	3.0	2	42
10	MeOH/THF 4:1	Na_2_HPO_4_ (1.1)	4	3.0	47	78
11	MeOH/THF 4:1	Na_2_HPO_4_ (1.1)	8	3.0	99	56
12	MeOH/THF 4:1	Na_2_HPO_4_ (1.1)	2	6.0	76	94
13	MeOH/THF 4:1	Na_2_HPO_4_ (1.5)	2	6.0	78	94
14	MeOH/THF 4:1	Na_2_HPO_4_ (2.0)	2	6.0	80	94
15	MeOH/THF 4:1	Na_2_HPO_4_ (2.5)	2	6.0	27	92

[a] Conditions: 2‐Acyl imidazole **5 a** (0.10 mmol), Λ‐**RhInd** (2–8 mol %) and the corresponding base (0.11–0.25 mmol) were dissolved in the indicated solvent (0.5 mL) under inert gas atmosphere and H_2_O (0.56 mmol) was added. The resulting mixture was stirred for 5 min before bromoacetonitrile (0.30–0.60 mmol) was added and the mixture was degassed via freeze‐pump‐thaw for three cycles. The reaction mixture was then stirred for 24 h under inert gas atmosphere at r.t. in front of blue LEDs (24 W, 10 cm distance). [b] Determined by HPLC analysis on a chiral stationary phase. [c] DIPEA=*N*,*N*‐diisopropylethylamine.

To summarize this part, we found reaction conditions for the photoinduced cyanoalkylation reaction **5 a**+**6 a**→(*R*)‐**7 a** in 80 % yield with 94 % *ee* using 2 mol % of the chiral‐at‐rhodium complex Λ‐**RhInd** as the single catalyst.

### Substrate scope

**Scheme 2:**
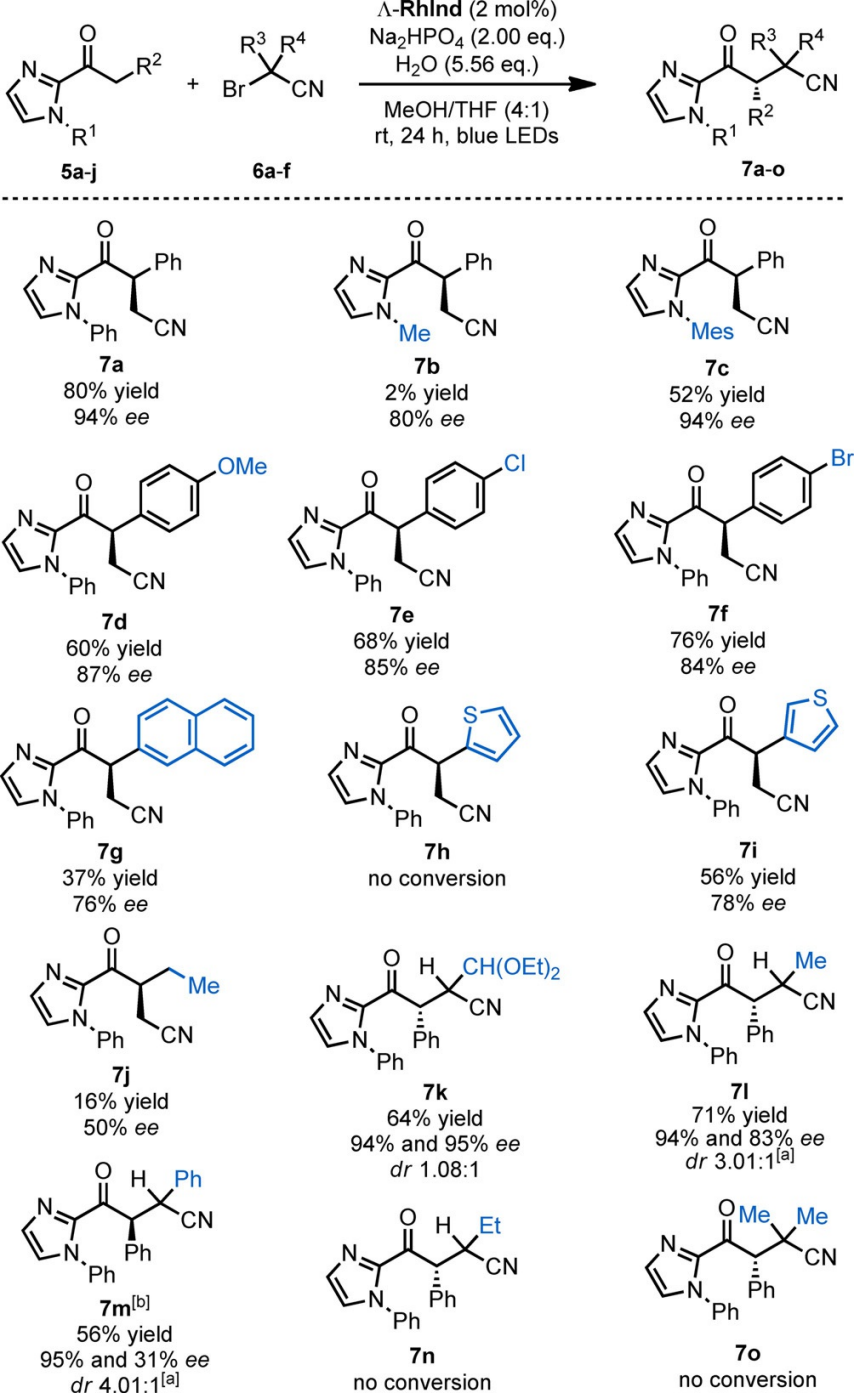
Substrate Scope. [a] Major diastereomer has the higher *ee*. [b] Δ‐**RhInd** was used instead of the Λ‐enantiomer for product **7 m**.

After having established the optimized reaction conditions, we next investigated the scope of the α‐cyanoalkylation with respect to 2‐acyl imidazoles (**5 a**–**j**) and α‐cyano bromides (**6 a**–**f**) (Scheme 2). Substrate **5 a** provided the best results with unbranched bromoacetonitrile (**6 a**) with respect to yield and enantioselectivity (**7 a**). Interestingly, methyl substituted imidazole substrate **5 b** only gave 2 % yield and 80 % *ee* (**7 b**). Mesityl substituted imidazole substrate **5 c** provided 52 % yield and an *ee* of 94 % (**7 c**). The addition of electron withdrawing and electron donating groups at the phenyl moiety had a slightly disadvantageous effect on the yield as well as the enantioselectivity (**7 d**–**f**). The implementation of a naphthyl moiety resulted in a significantly lower yield and a moderate enantioselectivity of 76 % *ee*. (**7 g**). 2‐Thiophenyl substrate **5 h** showed no conversion at all, whereas 3‐thiophenyl substrate **5 i** provided 56 % yield of **7 i** with 78 % *ee*. This can be rationalized by a bidentate coordination of the catalyst to the sulfur atom and the acyl oxygen of substrate **5 h** thus impeding the conversion. Aliphatic substrate **5 j** only provided a low yield of 16 % with 50 % *ee* (**7 j**). Furthermore, five branched α‐cyano bromides were investigated. Diastereoselectivities were validated by ^1^H NMR spectroscopy of the crude products. Product **7 k** was formed in 64 % yield with a d.r. of 1.08:1. Both of the diastereomers showed high *ee* values of 94 % and 95 %. Product **7 l** was formed with a d.r. of 3.01:1 with the major diastereomer exhibiting an *ee* of 94 %. When α‐bromophenylacetonitrile was used, the cyanoalkylation product **7 m** was obtained in 56 % yield with a d.r. of 4.01:1. Interestingly, the major diastereomer showed a very good *ee* of 95 % while the minor diastereomer was obtained with only 31 % *ee*. Unfortunately, butyronitrile and isobutyronitrile did not form any cyanoalkylation products (**7 n** and **7 o**).

### Mechanism of the cyanoalkylation

#### Mechanistic proposal

We previously reported a series of visible‐light‐induced enantioselective α‐alkylations of 2‐acyl imidazoles using electron deficient benzyl bromides, phenacyl bromides, perfluoroalkyl halides, and enantioselective trichloromethylations with BrCCl_3_.^2^, 9a, 20, 21 These photoreactions were catalyzed most effectively with bis‐cyclometalated iridium complexes, whereas related photoinduced enantioselective α‐aminations of 2‐acyl imidazoles were catalyzed by the related bis‐cyclometalated rhodium complexes.^2^, ^22^, ^23^ These enantioselective photoredox reactions serve as the basis for the proposed mechanism of the here introduced rhodium‐catalyzed photoinduced α‐cyanoalkylation of 2‐acyl imidazoles. Accordingly, the catalytic reaction begins with the coordination of the 2‐acyl imidazole substrate (e.g. **5 a**) to the rhodium catalyst in a bidentate fashion upon release of the two labile MeCN ligands of **RhInd**, thereby forming intermediate **I**. A subsequent deprotonation induced by the added base Na_2_HPO_4_ generates the rhodium enolate complex **II** which is a key intermediate of this asymmetric photoreaction. It fulfills a dual function as reactive intermediate in the catalytic cycle and as the in situ assembled visible light activatable photoredox catalyst. Upon absorption of visible light, the rhodium enolate acts as a photoexcited reducing agent and transfers a single electron^24^ to the α‐cyanoalkyl bromide (e.g. **6 a**), which in turn fragments into bromide and the α‐cyanoalkyl radical **V**. This free radical **V** is electron deficient due to the electron withdrawing cyano group in α‐position and therefore rapidly reacts with the electron rich double bond of the rhodium enolate **II** to form the ketyl radical intermediate **III** upon formation of a new C−C bond and a stereogenic carbon, the absolute configuration of which is controlled by the chiral rhodium complex.^25^ The ketyl radical **III** is a strong reducing agent and either regenerates the oxidized photoredox mediator (**II**→**III**) or it directly transfers an electron to a new α‐cyanoalkyl bromide substrate to initiate a chain reaction. Either way, rhodium‐coordinated product **IV** is formed and after product release (e.g. **7 a**) the coordination of new substrate leads to another catalytic cycle.

#### Mechanistic control experiments

**Scheme 3:**
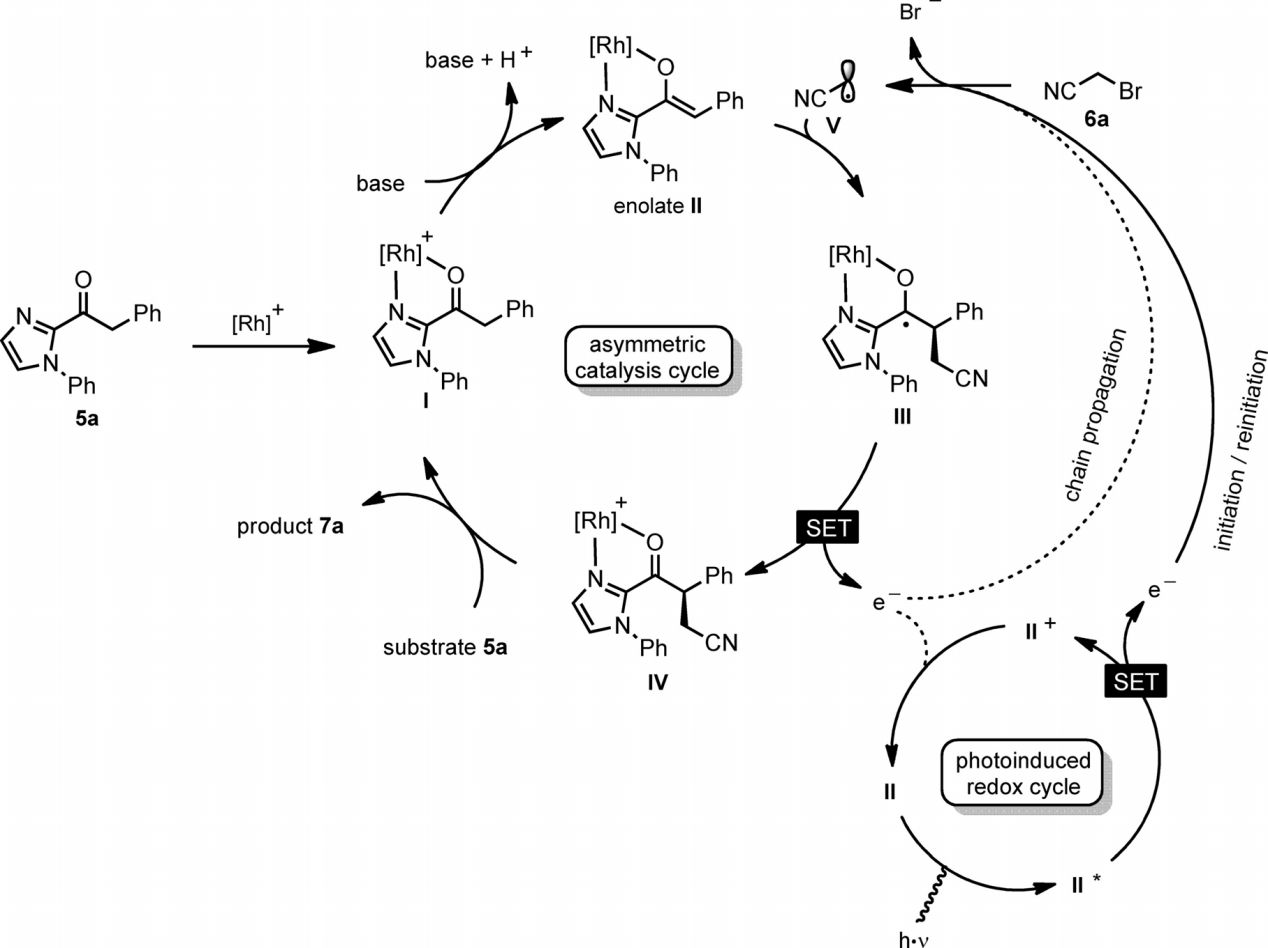
Proposed mechanism. [Rh]^+^ refers to the cationic, bis‐cyclometalated fragment of **RhInd**.

**Figure 5:**
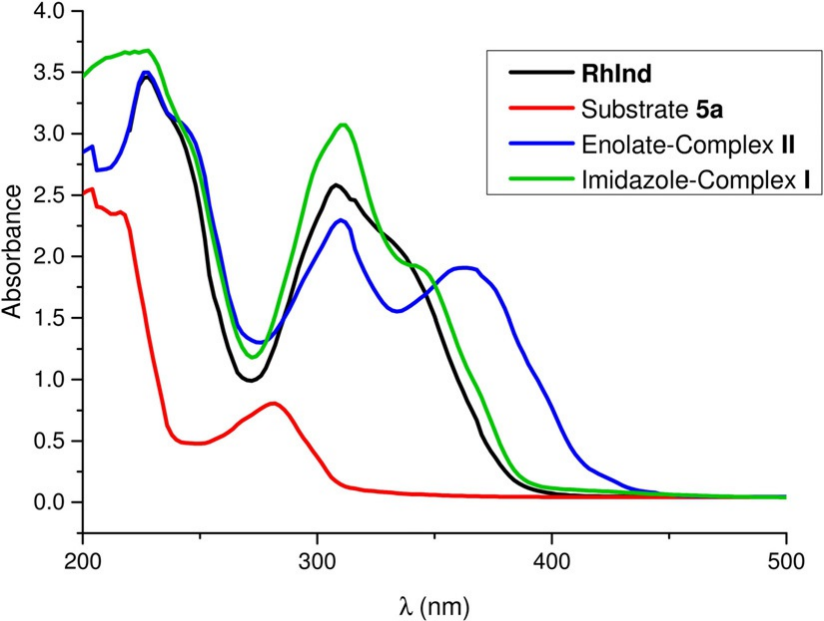
UV/Vis absorbance of **RhInd**, 2‐acyl imidazole **5 a**, imidazole‐complex **I**, and enolate‐complex **II** measured in CH_2_Cl_2_ (0.05 mm).

The proposed catalytic cycle is consistent with a number of control experiments. First, the reaction requires both catalyst and Brønsted base for achieving conversion (Table 2, entries 1 and 2), indicating the important role of the intermediate rhodium enolate (intermediate **II** in Scheme 3). Under air, the C−C coupling product is completely suppressed which is consistent with the interference of air with the proposed radical pathway, thus resulting in the formation of α‐keto‐2‐acyl imidazole as an undesired side product (entry 3). Without any visible light, only 20 % yield with significantly lower enantioselectivity was observed (entry 4). We propose that this product formation in the dark is the result of a non‐radical S_N_2‐pathway. When the photoreaction was performed in the presence of the radical trapping reagent (2,2,6,6‐tetramethylpiperidin‐1‐yl)oxyl (TEMPO), the yield dropped to 25 % (1.0 equiv TEMPO) and 20 % (6.0 equiv TEMPO), strongly indicating the involvement of a photoinduced radical mechanism (entries 5 and 6). A significant drop in enantioselectivity to 80 % is observed when H_2_O is excluded, demonstrating its crucial effect (entry 7). The benefit of small amounts of H_2_O can be rationalized with an improved solubility of the base Na_2_HPO_4_ in the reaction solvent. We also determined a quantum yield for this reactions, which is 0.046 for the reaction **5 a**+**6 a**→(*R*)‐**7 a**, which suggests that the chain propagation plays at most a minor role and instead the rhodium enolate complex **II** exerts the function of a real photoredox catalyst which is closely coupled to the asymmetric catalysis cycle. This is different from our previous iridium‐catalyzed α‐alkylations^2^, 9a, 20, 21 and rhodium‐catalyzed α‐aminations^2^, ^22^ which apparently follow a chain mechanism (quantum yields >1). Finally, UV/Vis absorption spectra shown in Figure 5 demonstrate that the 2‐acyl imidazole substrate **5 a**, the catalyst **RhInd**, and the rhodium ketone complex **I** are not capable of significantly absorbing visible light but that the rhodium enolate complex **II** after deprotonation of **I** features a new absorption band in the bathochromic region with a shoulder above 400 nm, which should be responsible for the visible‐light‐induced photochemistry. It also explains why the shorter wavelength of blue LEDs provides better results compared to a compact fluorescence light (CFL) bulb (entry 8).

**Table 2:** Comparison with other catalysts and control reactions.^[a]^

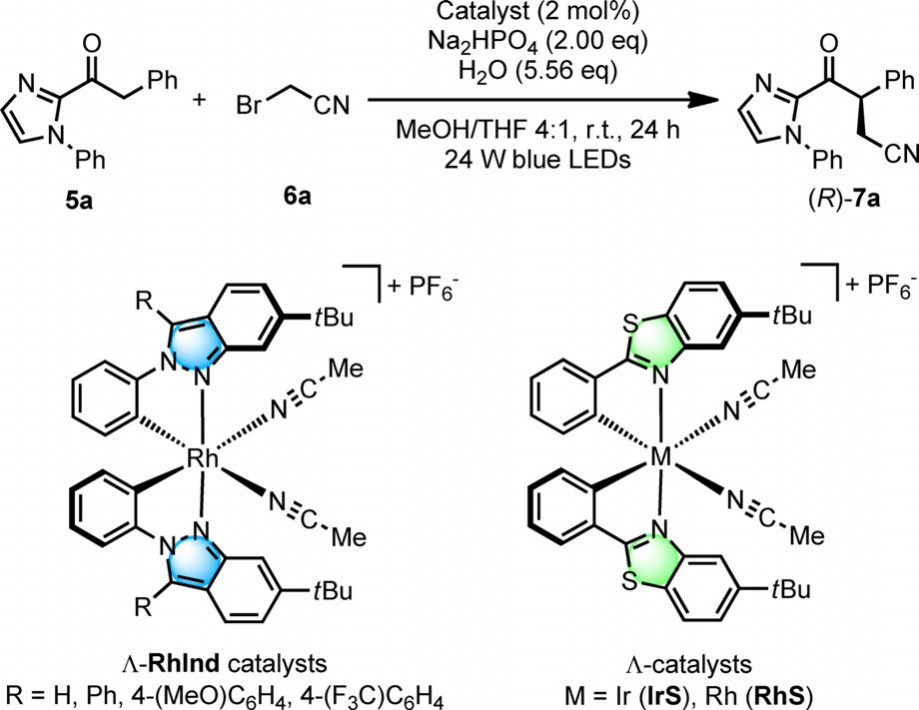				
Entry	Variations from standard procedure	Catalyst	Yield [%]	*ee* [%]^[b]^
1	without base	Λ‐RhInd	–	–
2	without catalyst	–	–	–
3	under air	Λ‐RhInd	–^[c]^	–
4	under air, without light	Λ‐RhInd	20	86
5	with 1.0 equiv TEMPO	Λ‐RhInd	25	14
6	with 6.0 equiv TEMPO	Λ‐RhInd	20	0
7	without H_2_O	Λ‐RhInd	77	80
8	CFL lamp	Λ‐RhInd	26	94
9	none	Λ‐RhInd	80	94
10	none	Λ‐IrS	23	94
11	50 °C	Λ‐IrS	62	0
12	none	Λ‐RhS	83	90
13	under air, without light	Λ‐RhS	38	87
14	none	Λ‐RhInd(Ph)^[d,e]^	73	74
15	none	*rac* RhInd(PhOMe)^[d,f]^	43	–
16	none	*rac* RhInd(PhCF_3_)^[d,g]^	13	–

[a] Conditions: 2‐Acyl imidazole (0.10 mmol), catalyst (2 mol %) and Na_2_HPO_4_ (0.20 mmol) were dissolved in MeOH/THF 4:1 (0.5 mL) under inert gas atmosphere and H_2_O (0.56 mmol) was added. The resulting mixture was stirred for 5 min before bromoacetonitrile (0.60 mmol) was added and the mixture was degassed via freeze‐pump‐thaw for three cycles. The reaction mixture was then stirred for 24 h under inert gas atmosphere at r.t. in front of blue LEDs (24 W, 10 cm). [b] Determined by chiral HPLC analysis. [c] Exclusive formation of α‐keto‐2‐acyl imidazole as side product. For more information, see Supporting Information. [d] For further information on modified catalysts see Supporting Information. [e] R=Ph. [f] R=4‐(MeO)C_6_H_4_. [g] R=4‐(F_3_C)C_6_H_4_.

#### Comparison with other catalysts

The performance of the new catalyst **RhInd** was compared with some related and previously reported bis‐cyclometalated complexes for the here introduced photoinduced cyanoalkylation. The bis‐cyclometalated phenylbenzothiazole complex Λ‐**IrS**, which was very successfully applied to a variety of enantioselective photoinduced α‐alkylations of 2‐acyl imidazoles,^2^, 9a, 20, 21 provided a high enantioselectivity of 94 % *ee* but with just 23 % yield (Table 2, entry 10). The low yield can be explained by the inhibition of the catalyst by blocking the active site of the catalyst through coordination of the bromoacetonitrile substrate or the cyanoalkylated product. This is consistent with the fact that the bis‐cyclometalated iridium catalyst displays a much slower ligand exchange kinetics compared to its rhodium congener and thus should be more sensitive to competing coordinating functional groups.^22^ Indeed, when we increased the reaction temperature to 50 °C to speed up ligand exchange, Λ‐**IrS** gave a significantly higher yield of 62 % but provided a racemic mixture of the product, which might be due to an uncatalyzed background reaction at higher temperatures (entry 11). On the other hand, the bis‐cyclometalated phenylbenzothiazole complex Λ‐**RhS**, which proved highly suitable for a variety of photoinduced α‐aminations of 2‐acyl imidazoles,^2^, ^10^, ^22^, ^23^ provided the cyanoalkylation product with 83 % yield but a slightly lower enantioselectivity of 90 % *ee* (entry 12). At a first glance, this lower enantioselectivity is surprising since the more constrained active site of the benzothiazole catalyst (see Figure 2 b) should provide a higher asymmetric induction. This is exactly what we observed for photoinduced α‐aminations of 2‐acyl imidazoles in which the benzothiazole catalyst **RhS** provided significantly higher *ee* values compared to the benzoxazole analogue **RhO**.10a We suggest that the higher enantioselectivity of **RhInd** over **RhS** for the photoinduced cyanoalkylation is due to a slower S_N_2 background catalysis with **RhInd**, and this is crucial because we observed a lower enantioselectivity for this reaction pathway. Indeed, in the presence of air and absence of light, Λ‐**RhS** provided the cyanoalkylation product in 38 % yield and with 88 % *ee* (entry 13), as compared to a yield of only 20 % with 86 % *ee* for Λ‐**RhInd** under the same conditions (entry 4). Finally, some modified **RhInd**‐catalysts were also tested but provided inferior results (entries 14–16).

## Conclusions

We here introduced a new chiral‐at‐metal rhodium(III) catalyst (**RhInd**) based on cyclometalated 2‐phenyl‐2*H*‐indazole ligands and developed a chiral‐auxiliary‐mediated synthesis of the individual Λ‐ and Δ‐enantiomer which provides virtually enantiopure complexes (*ee*>99 %). The new 2‐phenylindazole complex is structurally related to previously reported rhodium(III) catalysts containing cyclometalated 2‐phenylbenzoxazole and 2‐phenylbenzothiazole ligands. However, the indazole ligand apparently provides **RhInd** with distinct catalytic properties as demonstrated for an efficient visible‐light‐induced asymmetric α‐cyanoalkylation of 2‐acyl imidazoles. This cyanoalkylation complements previous photoredox‐mediated α‐alkylations using electron deficient benzyl bromides, phenacyl bromides, perfluoroalkyl halides, and enantioselective trichloromethylations with BrCCl_3_.^2^, 9a, 20, 21, 26 Especially branched α‐cyano bromides afford promising results with high enantioselectivities. Future work will investigate other applications of **RhInd** as a dual function catalyst for asymmetric photochemistry.

## Experimental Section

### General procedure for enantioselective α‐alkylation of 2‐acyl imidazoles

2‐Acyl imidazole (0.10 mmol), Λ‐**RhInd** (2.00 mol %) and Na_2_HPO_4_ (0.20 mmol) were dissolved in MeOH/THF 4:1 (0.5 mL) under inert gas atmosphere and H_2_O (0.56 mmol) was added. The resulting mixture was stirred for 5 min before bromoacetonitrile (0.60 mmol) was added and the mixture was thoroughly degassed via freeze‐pump‐thaw for three cycles. The reaction mixture was then stirred for 24 h under inert gas atmosphere at r.t. in front of blue LEDs (24 W, 10 cm). Afterwards, the solvent was evaporated under vacuum and the precipitate was purified by column chromatography on silica gel (*n*‐pentane/EtOAc 5:1→2:1) to afford pure products. For compounds **7 k**–**m** diastereomeric ratios were determined by ^1^H NMR spectroscopy of the crude products.

## Conflict of interest

The authors declare no conflict of interest.

## Supporting information

As a service to our authors and readers, this journal provides supporting information supplied by the authors. Such materials are peer reviewed and may be re‐organized for online delivery, but are not copy‐edited or typeset. Technical support issues arising from supporting information (other than missing files) should be addressed to the authors.

Supplementary
