# Supplementary material for: Bis‐Cyclometalated Indazole Chiral‐at‐Rhodium Catalyst for Asymmetric Photoredox Cyanoalkylations
Source: Chemistry. 2019 Nov 13;25(67):15333–40. doi: 10.1002/chem.201903369 (PMC6916287; doi:10.1002/chem.201903369)
Supplement: Supplementary file 1 — Supplementary [file CHEM-25-15333-s001.pdf]

CHEMISTRY
A European Journal

Supporting Information

Bis-Cyclometalated Indazole Chiral-at-Rhodium Catalyst for Asymmetric Photoredox Cyanoalkylations

Philipp S. Steinlandt, Wei Zuo, Klaus Harms, and Eric Meggers*[a]

chem_201903369_sm_miscellaneous_information.pdf

Table of Contents

1 General Information **S2**

2 Synthesis of RhInd Catalyst **S3**

2.1 Synthesis of 2*H*-Indazole Ligand **1** S3

2.2 Auxiliary-Mediated Synthesis of Enantiopure Λ- and Δ-**RhInd** S5

2.3 CD Spectra S9

2.4 HPLC Traces S10

2.5 UV/VIS Absorbance of **RhInd** S12

3 α-Cyanomethylation of 2-Acyl Imidazoles **S13**

3.1 Substrate Synthesis S13

3.2 Optimization of Reaction Conditions S13

3.3 Substrate Scope of α-Cyanomethylation of 2-Acyl Imidazoles S16

4 Mechanistical Studies **S26**

4.1 Control Reactions S26

4.2 Racemization Control Experiments S27

4.3 Synthesis and Characterization of RhInd-Enolate Complex **II** S28

4.4 Quantum Yield Determination S30

5 NMR Spectra **S32**

5.1 NMR Spectra of Catalyst Synthesis S32

5.2 NMR Spectra of Substrate Scope for α-Cyanomethylation S39

6 Chiral HPLC Traces **S51**

7 Single Crystal X-Ray Diffraction **S63**

8 References **S67**

1 General Information

All reactions were carried out under an atmosphere of nitrogen with magnetic stirring in flame-dried glassware unless stated otherwise. Catalytic reactions were performed in Schlenk tubes (10 mL), placed 10 cm away from a 24 W blue LED lamp (Hongchangzhaoming from Chinese Taobao, https://hongchang-led.taobao.com, see Supplementary Figure 1 in our previous publication[1]). Prior to irradiation, the reactions mixtures were degassed thoroughly via freeze-pump-thaw for three cycles over the course of 45 min. Solvents were distilled under nitrogen from sodium/benzophenon (THF, Et_2O) or calcium hydride (MeCN, CH_2Cl_2, $CHCl_3$, toluene). Commercially purchased compounds were used without further purification. Flash column chromatography was performed with silica gel 60 M from Macherey-Nagel (irregular shaped, 230-400 mesh, pH 6.8, pore volume: 0.81 mL x g-1, mean pore size: 66 A, specific surface: 492 m2 x g-1, particle size distribution: 0.5% < 25 µm and 1.7% > 71 µm, water content: 1.6%). ^{1}H NMR, ^{13}C{1H} NMR and ^{19}F{1H, 13C} NMR spectra were recorded on a Bruker AV II 300 MHz, AV III HD 250 MHz, AV III 500 MHz, AV III HD 500 MHz or AV II 600 MHz spectrometer at room temperature. The chemical shift δ is listed in ppm with the solvent resonance as internal standard. IR spectra were acquired on a Bruker Alpha FT-IR spectrophotometer. The resulting absorption bands are listed as wavenumbers (cm^{-1}) and the correlating intensities are specified as w (weak), s (strong) or m (medium). High-resolution mass spectrometry was measured via electrospray-ionization-technique (ESI) or atmospheric pressure chemical ionization-technique (APCI) on a Finnigan LTQ-FT Ultra mass spectrometer (Thermo Fischer Scientific). CD spectra were recorded on JASCO J-810 CD spectropolarimeter (parameters: 600-200 nm, bandwidth 1 nm, data pitch 0.5 nm, response 1 second, sensitivity standard, scanning speed 50 nm/min, accumulation of 3 scans). Optical rotations were measured on a Krüss P8000-T polarimeter with $[\alpha]_D^{22}$ values listed in degrees with concentrations reported in g/100 mL.

2 Synthesis of RhInd Catalyst

2.1 Synthesis of 2*H*-Indazole Ligand 1

Starting from compound **S1**, benzaldehyde **S4** was prepared according to published procedures.[2] The synthetic route for the synthesis of 2*H*-indazole ligand **1** is shown in Scheme S1.

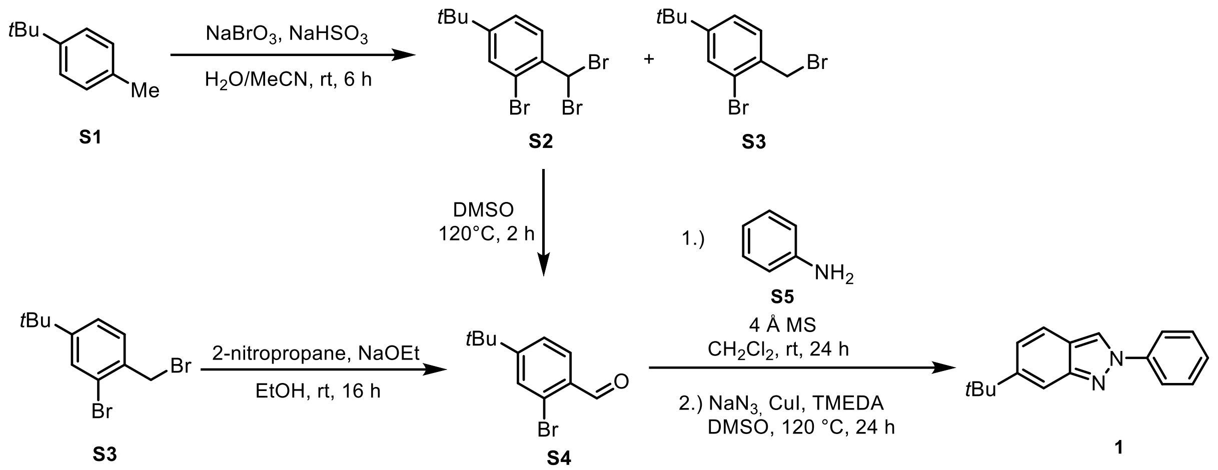

Scheme S1. Synthetic pathway to 2*H*-indazole ligand **1**.

6-(*tert*-Butyl)-2-phenyl-2*H*-indazole (1)

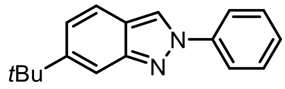

1

According to a procedure by OH *et al.*,[3] a flame dried SCHLENK flask was charged with benzaldehyde **S4** (2.60 g, 10.8 mmol, 1.00 eq.), aniline (**S5**, 0.98 mL, 10.8 mmol, 1.00 eq.) and molecular sieve (4 Å, 1 g/mmol of benzaldehyde **S4**). CH_2Cl_2 (53.0 mL) was added and the resulting mixture was stirred at r.t. for 24 h. Afterwards, the mixture was filtered through a short plug of Celite and the filtrate was concentrated under reduced pressure to give a yellow solid. Subsequently, the solid was dissolved in DMSO (50.0 mL) and CuI (412 mg, 2.16 mmol, 20 mol%), tetramethylethylendiamine (TMEDA, 326 µL, 2.16 mmol, 20 mol%) and NaN_3

(1.41 g, 21.6 mmol, 2.00 eq.) were added. The resulting mixture was stirred for 24 h at 120 °C. Afterwards, the brown mixture was cooled to r.t., diluted with EtOAc and H_2O (40 mL each) and filtered through a short plug of Celite. Layers were separated and the aqueous layer was extracted with EtOAc (3 x 40 mL). The combined organic layers were washed with sat. aq. NaCl-solution (120 mL), dried over Na_2SO_4, filtered and the solvent was removed under reduced pressure. The crude product was purified by column chromatography on silica gel (*n*-pentane/EtOAc 30:1). After recrystallization from *n*-hexane, 2*H*-indazole ligand **1** (1.68 g, 6.71 mmol, 62%) was obtained as pale yellow crystals.

TLC (*n*-pentane/EtOAc 30:1): $R_f = 0.35$. **^{1}H NMR** (500 MHz, CD_2Cl_2): $\delta = 8.39$ (d, $J = 0.9$ Hz, 1H), 7.93-7.90 (m, 2H), 7.68-7.67 (m, 1H), 7.65 (dd, $J = 8.8$, 0.8 Hz, 1H), 7.56-7.51 (m, 2H), 7.42-7.38 (m, 1H), 7.25 (dd, $J = 8.9$, 1.6 Hz, 1H), 1.40 (s, 9H) ppm. **^{13}C NMR** (126 MHz, CD_2Cl_2): $\delta = 150.8$, 150.3, 141.1, 129.9 (2C), 127.9, 122.6, 121.4, 120.9 (2C), 120.2, 120.1, 112.7, 35.5, 31.2 (3C) ppm. **IR** (neat): $\tilde{\nu} = 3131$ (w), 3060 (w), 2975 (w), 2953 (m), 2902 (w), 2866 (w), 1733 (w), 1633 (w), 1593 (m), 1505 (m), 1463 (m), 1383 (m), 1363 (w), 1328 (w), 1297 (w), 1230 (w), 1196 (w), 1153 (w), 1099 (w), 1073 (w), 1044 (m), 1022 (w), 940 (w), 906 (w), 868 (w), 842 (w), 809 (s), 754 (s), 728 (m), 706 (w), 683 (m), 657 (m), 625 (w), 598 (w), 505 (m), 454 (w), 423 (w) cm^{-1}. **HRMS (ESI)** *m*/*z* calcd. for $C_{17}H_{19}N_2$ $[M + H^+]$: 251.1543, found: 251.1541. **m.p.**: 76°C (EtOAc).

2.2 Auxiliary-Mediated Synthesis of Enantiopure Λ- and Δ-RhInd

Synthesis of *rac*-RhInd

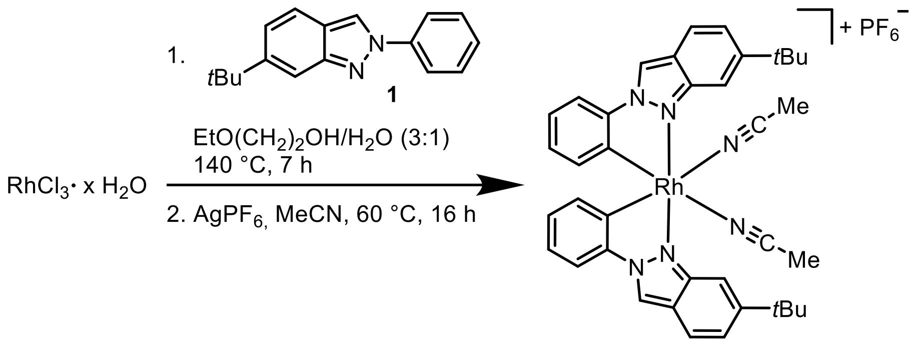

rac-**RhInd**

Following a slightly modified procedure of MEGGERS *et al.*,[4] $RhCl_3 \cdot x\ H_2O$ (w_{Rh} = 40%, 150 mg, 0.58 mmol, 1.00 eq.) and 2*H*-indazole ligand **1** (292 mg, 1.17 mmol, 2.00 eq.) were added to a SCHLENK flask. Subsequently, a mixture of 2-ethoxyethanol and H_2O (v/v = 3:1, 11.7 mL) was added and the resulting solution was stirred at 140 °C for 7 h. Afterwards, the solvent was removed thoroughly under reduced pressure and the precipitate was dried under vacuum. $AgPF_6$ (301 mg, 1.17 mmol, 2.00 eq.) and MeCN (11.7 mL) were added and the resulting mixture was stirred for 16 h at 60 °C. After cooling to r.t., the mixture was filtered through a short plug of Celite and rinsed with MeCN. The solvent was removed under reduced pressure and the crude product was purified on silica gel by column chromatography (CH_2Cl_2/MeCN 20:1 → 10:1). After recrystallization from CH_2Cl_2/*n*-hexane, *rac*-**RhInd** (470 mg, 0.56 mmol, 97%) was obtained as pale yellow crystals.

TLC (CH_2Cl_2/MeCN 10:1): R_f = 0.30. **^{1}H NMR** (500 MHz, MeCN-d_3): δ = 9.01 (s, 2H), 7.97 (s, 2H), 7.91 (d, J = 9.0 Hz, 2H), 7.62 (d, J = 7.6 Hz, 2H), 7.54 (dd, J = 9.0, 1.3 Hz, 2H), 7.03 (t, J = 7.6 Hz, 2H), 6.70 (t, J = 7.5 Hz, 2H), 5.79 (d, J = 7.8 Hz, 2H), 2.16 (s, 6H), 1.44 (s, 18H) ppm. **^{13}C NMR** (126 MHz, MeCN-d_6): δ = 154.5 (2C), 149.1 (2C), 145.8 (d, $J_{Rh,C}$ = 32.5 Hz, 2C), 143.3 (2C), 134.7 (2C), 128.0 (2C), 125.4 (2C), 124.4 (2C), 122.6 (2C), 122.5 (2C), 121.8 (2C), 114.8 (2C), 110.2 (2C), 36.3 (2C), 31.1 (6C), 1.8 (2C) ppm. **^{19}F NMR**

(282 MHz, MeCN-d_3): δ = –72.5 (d, $J_{P,F}$ = 706.3 Hz, 6F) ppm. **IR** (neat): $\tilde{\nu}$ = 3148 (w), 3062 (w), 2958 (w), 2870 (w), 2280 (w), 1631 (w), 1494 (w), 1459 (w), 1384 (w), 1365 (w), 1281 (w), 1253 (w), 1234 (w), 1202 (w), 1148 (w), 1122 (w), 1075 (w), 1048 (w), 1026 (w), 983 (w), 947 (w), 838 (s), 744 (w), 660 (w), 629 (w), 556 (m), 458 (w) cm^{-1}. **HRMS (APCI)**: *m*/*z* calcd. for $C_{36}H_{37}N_5Rh$ $[M - MeCN]^+$: 642.2099, found: 642.2113. **m.p.**: 221 °C (DCM).

Synthesis of Auxiliary Complexes Λ-(*S*)-3 and Δ-(*S*)-4

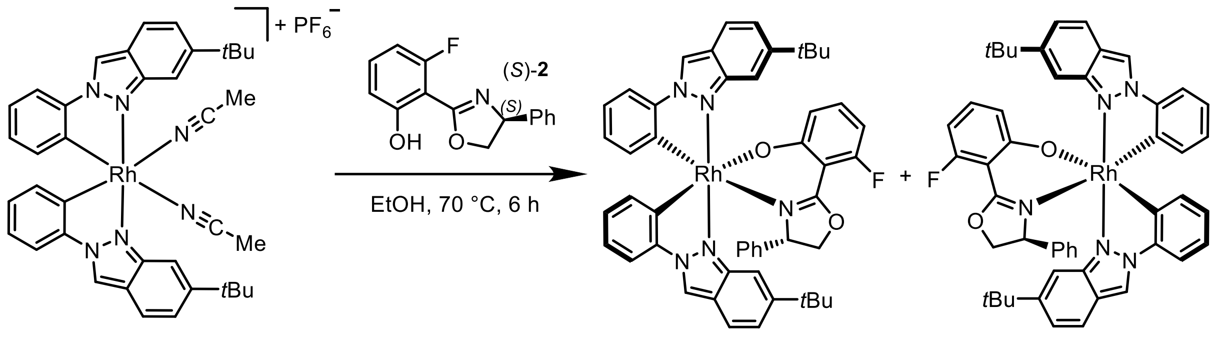

According to a procedure by MEGGERS *et al.*,[5] *rac*-**RhInd** (150 mg, 0.18 mmol, 1.00 eq.), (*S*)-auxiliary **2** (51.2 mg, 0.20 mmol, 1.10 eq.) and K_2CO_3 (75.1 mg, 0.54 mmol, 3.00 eq.) were dissolved in absolute ethanol (7.00 mL) and stirred for 6 h at 70 °C. Afterwards, the mixture was diluted with CH_2Cl_2 (20 mL), filtered through a short plug of Celite and rinsed with CH_2Cl_2 (20 mL). After removing the solvent under reduced pressure, the crude product was purified by column chromatography on silica gel (*n*-pentane/EtOAc + 1% Et_3N 10:1 → 2:1) to afford Λ-(*S*)-**3** (62.0 mg, 72.3 µmol, 40%) and Δ-(*S*)-**4** (74.0 mg, 86.3 µmol, 48%) both as yellow solids.

Λ-(*S*)-**3**:

TLC (*n*-pentane/EtOAc 2:1 + 1% Et_3N): R_f = 0.48. **^{1}H NMR** (500 MHz, CD_2Cl_2): δ = 8.58 (s, 1H), 8.15 (s, 1H), 7.85 (s, 1H), 7.69 (d, *J* = 9.0 Hz, 1H), 7.55 (s, 1H), 7.47 (d, *J* = 9.0 Hz, 1H), 7.38 (d, *J* = 7.9 Hz, 1H), 7.34 (dd, *J* = 9.0, 1.4 Hz, 1H), 7.27 (dd, *J* = 9.0, 1.5 Hz, 1H), 7.04 (d, *J* = 7.1 Hz, 1H), 6.97-6.88 (m, 3H), 6.77 (t, *J* = 7.4 Hz, 1H), 6.71 (dt, *J* = 7.4, 1.1 Hz, 1H), 6.63

(dt, J = 7.5, 1.0 Hz, 1H), 6.51 (bs, 2H), 6.45 (d, J = 8.8 Hz, 1H), 6.34 (d, J = 7.5 Hz, 1H), 6.20 (d, J = 7.1 Hz, 2H), 5.93 (ddd, J = 12.8, 7.8, 0.9 Hz, 1H), 5.59 (d, J = 7.7 Hz, 1H), 4.90-4.85 (m, 1H), 4.84-4.79 (m, 1H), 4.04-4.01 (m, 1H), 1.44 (s, 9H), 1.28 (s, 9H) ppm. **^{13}C NMR** (126 MHz, CD_2Cl_2): δ = 174.8 (d, $J_{F,C}$ = 3.4 Hz), 165.7 (d, $J_{F,C}$ = 3.5 Hz), 164.0 (d, $J_{F,C}$ = 257.0 Hz), 154.1 (d, $J_{Rh,C}$ = 30.9 Hz), 152.8, 152.2, 151.3 (d, $J_{Rh,C}$ = 32.2 Hz), 148.8, 148.4, 143.6, 143.1, 140.9, 135.6, 134.8, 132.9 (d, $J_{F,C}$ = 14.1 Hz), 129.2, 127.8 (2C), 126.9, 126.8, 126.4, 125.7, 123.2, 123.0 (2C), 122.7, 121.0, 120.9, 120.9, 120.7 (d, $J_{F,C}$ = 3.0 Hz), 120.1, 119.5, 118.9, 113.4, 113.2, 111.6, 109.5, 100.6 (d, $J_{F,C}$ = 6.1 Hz), 98.6 (d, $J_{F,C}$ = 24.3 Hz), 75.2, 69.6, 35.8, 35.6, 31.1 (6C) ppm. **^{19}F NMR** (283 MHz, CD_2Cl_2): δ = –105.0 ppm. **IR** (neat): $\tilde{\nu}$ = 3327 (w), 3144 (w), 3051 (w), 2959 (w), 2534 (w), 2478 (w), 2456 (w), 2372 (w), 2336 (w), 2282 (w), 2242 (w), 2220 (w), 2196 (w), 2170 (w), 2121 (w), 2077 (w), 2011 (w), 1976 (w), 1941 (w), 1867 (w), 1771 (w), 1616 (m), 1581 (w), 1526 (w), 1487 (w), 1444 (s), 1366 (m), 1320 (w), 1278 (w), 1249 (w), 1215 (m), 1154 (w), 1119 (w), 1091 (w), 1062 (w), 1026 (m), 1001 (w), 985 (w), 946 (w), 923 (w), 892 (w), 842 (s), 815 (m), 790 (m), 772 (w), 743 (s), 696 (m), 659 (w), 627 (w), 611 (w), 579 (w), 559 (w), 531 (m), 482 (w), 459 (m), 440 (w) cm^{-1}. **HRMS (APCI)**: *m*/*z* calcd. for $C_{49}H_{46}FN_5O_2Rh$ [M + H$^+$]: 858.2685, found: 858.2693. **CD** (MeOH): λ, nm ($\Delta\varepsilon$ in M^{-1}cm^{-1}): 384 (–13), 342 (+46), 300 (–48), 274 (–16), 258 (–25), 244 (+6), 235 (–17). **m.p.**: 299 °C decomposition (EtOAc).

Δ-(*S*)-**4**:

TLC (*n*-pentane/EtOAc 2:1 + 1% NEt_3): R_f = 0.35. **^{1}H NMR** (300 MHz, CD_2Cl_2): δ = 8.65 (s, 1H), 8.54 (s, 1H), 8.31 (s, 1H), 7.81-7.77 (m, 2H), 7.63 (d, J = 9.0 Hz, 1H), 7.42-7.36 (m, 2H), 7.29 (dd, J = 9.0, 1.1 Hz, 1H), 7.18 (d, J = 7.9 Hz, 1H), 6.95 (t, J = 7.5 Hz, 1H), 6.93-6.88 (m, 1H), 6.85 (t, J = 7.4 Hz, 2H), 6.81-6.76 (m, 3H), 6.70–6.63 (m, 2H), 6.26 (d, J = 8.7 Hz, 1H), 6.19 (t, J = 7.4 Hz, 1H), 5.97-5.89 (m, 2H), 5.62 (d, J = 7.7 Hz, 1H), 4.26 (t, J = 9.4 Hz, 1H), 4.03 (t, J = 9.4 Hz, 1H), 3.77 (t, J = 10.0 Hz, 1H), 1.39 (s, 9H), 1.25 (s, 9H) ppm. **^{13}C NMR**

(126 MHz, CD_2Cl_2): δ = 174.5 (d, $J_{F,C}$ = 3.9 Hz), 166.2, 163.2 (d, $J_{F,C}$ = 254.0 Hz), 153.1 (d, $J_{Rh,C}$ = 31.9 Hz), 152.8, 152.1, 151.3 (d, $J_{Rh,C}$ = 32.3 Hz), 148.8, 148.8, 142.9, 142.6, 140.2, 136.4, 135.0, 132.6 (d, $J_{F,C}$ = 13.7 Hz), 128.3 (2C), 127.5, 127.2 (2C), 126.8, 126.4, 123.7, 123.2, 123.0, 122.5, 121.0, 120.6, 120.3, 119.5 (d, $J_{F,C}$ = 2.9 Hz), 119.2, 118.7, 113.2, 112.9, 111.2, 110.7, 103.1 (d, $J_{F,C}$ = 8.2 Hz), 98.5 (d, $J_{F,C}$ = 22.3 Hz), 75.4, 70.4, 35.8, 35.7, 31.1 (3C), 30.9 (3C) ppm. **^{19}F NMR** (283 MHz, CD_2Cl_2): δ = –107.8 ppm. **IR** (neat): $\tilde{\nu}$ = 3357 (w), 3054 (w), 2960 (m), 2868 (w), 2381 (w), 2311 (w), 2281 (w), 2213 (w), 2186 (w), 2141 (w), 2114 (w), 2021 (w), 1985 (w), 1964 (w), 1933 (w), 1897 (w), 1618 (s), 1595 (w), 1581 (w), 1530 (w), 1486 (w), 1446 (s), 1364 (m), 1279 (w), 1259 (w), 1220 (m), 1149 (w), 1092 (w), 1067 (w), 1025 (s), 980 (w), 947 (w), 922 (w), 845 (w), 809 (m), 791 (w), 741 (s), 694 (m), 657 (w), 629 (w), 580 (w), 558 (w), 530 (w), 494 (w), 459 (w) cm^{-1}. **HRMS (APCI)**: *m*/*z* calcd. for $C_{49}H_{46}FN_5O_2Rh$ [M + H$^+$]: 858.2685, found: 858.2676. **CD** (MeOH): λ, nm ($\Delta\varepsilon$ in $M^{-1}cm^{-1}$): 382 (+25), 364 (+16), 333 (–22), 299 (+48), 276 (+15), 263 (+22), 248 (–14), 234 (+20). **m.p.**: 205 °C (EtOAc).

Synthesis of Enantiopure Λ-and Δ-RhInd

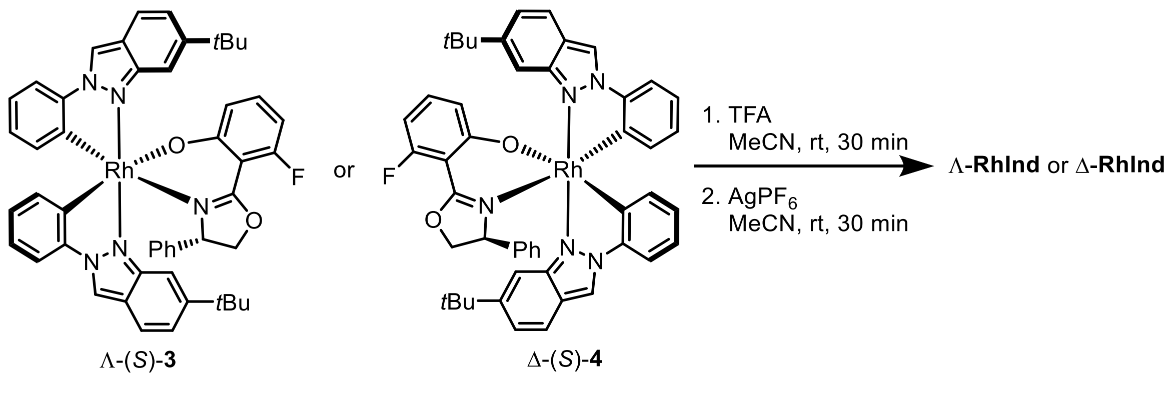

To a suspension of Λ-(*S*)-**3** (50.0 mg, 58.0 µmol, 1.00 eq.) or Δ-(*S*)-**4** (50.0 mg, 58.0 µmol, 1.00 eq.) in MeCN (1.45 mL) was added TFA (27.0 µL, 0.35 mmol, 6.00 eq.) in one portion and stirred for 30 min at r.t. under air. Subsequently, the reaction mixture was evaporated under vacuum and the precipitate was redissolved in MeCN (1.45 mL). NH_4PF_6 (190 mg, 1.17 mmol,

20.0 eq.) was added and stirred for another 30 min at r.t. under air. Afterwads, the mixture was concentrated and subjected to column chromatography on silica gel (CH_2Cl_2/MeCN 20:1 → 10:1). Recrystallization from CH_2Cl_2/*n*-hexane gave enantiopure Λ-**RhInd** (46.0 mg, 55.5 μmol, 95%) or Δ-**RhInd** (42.0 mg, 50.7 μmol, 87%) as pale yellow crystals.

All other analytical data of enantiopure **RhInd** catalysts were in agreement with the racemic catalyst.

Λ-**RhInd**: **CD** (MeOH): λ, nm (Δε in $M^{-1}cm^{-1}$): 375 (–16), 362 (–7), 338 (+35), 296 (–54), 270 (–16), 238 (+78), 233 (+72), 221 (+94), 213 (+12).

Δ-**RhInd**: **CD** (MeOH): λ, nm (Δε in $M^{-1}cm^{-1}$): 375 (+16), 362 (+8), 338 (–32), 296 (+55), 270 (+18), 236 (–72), 232 (–68), 222 (–90), 212 (–12).

2.3 CD Spectra

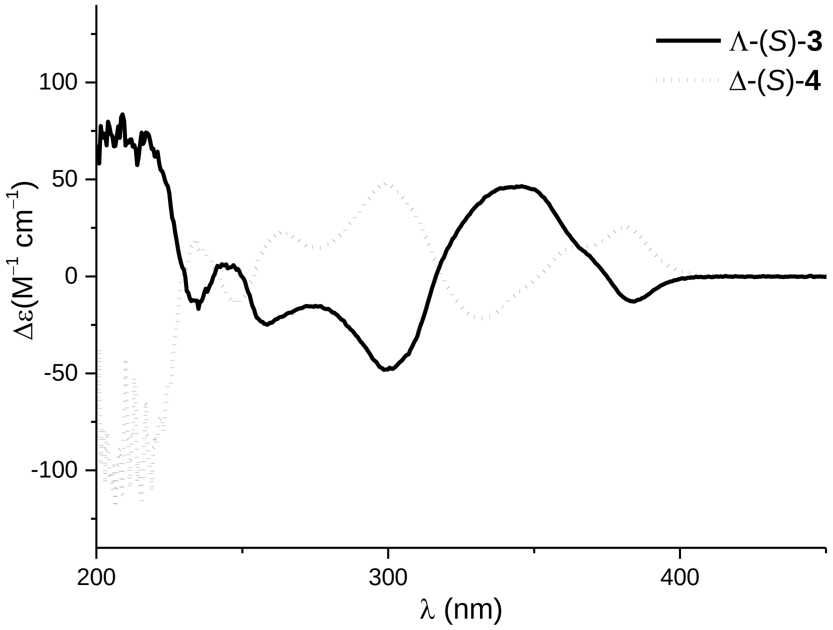

Figure S1. CD spectra (0.2 mM in MeOH) of Λ-(*S*)-**3** and Δ-(*S*)-**4**.

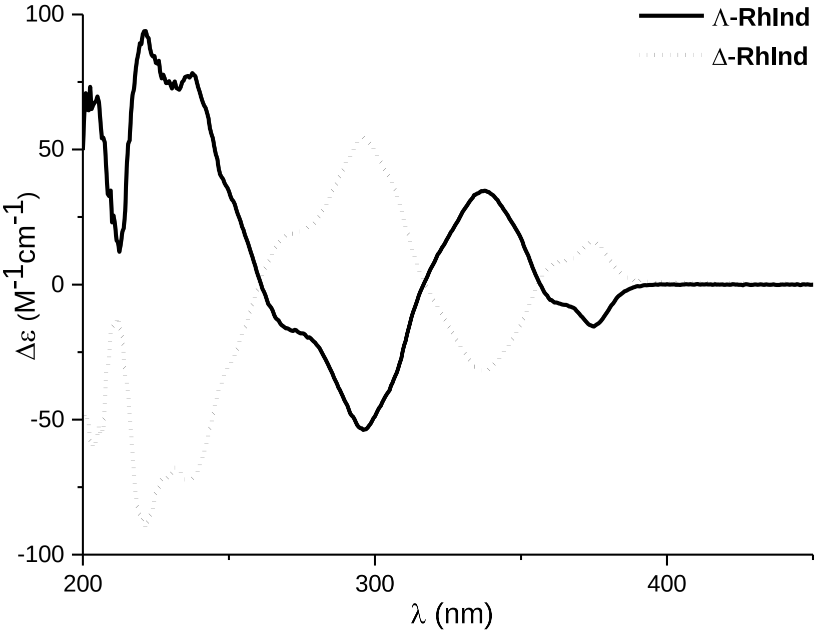

Figure S2. CD spectra (0.2 mM in MeOH) of Λ- and Δ-**RhInd**.

2.4 HPLC Traces

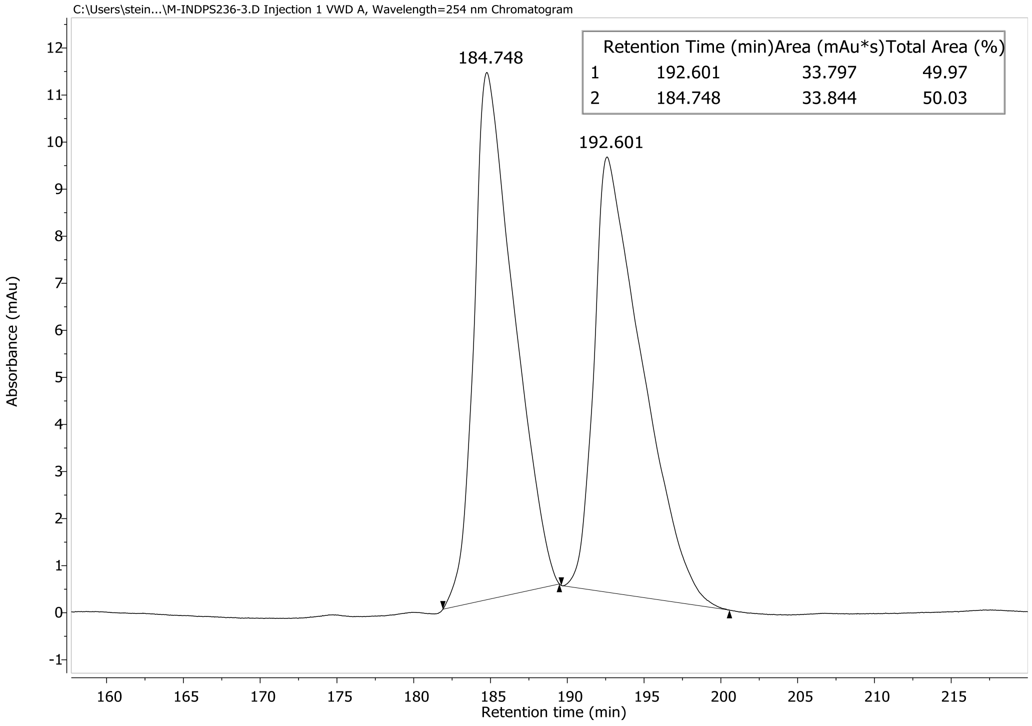

Figure S3. HPLC trace of *rac*-**RhInd**.

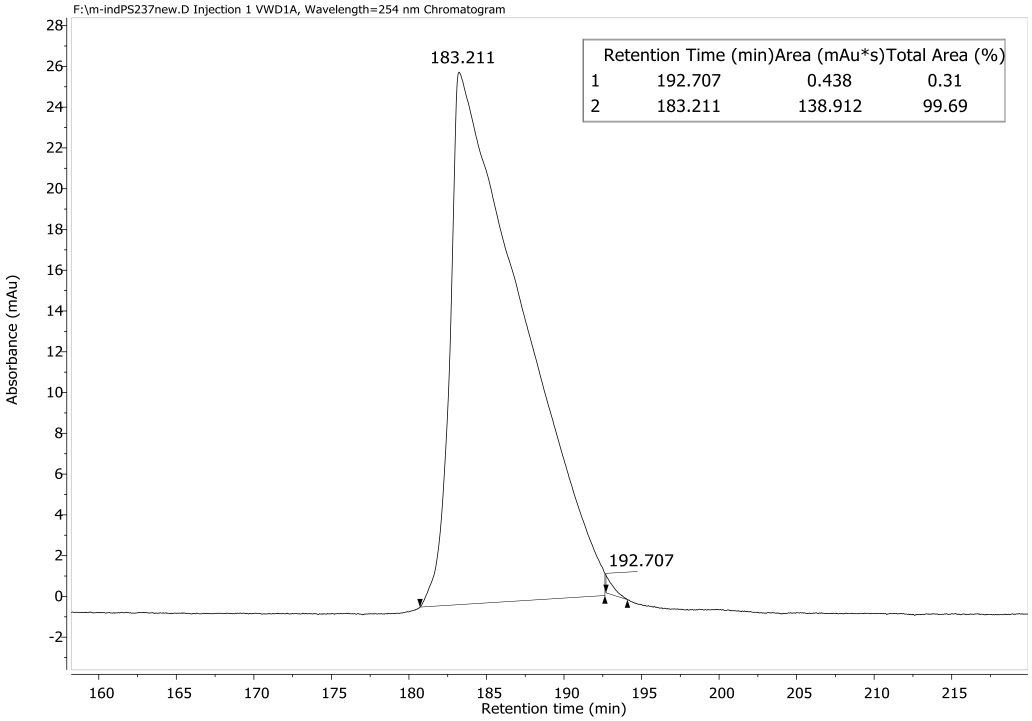

Figure S4. HPLC trace of Δ-**RhInd** (99.4% *ee*).

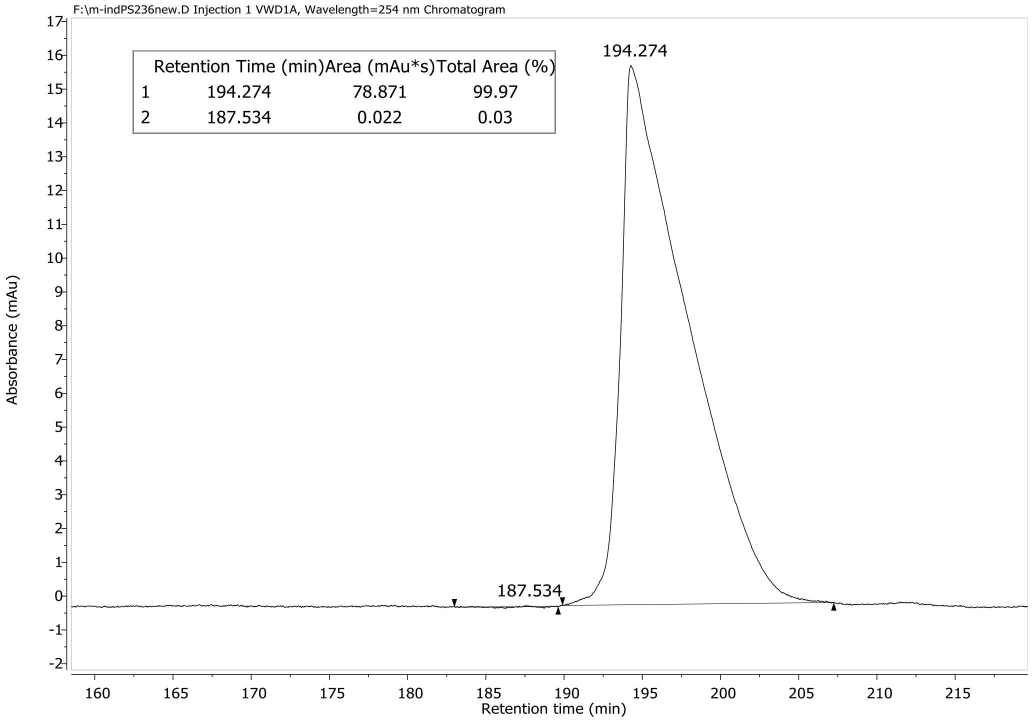

Figure S5. HPLC trace of Λ-**RhInd** (99.9% *ee*).

2.5 UV/VIS Absorbance of RhInd

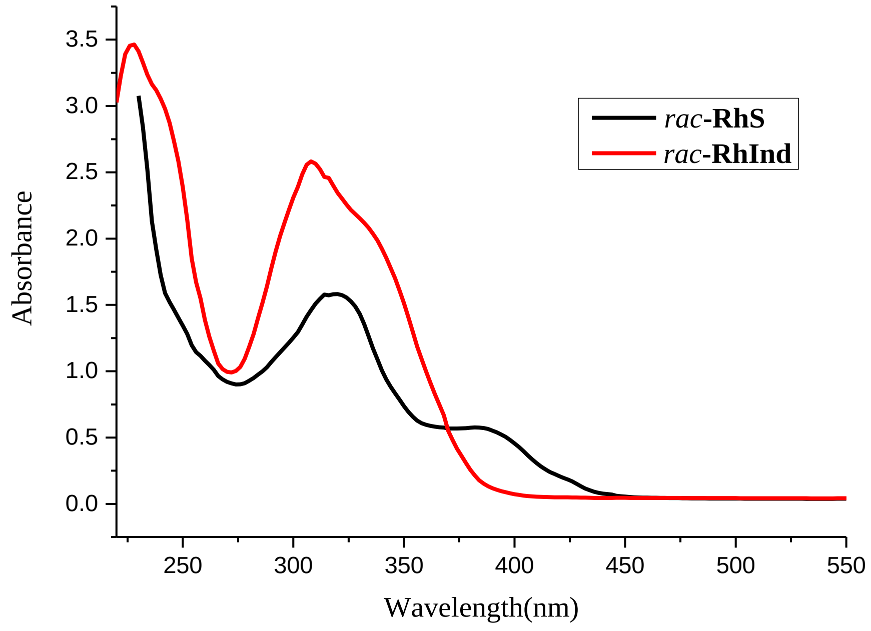

Figure S6. UV/VIS absorbance spectra of *rac*-**RhS** (black) and *rac*-**RhInd** (red) in CH_2Cl_2 (0.05 mM).

3 α-Cyanomethylation of 2-Acyl Imidazoles

3.1 Substrate Synthesis

2-Acyl imidazole substrates **5a-j** were synthesized according to a published procedure.[6] Analytical data for substrates **5a**, **5d**, **5e**, **5i**, and **5j**[7] and for substrates **5b**, **5f**, **5g**, and **5h**[8] are in agreement with the literature. Bromoacetonitrile **6a** was purchased and used without further purification. α-Cyano bromides **6b**,[9] **6c**,[10], **6d**[11], **6e**[12], and **6f**[13] were prepared according to published procedures.

3.2 Optimization of Reaction Conditions

General Procedure for Optimization of Reaction Conditions

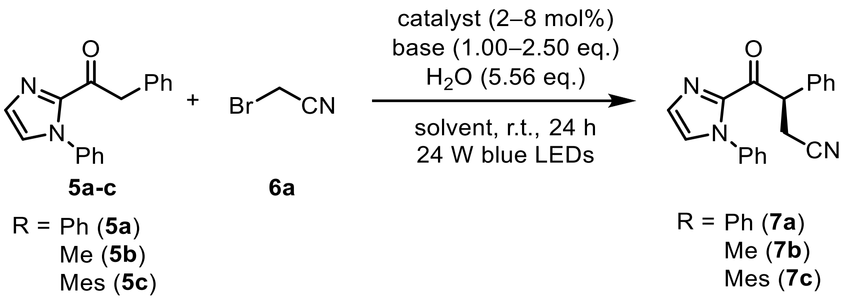

A 10 mL Schlenk tube was charged with 2-acyl imidazole **5a-c**, catalyst and the indicated base and put under inert gas atmosphere. The indicated solvent and H_2O were added and the resulting mixture was stirred for 5 min before bromoacetonitrile (**6a**) was added and, subsequently, degassed via freeze-pump-thaw thoroughly for three cycles. The reaction mixture was then stirred for 24 h under inert gas atmosphere at r.t. in front of blue LEDs (24 W, 10 cm). Afterwards, the solvent was evaporated under vacuum and the precipitate was purified by column chromatography on silica gel (*n*-pentane/EtOAc 5:1 → 2:1) to afford pure product **7a-c**.

Table S1. Survey of solvents.[a]

Entry	Solvent	Yield [%][b]	*ee* [%][c]
**1**	**MeOH/THF 4:1**	**22**	**94**
2	MeCN	–	–
3	THF	–	–
4	MeOH	15	80
5	MeOH/CH_2Cl_2 1:1	17	79
6	MeOH/MeCN 4:1	–	–

[a] Reaction conditions: According to the general procedure, **5a** (0.10 mmol), Λ-**RhInd** (2 mol%) and Na_2HPO_4 (0.11 mmol) were dissolved in the indicated solvent (0.50 mL). After addition of H_2O (5.56 mmol) and **6a** (0.30 mmol), the reaction mixture was degassed and stirred for 24 h at r.t. in front of blue LEDs under an atmosphere of nitrogen. [b] Isolated yield. [c] Determined by chiral HPLC analysis.

TableS 2. Survey of base.[a]

Entry	Base	Yield [%][b]	*ee* [%][c]
**1**	**Na_2HPO_4**	**22**	**94**
2	2,6-Lutidine	50	87
3	Cs_2CO_3	73	4
4	DIPEA	2	42

[a] Reaction conditions: According to the general procedure, **5a** (0.10 mmol), Λ-**RhInd** (2 mol%) and the indicated base (0.11 mmol) were dissolved in MeOH/THF 4:1 (0.50 mL). After addition of H_2O (5.56 mmol) and **6a** (0.30 mmol), the reaction mixture was degassed and stirred for 24 h at r.t. in front of blue LEDs under an atmosphere of nitrogen. [b] Isolated yield. [c] Determined by chiral HPLC analysis.

Table S3. Survey of catalyst loading and amount bromoacetonitrile (**6a**).[a]

Entry	Catalyst Loading [mol%]	Equivalents of **6a**	Yield [%][b]	*ee* [%][c]
1	2	3.00	22	94
2	4	3.00	47	78
3	8	3.00	99	56
**4**	**2**	**6.00**	**76**	**94**

[a] Reaction conditions: According to the general procedure, **5a** (0.10 mmol), Λ-**RhInd** (2−8 mol%) and Na_2HPO_4 (0.11 mmol) were dissolved in MeOH/THF 4:1 (0.50 mL). After addition of H_2O (5.56 mmol) and **6a** (0.30–0.60 mmol), the reaction mixture was degassed and stirred for 24 h at r.t. in front of blue LEDs under an atmosphere of nitrogen. [b] Isolated yield. [c] Determined by chiral HPLC analysis.

Table S4. Survey of amount of base.

Entry	Base Equivalents	Yield [%][b]	*ee* [%][c]
1	1.10	76	94
2	1.50	78	94
**3**	**2.00**	**80**	**94**
4	2.50	27	92

[a] Reaction conditions: According to the general procedure, **5a** (0.10 mmol), Λ-**RhInd** (2 mol%) and Na_2HPO_4 (0.11 mmol) were dissolved in MeOH/THF 4:1 (0.50 mL). After addition of H_2O (5.56 mmol) and **6a** (0.60 mmol), the reaction mixture was degassed and stirred for 24 h at r.t. in front of blue LEDs under an atmosphere of nitrogen. [b] Isolated yield. [c] Determined by chiral HPLC analysis.

Table S5. Survey of imidazole auxiliary.[a]

Entry	2-acyl imidazole	Yield [%][b]	*ee* [%][c]
**1**	**5a**	**80**	**94**
2	**5b**	2	80
3	**5c**	52	94

[a] Reaction conditions: According to the general procedure, the indicated 2-acyl imidazole (**5a-c**) (0.10 mmol), Λ-**RhInd** (2 mol%) and Na_2HPO_4 (0.20 mmol) were dissolved in MeOH/THF 4:1 (0.50 mL). After addition of H_2O (5.56 mmol) and **6a** (0.60 mmol), the reaction mixture was degassed and stirred for 24 h at r.t. in front of blue LEDs under an atmosphere of nitrogen. [b] Isolated yield. [c] Determined by chiral HPLC analysis.

Table S6. Survey of different catalysts.[a]

Entry	Catalyst	Yield [%][b]	*ee* [%][c]
1	Λ-**RhInd**	80	94
2	Λ-**IrS**	23	94
3	Λ-**RhS**	83	90
4	Λ-**RhInd(Ph)**[d]	73	74
5	*rac* **RhInd(Ph-OMe)**[d]	43	–
6	*rac* **RhInd(Ph-CF_3)**[d]	13	–

[a] Reaction conditions: According to the general procedure, **5a** (0.10 mmol), the indicated catalyst (2 mol%) and Na_2HPO_4 (0.20 mmol) were dissolved in MeOH/THF 4:1 (0.50 mL). After addition of H_2O (5.56 mmol) and **6a** (0.60 mmol), the reaction mixture was degassed and stirred for 24 h at r.t. in front of blue LEDs under an atmosphere of nitrogen. [b] Isolated yield. [c] Determined by chiral HPLC analysis. [d] Modified catalysts are shown in Figure **S7**. The ligands were synthesized according to a reported procedure[14] starting from unmodified 2*H*-indazole ligand **1**. The catalysts were prepared analogously to the synthesis of **RhInd**.

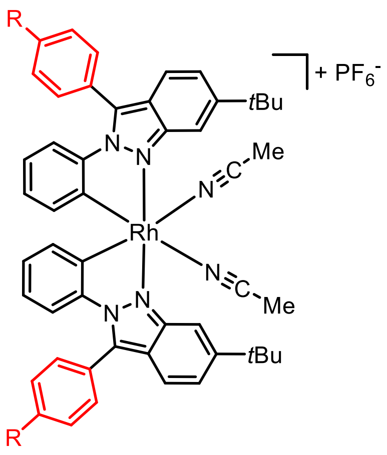

Figure S7. Structure of modified **RhInd**-catalysts.

3.3 Substrate Scope of α-Cyanomethylation of 2-Acyl Imidazoles

Standard Procedure for the Substrate Scope under Optimized Conditions

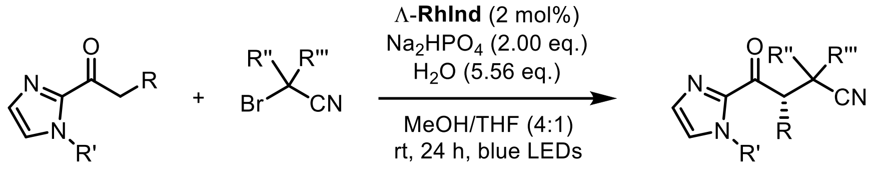

A 10 mL Schlenk tube was charged with 2-acyl imidazole **5a-j** (0.10 mmol), Λ-**RhInd** (2 mol%) and Na_2HPO_4 (0.20 mmol) and put under inert gas atmosphere. A mixture of MeOH/THF 4:1 (0.50 mL) and H_2O (5.56 eq.) were added and the resulting suspension was stirred for 5 min before α-cyano bromide **6a-f** was added and, subsequently, degassed via freeze-pump-thaw thoroughly for three cycles. The reaction mixture was then stirred for 24 h under inert gas atmosphere at r.t. in front of blue LEDs (24 W, 10 cm). Afterwards, the solvent was evaporated under vacuum and the precipitate was purified by column chromatography on silica gel (*n*-pentane/EtOAc 5:1 → 2:1) to afford pure products **7a-m**. Enantiomeric excess was determined by HPLC analysis on a chiral stationary phase. The absolute configuration of the products has been assigned based on a comparison with the crystal structure of an *o*-tolyl derivative (see Section 7, compound **S7**).

(*R*)-4-Oxo-3-phenyl-4-(1-phenyl-1*H*-imidazol-2-yl)butanenitrile (7a)

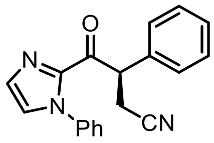

Following the general procedure, substrate **5a** (26.2 mg, 0.10 mmol) was used to obtain **7a** as a colorless solid (24.0 mg, 79.6 μmol, 80%). Enantiomeric excess was established by HPLC analysis using a Daicel Chiralcel AD-H column, *ee* = 94% (HPLC: 254 nm, *n*-hexane/isopropanol = 70:30, isocratic flow, flow rate 1.0 mL/min, 25 °C, t_r (major) = 7.5 min, t_r (minor) = 21.9 min).

TLC (*n*-pentane/EtOAc 2:1): R_f = 0.26. **^{1}H NMR** (300 MHz, $CDCl_3$): δ = 7.50-7.43 (m, 3H), 7.42-7.26 (m, 6H), 7.21-7.14 (m, 3H), 5.49 (t, J = 7.7 Hz, 1H), 3.00 (dd, J = 16.8, 7.7 Hz, 1H), 2.81 (dd, J = 16.8, 7.7 Hz, 1H) ppm. **^{13}C NMR** (75 MHz, $CDCl_3$): δ = 187.0, 141.8, 138.0, 136.2, 130.5, 129.3 (2C), 129.2 (2C), 129.2, 128.5 (2C), 128.4, 127.9, 125.8 (2C), 118.2, 49.6, 20.8 ppm. **IR** (neat): $\tilde{\nu}$ = 3135 (w), 2252 (w), 2168 (w), 1991 (w), 1959 (w), 1682 (s), 1593 (w), 1492 (m), 1448 (m), 1400 (s), 1301 (m), 1222 (w), 1146 (w), 1068 (m), 1009 (w), 971 (w), 932 (m), 902 (m), 819 (w), 755 (s), 693 (s), 573 (m), 409 (w) cm^{-1}. **$[\alpha]_D^{22}$** = –183.2° (*c* = 1.0, CH_2Cl_2). **HRMS (ESI)**: *m*/*z* calcd. for $C_{19}H_{16}N_3O$ [M + H$^+$]: 302.1288, found: 302.1286.

(*R*)-3-(4-Methoxyphenyl)-4-oxo-4-(1-phenyl-1*H*-imidazol-2-yl)butanenitrile (7d)

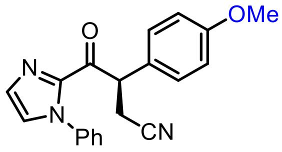

Following the general procedure, substrate **5d** (29.2 mg, 0.10 mmol) was used to obtain **7d** as a brown oil (20.2 mg, 61.0 μmol, 60%). Enantiomeric excess was established by HPLC analysis using a Daicel Chiralcel AD-H column, *ee* = 87% (HPLC: 254 nm, *n*-hexane/isopropanol = 70:30, isocratic flow, flow rate 1.0 mL/min, 25 °C, t_r (major) = 11.3 min, t_r (minor) = 18.1 min).

TLC (*n*-pentane/EtOAc 2:1): R_f = 0.36. **^{1}H NMR** (300 MHz, CD_2Cl_2): δ = 7.50-7.44 (m, 3H), 7.34-7.15 (m, 6H), 6.89 (d, *J* = 8.6 Hz, 2H), 5.40 (t, *J* = 7.7 Hz, 1H), 3.78 (s, 3H), 2.92 (dd, *J* = 16.1, 7.6 Hz, 1H), 2.79 (dd, *J* = 16.7, 7.9 Hz, 1H) ppm. **^{13}C NMR** (75 MHz, $CDCl_3$): δ = 187.2, 159.6, 141.8, 138.1, 130.4, 129.6 (2C), 129.2 (2C), 129.1, 128.1, 127.8, 125.8 (2C), 118.3, 114.7 (2C), 55.4, 48.8, 20.8 ppm. **IR** (neat): $\tilde{\nu}$ = 3129 (w), 2928 (w), 2840 (w), 2248 (w), 1682 (s), 1604 (m), 1505 (s), 1447 (m), 1399 (s), 1304 (m), 1249 (s), 1179 (m), 1150 (w), 1110 (w), 1068 (w), 1029 (m), 969 (w), 937 (w), 905 (m), 833 (m), 798 (s), 763 (s), 691 (s), 649 (w), 597 (w), 538 (m), 453 (w) cm^{-1}. **$[\alpha]_D^{22}$** = –194.4° (*c* = 1.0, CH_2Cl_2). **HRMS (ESI)**: *m*/*z* calcd. for $C_{20}H_{18}N_3O_2$ [M + H^+]: 332.1394, found: 332.1393.

(*R*)-3-(4-Chlorophenyl)-4-oxo-4-(1-phenyl-1*H*-imidazol-2-yl)butanenitrile (7e)

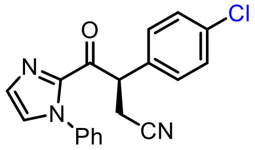

Following the general procedure, substrate **5e** (29.7 mg, 0.10 mmol) was used to obtain **7e** as a yellow solid (23.0 mg, 68.4 μmol, 68%). Enantiomeric excess was established by HPLC analysis using a Daicel Chiralcel AD-H column, *ee* = 85% (HPLC: 254 nm, *n*-hexane/isopropanol = 70:30, isocratic flow, flow rate 1.0 mL/min, 25 °C, t_r (major) = 8.1 min, t_r (minor) = 19.6 min).

TLC (*n*-pentane/EtOAc 2:1): R_f = 0.35. **^{1}H NMR** (300 MHz, $CDCl_3$): δ = 7.51-7.43 (m, 3H), 7.37-7.29 (m, 4H), 7.28-7.26 (m, 1H), 7.22-7.15 (m, 3H), 5.47 (t, *J* = 7.7 Hz, 1H), 2.96 (dd, *J* = 16.8, 7.5 Hz, 1H), 2.81 (dd, *J* = 16.8, 8.0 Hz, 1H) ppm. **^{13}C NMR** (75 MHz, $CDCl_3$): δ = 186.5, 141.6, 137.9, 134.7, 134.5, 130.6, 129.9 (2C), 129.5 (2C), 129.3 (3C), 128.2, 125.8 (2C), 117.9, 48.9, 20.6 ppm. **IR** (neat): $\tilde{\nu}$ = 3140 (w), 3060 (w), 2978 (w), 2922 (w), 2244 (w), 2171 (w), 2031 (w), 1679 (s), 1595 (w), 1490 (m), 1446 (m), 1400 (s), 1314 (m), 1229 (w), 1147 (w), 1089 (m), 1014 (m), 937 (m), 903 (m), 869 (w), 827 (m), 766 (s), 730 (m), 689 (s), 576 (w),

529 (s), 435 (m) cm^{-1}. **[α]$_D^{22}$** = –199.2° (c = 1.0, CH_2Cl_2). **HRMS (ESI)**: m/z calcd. for $C_{19}H_{14}Cl_1N_3ONa$ [M + Na$^+$]: 358.0718, found: 358.0719.

(*R*)-3-(4-Bromophenyl)-4-oxo-4-(1-phenyl-1*H*-imidazol-2-yl)butanenitrile (7f)

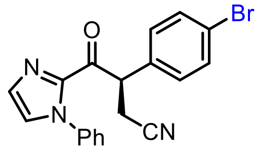

Following the general procedure, substrate **5f** (34.1 mg, 0.10 mmol) was used to obtain **7f** as a yellow solid (28.9 mg, 76.0 μmol, 76%). Enantiomeric excess was established by HPLC analysis using a Daicel Chiralcel AD-H column, *ee* = 84% (HPLC: 254 nm, *n*-hexane/isopropanol = 70:30, isocratic flow, flow rate 1.0 mL/min, 25 °C, t_r (major) = 8.5 min, t_r (minor) = 20.6 min).

TLC (*n*-pentane/EtOAc 2:1): R_f = 0.29. **^{1}H NMR** (300 MHz, $CDCl_3$): δ = 7.52-7.43 (m, 5H), 7.32-7.26 (m, 3H), 7.21-7.14 (m, 3H), 5.45 (t, J = 7.5 Hz, 1H), 2.96 (dd, J = 16.8, 7.4 Hz, 1H), 2.80 (dd, J = 16.8, 8.1 Hz, 1H) ppm. **^{13}C NMR** (75 MHz, $CDCl_3$): δ = 186.4, 141.5, 137.9, 135.2, 132.5 (2C), 130.7, 130.2 (2C), 129.3 (3C), 128.2, 125.8 (2C), 122.6, 117.9, 49.0, 20.6 ppm. **IR** (neat): $\tilde{\nu}$ = 3358 (w), 2922 (m), 2853 (m), 2245 (w), 1681 (m), 1595 (w), 1489 (m), 1448 (m), 1402 (s), 1314 (m), 1260 (m), 1145 (w), 1070 (m); 1014 (m), 938 (w), 904 (m), 867 (w), 796 (s), 764 (s), 688 (s), 575 (w), 529 (s), 398 (w) cm^{-1}. **[α]$_D^{22}$** = –107.2° (c = 1.0, CH_2Cl_2). **HRMS (ESI)**: m/z calcd. for $C_{19}H_{14}Br_1N_3ONa$ [M + Na$^+$]: 402.0212, found: 402.0215.

(*R*)-3-(Naphthalen-2-yl)-4-oxo-4-(1-phenyl-1*H*-imidazol-2-yl)butanenitrile (7g)

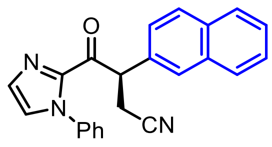

Following the general procedure, substrate **5g** (31.1 mg, 0.10 mmol) was used to obtain **7g** as an off-white solid (13.1 mg, 37.3 μmol, 37%). Enantiomeric excess was established by HPLC analysis using a Daicel Chiralcel AD-H column, *ee* = 76% (HPLC: 254 nm, *n*-hexane/isopropanol = 70:30, isocratic flow, flow rate 1.0 mL/min, 25 °C, t_r (major) = 10.2 min, t_r (minor) = 27.3 min).

TLC (*n*-pentane/EtOAc 2:1): R_f = 0.29. **^{1}H NMR** (300 MHz, CD_2Cl_2): δ = 7.89-7.81 (m, 4H), 7.53-7.45 (m, 6H), 7.24 (d, *J* = 0.9 Hz, 1H), 7.22-7.17 (m, 3H), 5.63 (t, 1H, *J* = 7.7 Hz, 1H), 3.07 (dd, *J* = 16.9, 7.7 Hz, 1H), 2.92 (dd, *J* = 16.8, 7.8 Hz, 1H) ppm. **^{13}C NMR** (75 MHz, $CDCl_3$): δ = 186.9, 141.9, 138.0, 133.7, 133.6, 133.1, 130.6, 129.2 (2C), 129.2, 128.2, 127.9, 127.9, 127.8, 126.6, 125.9, 125.9 (2C), 118.2, 49.7, 20.8 ppm. **IR** (neat): $\tilde{\nu}$ = 3137 (w), 3058 (w), 2928 (w), 2242 (w), 1968 (w), 1737 (w), 1680 (s), 1592 (m), 1493 (m), 1447 (m), 1402 (s), 1327 (w), 1296 (m), 1248 (w), 1187 (w), 1144 (m), 1066 (m), 1014 (w), 971 (m), 913 (w), 867 (w), 799 (s), 752 (s), 688 (s), 645 (m), 600 (w), 546 (m), 516 (w), 479 (m), 402 (w) cm^{-1}. **$[\alpha]_D^{22}$** = –288.4° (*c* = 1.0, CH_2Cl_2). **HRMS (ESI)**: *m*/*z* calcd. for $C_{23}H_{18}N_3O$ [M + H^+]: 352.1444, found: 352.1445.

(*R*)-4-Oxo-4-(1-phenyl-1*H*-imidazol-2-yl)-3-(thiophen-3-yl)butanenitrile (7i)

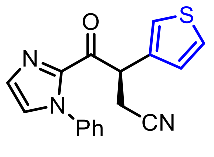

Following the general procedure, substrate **5i** (26.8 mg, 0.10 mmol) was used to obtain **7i** as an off-white solid (17.1 mg, 55.6 μmol, 56%). Enantiomeric excess was established by HPLC analysis using a Daicel Chiralcel AD-H column, *ee* = 78% (HPLC: 254 nm, *n*-hexane/isopropanol = 70:30, isocratic flow, flow rate 1.0 mL/min, 25 °C, t_r (major) = 8.8 min, t_r (minor) = 19.2 min).

TLC (*n*-pentane/EtOAc 2:1): R_f = 0.30. **^{1}H NMR** (300 MHz, $CDCl_3$): δ = 7.49-7.44 (m, 3H), 7.32-7.28 (m, 3H), 7.22-7.18 (m, 3H), 7.12-7.08 (m, 1H), 5.65 (t, *J* = 7.7 Hz, 1H), 2.98 (dd, *J* = 16.7, 7.7 Hz, 1H), 2.83 (dd, *J* = 16.7, 7.6 Hz, 1H) ppm. **^{13}C NMR** (75 MHz, $CDCl_3$): δ = 186.6, 141.7, 138.0, 136.2, 130.5, 129.3 (2C), 129.2, 128.1, 127.1, 126.8, 126.0, 125.8 (2C), 124.0, 118.1, 45.0, 20.5 ppm. **IR** (neat): $\tilde{\nu}$ = 3111 (w), 2922 (w), 2852 (w), 2247 (w), 1680 (s), 1594 (w), 1495 (m), 1445 (m), 1396 (s), 1308 (m), 1259 (w), 1198 (w), 1147 (m), 1069 (m), 1027 (w), 934 (w), 903 (m), 868 (w), 839 (w), 762 (s), 689 (s), 650 (m), 629 (m), 579 (w), 518 (m), 412 (w) cm^{-1}. **$[\alpha]_D^{22}$** = –82.0° (*c* = 1.0, CH_2Cl_2). **HRMS (ESI)**: *m*/*z* calcd. for $C_{17}H_{14}N_3OS$ [M + H^+]: 308.0852, found: 308.0851.

(*S*)-3-(1-Phenyl-1H-imidazole-2-carbonyl)pentanenitrile (7j)

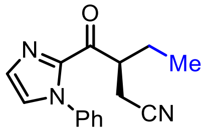

Following the general procedure, substrate **5j** (21.5 mg, 0.10 mmol) was used to obtain **7j** as a yellow oil (4.0 mg, 15.8 µmol, 16%). Enantiomeric excess was established by HPLC analysis using a Daicel Chiralcel AD-H column, *ee* = 50% (HPLC: 254 nm, *n*-hexane/isopropanol = 97:3, isocratic flow, flow rate 1.0 mL/min, 25 °C, t_r (major) = 28.7 min, t_r (minor) = 33.0 min).

TLC (*n*-pentane/EtOAc 2:1): R_f = 0.26. **^{1}H NMR** (300 MHz, $CDCl_3$): δ = 7.51-7.45 (m, 3H), 7.32-7.30 (m, 1H), 7.30-7.26 (m, 2H), 7.23 (d, *J* = 0.9 Hz, 1H), 4.15 (tt, *J* = 6.8 Hz, 1H), 2.67 (dd, *J* = 16.8, 7.3 Hz, 1H), 2.57 (dd, *J* = 16.8, 6.9 Hz, 1H), 2.05-1.90 (m, 1H), 1.90-1.74 (m, 1H), 0.95 (t, *J* = 7.5 Hz, 3H) ppm. **^{13}C NMR** (75 MHz, $CDCl_3$): δ = 190.6, 160.0, 142.1, 130.3, 129.3 (2C), 129.1, 127.9, 126.0 (2C), 64.6, 44.8, 25.5, 25.1, 10.9 ppm. **IR** (neat): $\tilde{\nu}$ = 3122 (w), 2964 (m), 2925 (m), 2855 (w), 2248 (w), 1725 (w), 1679 (s), 1594 (w), 1553 (w), 1495 (m), 1448 (m), 1404 (s), 1338 (m), 1303 (m), 1258 (w), 1214 (w), 1150 (w), 1100 (w), 1030 (m),

967 (w), 903 (m), 762 (s), 692 (s), 659 (w), 517 (w), 406 (w) cm^{-1}. **[α]$_D$^{22}** = –0.6° (*c* = 1.0, CH_2Cl_2). **HRMS (ESI)**: *m*/*z* calcd. for $C_{15}H_{15}N_3ONa$ [M + Na^+]: 276.1107, found: 276.1109.

(3*R*)-2-(Diethoxymethyl)-4-oxo-3-phenyl-4-(1-phenyl-1*H*-imidazol-2-yl)butanenitrile (7k)

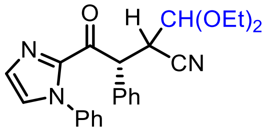

Following the general procedure, substrate **5a** (26.2 mg, 0.10 mmol) was used to obtain **7k**. The *d.r.* was determined by ^{1}H-NMR spectroscopy of the crude product (*d.r.* = 1.08:1). Diastereomer **7k-I** (13.1 mg, 32.5 μmol, 33%) was obtained as a yellow solid and diastereomer **7k-II** (12.4 mg, 30.7 μmol, 31%) was obtained as a yellow oil. Enantiomeric excess was established by HPLC analysis using a Daicel Chiralcel AD-H column (HPLC: 254 nm, *n*-hexane/isopropanol = 97:3, isocratic flow, flow rate 1.0 mL/min, 25 °C, **7k-I**: *ee* = 94%, t_r (major) = 4.2 min, t_r (minor) = 6.2 min; **7k-II**: *ee* = 95%, t_r (major) = 5.2 min, t_r (minor) = 7.1 min).

7k-I: **TLC** (*n*-pentane/EtOAc 2:1): R_f = 0.30. **^{1}H NMR** (300 MHz, $CDCl_3$): δ = 7.51-7.39 (m, 5H), 7.38-7.26 (m, 4H), 7.24-7.18 (m, 2H), 7.14 (s, 1H), 5.58 (d, *J* = 10.3 Hz, 1H), 4.11 (d, *J* = 3.7 Hz, 1H), 3.81-3.69 (m, 1H), 3.60-3.47 (m, 2H), 3.47-3.31 (m, 2H), 1.24 (t, *J* = 7.0 Hz, 3H), 1.11 (t, *J* = 7.0 Hz, 3H) ppm. **^{13}C NMR** (75 MHz, $CDCl_3$): δ = 187.0, 142.0, 138.2, 134.8, 130.5, 129.2 (2C), 129.2 (2C), 129.1 (2C), 129.0, 128.3, 127.8, 125.8 (2C), 118.3, 99.7, 64.2, 63.7, 52.2, 40.0, 15.3, 15.2 ppm. **IR** (neat): $\tilde{\nu}$ = 3117 (w), 3064 (w), 2976 (w), 2888 (w), 2248 (w), 2148 (w), 2037 (w), 1962 (w), 1681 (s), 1596 (w), 1495 (m), 1448 (m), 1397 (s), 1345 (w), 1309 (m), 1248 (w), 1121 (m), 1060 (s), 963 (m), 911 (w), 828 (w), 761 (s), 695 (s), 601 (w), 538 (m), 417 (w) cm^{-1}. **[α]$_D$^{22}** = –148.0° (*c* = 1.0, CH_2Cl_2). **HRMS (ESI)**: *m*/*z* calcd. for $C_{24}H_{26}N_3O_3$ [M + H^+]: 404.1969, found: 404.1973.

7k-II: **TLC** (*n*-pentane/EtOAc 2:1): R_f = 0.24. **^{1}H NMR** (300 MHz, $CDCl_3$): δ = 7.47-7.41 (m, 5H), 7.38-7.32 (m, 2H), 7.32-7.26 (m, 2H), 7.18-7.11 (m, 3H), 5.53 (d, *J* = 9.8 Hz, 1H), 4.60 (d, *J* = 5.9 Hz, 1H), 3.89 (dd, *J* = 9.8, 5.9 Hz, 1H), 3.77-3.63 (m, 2H), 3.62-3.44 (m, 2H), 1.18 (t, *J* = 7.0 Hz, 3H), 1.07 (t, *J* = 7.0 Hz, 3H) ppm. **^{13}C NMR** (75 MHz, $CDCl_3$): δ = 186.6, 142.1, 138.2, 135.2, 130.3, 129.5 (2C), 129.2 (4C), 129.0, 128.4, 127.6, 125.8 (2C), 118.1, 100.9, 64.1, 63.7, 50.9, 40.4, 15.2, 15.0 ppm. **IR** (neat): $\tilde{\nu}$ = 3116 (w), 3064 (w), 2978 (w), 2890 (w), 2248 (w), 1684 (s), 1596 (w), 1495 (m), 1448 (m), 1399 (s), 1345 (m), 1306 (m), 1254 (w), 1219 (w), 1116 (s), 1058 (s), 967 (m), 912 (w), 803 (m), 761 (s), 694 (s), 659 (m), 608 (w), 541 (m), 499 (m), 432 (w) cm^{-1}. **$[\alpha]_D^{22}$** = –248.6° (*c* = 1.0, CH_2Cl_2). **HRMS (ESI)**: *m/z* calcd. for $C_{24}H_{25}N_3O_3Na$ [M + Na^+]: 426.1784, found: 426.1792.

(3*R*)-2-methyl-4-oxo-3-phenyl-4-(1-phenyl-1*H*-imidazol-2-yl)butanenitrile (7l)

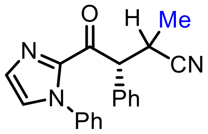

Following the general procedure, substrate **5a** (26.2 mg, 0.10 mmol) was used to obtain **7l**. The *d.r.* was determined by ^{1}H-NMR spectroscopy of the crude product (*d.r.* = 3.01:1). Diastereomer **7l-I** (17.0 mg, 53.9 μmol, 54%) was obtained as a yellow solid and diastereomer **7l-II** (5.3 mg, 16.8 μmol, 17%) was obtained as a yellow oil. Enantiomeric excess was established by HPLC analysis using a Daicel Chiralcel AD-H column (HPLC: 254 nm, *n*-hexane/isopropanol = 97:3, isocratic flow, flow rate 1.0 mL/min, 25 °C, **7l-I**: *ee* = 94%, t_r (major) = 5.9 min, t_r (minor) = 15.2 min; **7l-II**: *ee* = 83%, t_r (major) = 6.6 min, t_r (minor) = 9.3 min).

7l-I: **TLC** (*n*-pentane/EtOAc 2:1): R_f = 0.42. **^{1}H NMR** (300 MHz, $CDCl_3$): δ = 7.49-7.42 (m, 3H), 7.41-7.36 (m, 2H), 7.36-7.26 (m, 4H), 7.24-7.18 (m, 2H), 7.16 (d, *J* = 0.9 Hz, 1H), 5.33 (d, *J* = 11.0 Hz, 1H), 3.36-3.24 (m, 1H), 1.16 (d, *J* = 7.1 Hz, 3H) ppm. **^{13}C NMR** (75 MHz,

$CDCl_3$): δ = 187.6, 142.1, 138.1, 134.8, 130.4, 129.2 (2C), 129.2 (2C), 129.1 (2C), 128.3, 127.9, 125.8 (2C), 122.0, 77.4, 56.4, 27.6, 15.8 ppm. **IR** (neat): $\tilde{\nu}$ = 3137 (w), 3060 (w), 2994 (w), 2930 (w), 2242 (w), 2151 (w), 1994 (w), 1949 (w), 1680 (s), 1596 (w), 1494 (m), 1450 (m), 1403 (s), 1307 (m), 1259 (w), 1137 (w), 1068 (m), 1027 (m), 977 (w), 916 (m), 819 (m), 749 (s), 694 (s), 594 (w), 530 (m), 423 (w) cm^{-1}. **$[\alpha]_D^{22}$** = –240.8° (*c* = 1.0, CH_2Cl_2). **HRMS (ESI)**: *m*/*z* calcd. for $C_{20}H_{17}N_3ONa$ [M + Na^+]: 338.1264, found: 338.1265.

7l-II: **TLC** (*n*-pentane/EtOAc 2:1): R_f = 0.28. **^{1}H NMR** (300 MHz, $CDCl_3$): δ = 7.48-7.41 (m, 5H), 7.38-7.30 (m, 3H), 7.24-7.19 (m, 1H), 7.17-7.10 (m, 3H), 5.28 (d, *J* = 10.4 Hz, 1H), 3.56-3.45 (m, 1H), 1.37 (d, *J* = 6.9 Hz, 3H) ppm. **^{13}C NMR** (75 MHz, $CDCl_3$): δ = 187.0, 142.4, 138.0, 135.5, 130.4, 129.3 (2C), 129.2 (2C), 129.1 (2C), 129.1, 128.5, 128.3, 125.7, 121.5, 56.2, 29.2, 17.4, 15.8 ppm. **IR** (neat): $\tilde{\nu}$ = 3116 (w), 3063 (w), 2928 (w), 2242 (w), 1681 (s), 1596 (w), 1495 (m), 1449 (m), 1397 (s), 1306 (m), 1259 (w), 1207 (w), 1148 (w), 1073 (w), 1027 (m), 974 (w), 915 (m), 867 (w), 796 (m), 760 (s), 693 (s), 600 (w), 534 (m), 496 (w) cm^{-1}. **$[\alpha]_D^{22}$** = –91.6° (*c* = 1.0, CH_2Cl_2). **HRMS (ESI)**: *m*/*z* calcd. for $C_{20}H_{18}N_3O$ [M + H^+]: 316.1444, found: 316.1447.

(3*S*)-4-Oxo-2,3-diphenyl-4-(1-phenyl-1*H*-imidazol-2-yl)butanenitrile (7m)

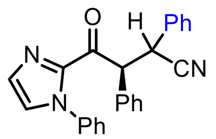

Following the general procedure, substrate **5a** (26.2 mg, 0.10 mmol) was used to obtain **7m**. The *d.r.* was determined by ^{1}H-NMR spectroscopy of the crude product (*d.r.* = 4.01:1). Diastereomer **7m-I** (18.0 mg, 47.7 μmol, 48%) was obtained as a colorless solid and diastereomer **7m-II** (3.1 mg, 8.2 μmol, 8%) was obtained as a colorless solid. Enantiomeric excess was established by HPLC analysis using a Daicel Chiralcel AD-H column. Only the analytical data of the major diastereomer **7m-I** will be listed below. This reaction was

performed with Δ-**RhInd**. Enantiomeric excess was established by HPLC analysis using a Daicel Chiralcel AD-H column (HPLC: 254 nm, *n*-hexane/isopropanol = 97:3, isocratic flow, flow rate 1.0 mL/min, 25 °C, **7m-I**: *ee* = 95%, t_r (minor) = 6.4 min, t_r (major) = 10.7 min.

7m-I: TLC (*n*-pentane/EtOAc 2:1): R_f = 0.21. **^{1}H NMR** (300 MHz, $CDCl_3$): δ = 7.51-7.46 (m, 3H), 7.28-7.23 (m, 3H), 7.21-7.14 (m, 9H), 7.11-7.05 (m, 2H), 5.61 (d, J = 11.2 Hz, 1H), 4.36 (d, J = 11.1 Hz, 1H) ppm. **^{13}C NMR** (75 MHz, $CDCl_3$): δ = 187.2, 142.1, 138.1, 134.3, 133.3, 130.5, 129.2 (2C), 129.2 (2C), 129.1, 128.9 (2C), 128.8 (2C), 128.6 (2C), 128.3, 128.1, 128.0, 125.9 (2C), 120.3, 58.3, 40.0 ppm. **IR** (neat): $\tilde{\nu}$ = 3141 (w), 3062 (w), 2946 (w), 2236 (w), 2159 (w), 2022 (w), 1949 (w) 1754 (w), 1679 (s), 1595 (w), 1493 (m), 1450 (m), 1401 (s), 1305 (m), 1248 (w), 1213 (w), 1181 (w), 1151 (w), 1108 (m), 1064 (w), 1027 (w), 987 (w), 941 (w), 912 (w), 871 (w), 837 (w), 796 (m), 756 (s), 693 (s), 623 (w), 582 (m), 535 (m), 479 (w), 432 (w) cm^{-1}. **$[\alpha]_D^{22}$** = 149.6° (*c* = 1.0, CH_2Cl_2). **HRMS (ESI)**: *m/z* calcd. for $C_{25}H_{19}N_3ONa$ [M + Na^+]: 400.1420, found: 400.1417.

4 Mechanistical Studies

4.1 Control Reactions

General Procedure for Control Experiments

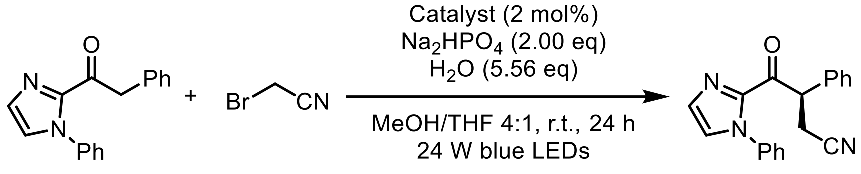

A 10 mL Schlenk tube was charged with 2-acyl imidazole **5a** (0.10 mmol), the indicated catalyst (2 mol%) and Na_2HPO_4 (0.20 mmol) and put under inert gas atmosphere. A mixture of MeOH/THF 4:1 (0.50 mL) and H_2O (5.56 eq.) were added and the resulting mixture was stirred for 5 min before bromoacetonitrile (**6a**, 0.60 mmol) was added and, subsequently, degassed via freeze-pump-thaw thoroughly for three cycles. The reaction mixture was then stirred for 24 h under inert gas atmosphere at r.t. in front of blue LEDs (24 W, 10 cm). Afterwards, the solvent was evaporated under vacuum and the precipitate was purified by column chromatography on silica gel (*n*-pentane/EtOAc 5:1 → 2:1) to afford pure product **7a**. Enantiomeric excess was determined by HPLC analysis on a chiral stationary phase.

Table S7. Control Experiments.

Entry	Variations from Standard Procedure	Catalyst	Yield [%][a]	*ee* [%][b]
1	without base	Λ-**RhInd**	–	–
2	without catalyst	–	–	–
3	under air	Λ-**RhInd**	–[c]	–
4	under air and without light	Λ-**RhInd**	20	86
5	with 1.0 equiv TEMPO	Λ-**RhInd**	25	14
6	with 6.0 equiv. TEMPO	Λ-**RhInd**	20	0
5	without H_2O	Λ-**RhInd**	77	80
6	CFL lamp	Λ-**RhInd**	26	94
7	50 °C	Λ-**IrS**	62	0
8	under air and without light	Λ-**RhS**	38	87

[a] Isolated yield. [b] Determined by chiral HPLC analysis. [c] Exclusive formation of α-keto-2-acyl imidazole **S6** as side product.

1-Phenyl-2-(1-phenyl-*1*H-imidazol-2-yl)ethane-1,2-dione (S6)

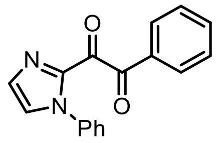

Analytical data of compound **S6** is in agreement with the literature.[15]

4.2 Racemization Control Experiments

For evaluation of racemization occurring during this reaction, the enantioenriched product **7a** (94% *ee*) was stirred with **RhInd**, **RhS**, and without catalyst in the presence of Na_2HPO_4.

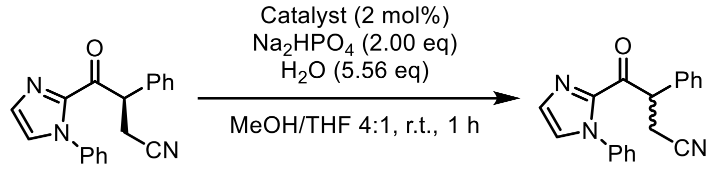

Table S 8. Control Experiments Concerning Racemization.[a]

Entry	Catalyst	*ee* [%][b]
1	Λ-**RhInd**	79.0
2	Λ-**RhS**	76.5
3	without catalyst	88.4

[a] Conditions: Enantioenriched product **7a** (1.00 eq.), the indicated catalyst (2 mol%) and Na_2HPO_4 (2.00 eq.) were dissolved in a mixture of MeOH/THF (4:1, 0.20 M) and H_2O (5.56 eq.) was added. The mixture was stirred for 1 h at r.t. and *ee* values were determined. [b] Determined by chiral HPLC analysis

Conclusion: As reported in Table S8, a significant racemization is occurring when enantioenriched product **7a** is stirred with base. However, in the presence of catalyst (entries 1 and 2) the racemization is faster than without catalyst (entry 3) explaining the observation that higher catalyst loadings lead to a drop in enantioselectivity for this reaction.

4.3 Synthesis and Characterization of RhInd-Enolate Complex II

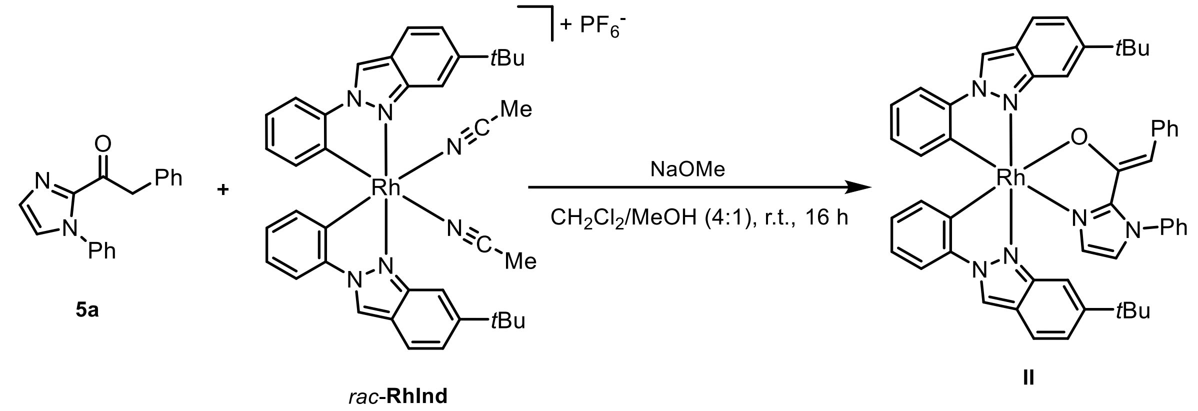

Following a procedure of MEGGERS *et al.*,[16] *rac*-**RhInd** (43.0 mg, 51.9 µmol, 1.00 eq.) and acyl imidazole **5a** (16.3 mg, 62.1 µmol, 1.20 eq.) were added to a flame dried Schlenk flask and dissolved in CH_2Cl_2/MeOH (4:1, 1 mL) under nitrogen atmosphere. NaOMe in MeOH (w = 25%, 18.0 µL, 77.8 µmol, 1.50 eq.) was added and the resulting mixture was stirred for 16 h at r.t.. Afterwards, the solvent was removed under vacuum and the precipitate was purified by column chromatography on silica gel (*n*-pentane/EtOAc 1:1 + 1% Et_3N). Catalyst-enolate-complex **II** (20.0 mg, 23.2 µmol, 45%) was obtained as a yellow solid.

TLC (*n*-pentane/EtOAc 1:1 + 1% NEt_3): R_f = 0.40. **^{1}H NMR** (300 MHz, CD_2Cl_2): δ = 8.67 (d, J = 0.7 Hz, 1H), 8.62 (d, J = 0.6 Hz, 1H), 8.13 (s, 1H), 7.71 (dd, J = 8.9, 0.5 Hz, 1H), 7.67 (dd, J = 9.0, 0.6 Hz, 1H), 7.57-7.36 (m, 8H), 7.33-7.26 (m, 4H), 7.08-6.91 (m, 5H), 6.84-6.67 (m, 5H), 6.21 (d, J = 1.2 Hz, 1H), 6.19 (d, J = 1.2 Hz, 1H), 4.77 (s, 1H), 1.24 (s, 9H), 1.05 (s, 9H) ppm. **HRMS (ESI)**: *m*/*z* calcd. for $C_{51}H_{48}N_6O$ [M + H$^+$]: 863.2939, found: 863.2949.

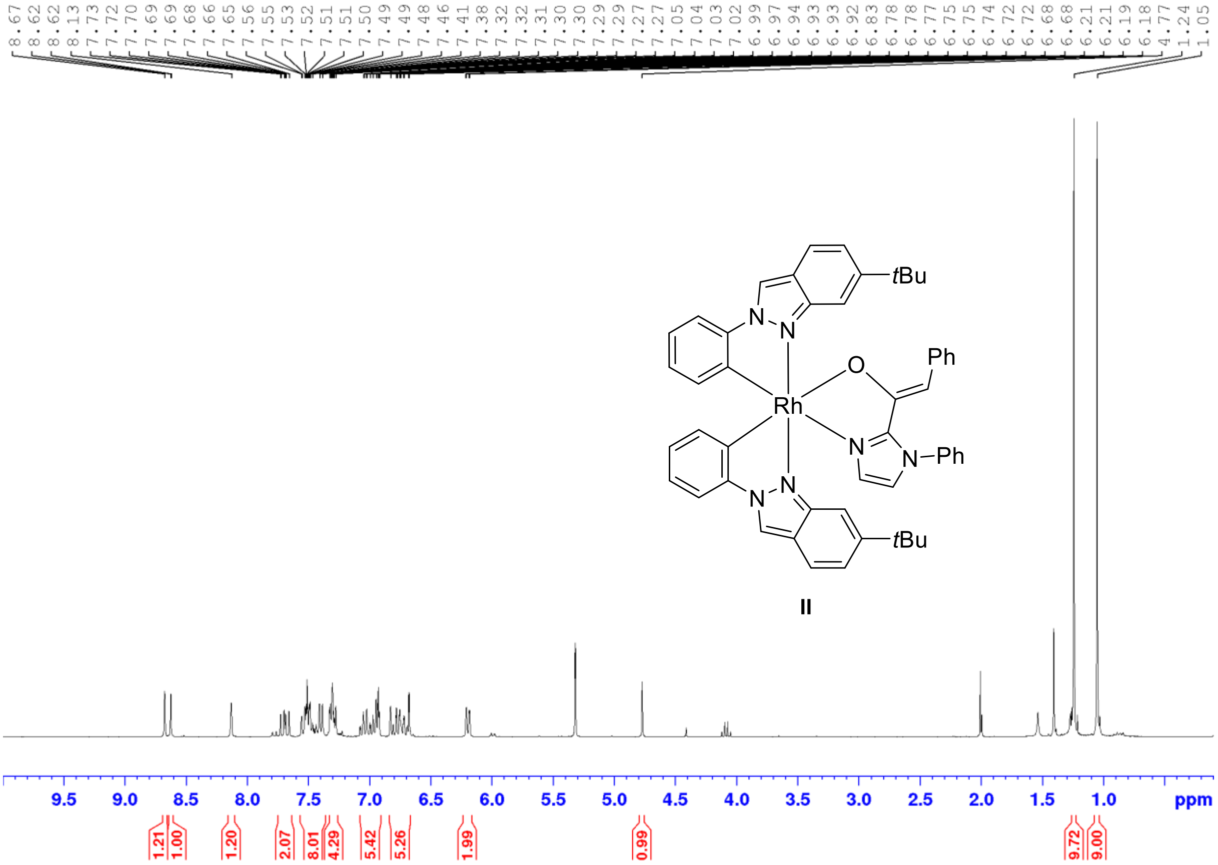

Figure S8. ^{1}H NMR (300 MHz) spectrum of **II** in CH_2Cl_2.

UV/VIS Absorbance of Substrate 5a, RhInd, and RhInd-Enolate II

As shown in Figure S9, **5a** has no absorption in visible light region. **RhInd** shows strong absorption in UV region but weak absorption at longer wavelengths at 400 nm and even imidazole-complex **I** exhibits no significant absorption in visible light region. Upon coordination and formation of **II**, the absorption of the resulting **RhInd** complex is significantly enhanced at 400-440 nm thus enabling an excitation by using blue LEDs.

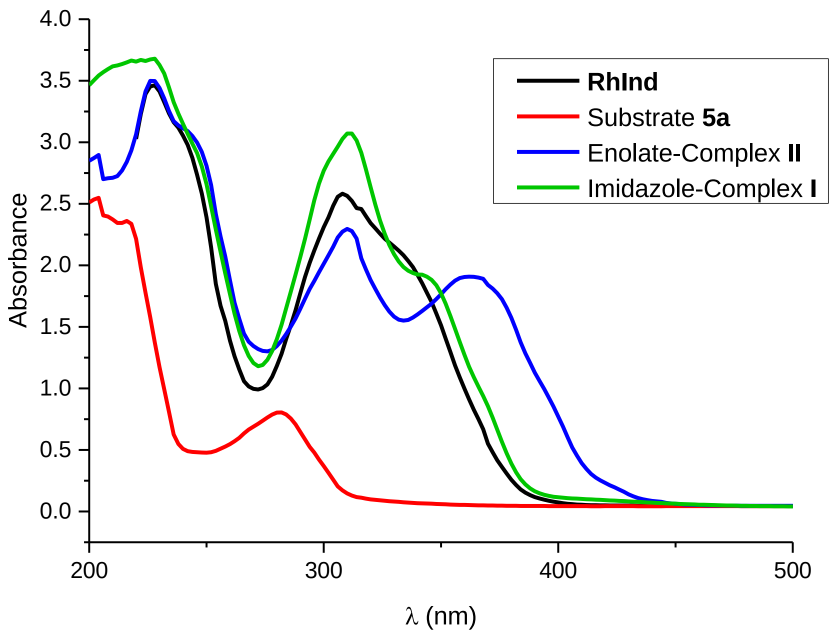

Figure S9. UV/VIS absorbance spectra of substrate **5a** (red), **RhInd** (black), imidazole-complex **I** (green) and enolate-complex **II** (blue) measured in CH_2Cl_2 (0.05 mM) in the absence of O_2.

4.4 Quantum Yield Determination

The quantum yield was measured following a procedure reported by MEGGERS *et al.*.[17] A 420 nm LED was employed as light source and a Powermeter was used as a detector. The measurement was done in a dark room with a 1.1 W red LED. The quantum yield was determined for the reaction **5a** + **6a** → **7a** following the below explained setup.

Step 1: The radiant power of light transmitted by the cuvette with a blank solution of MeOH/THF (4:1) was determined as P_{blank} = 24.8 mW.

Step 2: The reaction mixture of **5a** (105 mg, 0.40 mmol, 1.00 eq.), *rac*-**RhInd** (6.6 mg, 2 mol%), Na_2HPO_4 (114 mg, 0.80 mmol, 2.00 eq.) and H_2O (40.0 µL, 2.23 mmol, 5.56 eq.) dissolved in MeOH (1.60 mL) and THF (0.40 mL) was filled into a fluorescence cuvette with a stirring bar and **6a** (167 µL, 2.40 mmol,

6.00 eq.) was added in the dark. The reaction mixture was degassed with nitrogen (10 min) and the cuvette was placed into the setup and illuminated with a 420 nm LED. The transmitted radiant power $\underline{P^0_{sample} = 285\ \mu W}$ was noted at the beginning of the reaction.

Step 3: After illumination for 5 h (t = 18000 s), the transmitted radiant power $\underline{P^1_{sample} = 360\ \mu W}$ was noted again to calculate the average transmitted radiant power $\underline{P^{\varnothing}_{sample} = 323\ \mu W}$ and the amount of formed product **7a** was determined as 7.30 x 10^{-5} mol ($n_{product}$) by ^{1}H-NMR spectroscopy.

Step 4: The overall quantum yield can be calculated by the following formula:

$$\text{Quantum Yield} = \frac{N_{product}}{N_{photon}} = \frac{N_A \times n_{product}}{\frac{P_{absorbed} \times t}{\frac{h \times c}{\lambda}}} = \frac{h \times c \times N_A \times n_{product}}{\left(P_{blanc} - P^{\emptyset}_{sample}\right) \times t \times \lambda}$$

$$= \frac{6.626 \times 10^{-34}\text{Js} \times 2.998 \times 10^{8}\ \text{ms}^{-1} \times 6.022 \times 10^{23}\text{mol}^{-1} \times 7.30 \times 10^{-5}\text{mol}}{(25.42 - 0.32) \times 10^{-3}\text{Js}^{-1} \times 18000\ \text{s} \times 420 \times 10^{-9}\ \text{m}} = 0.046$$

5 NMR Spectra

5.1 NMR Spectra of Catalyst Synthesis

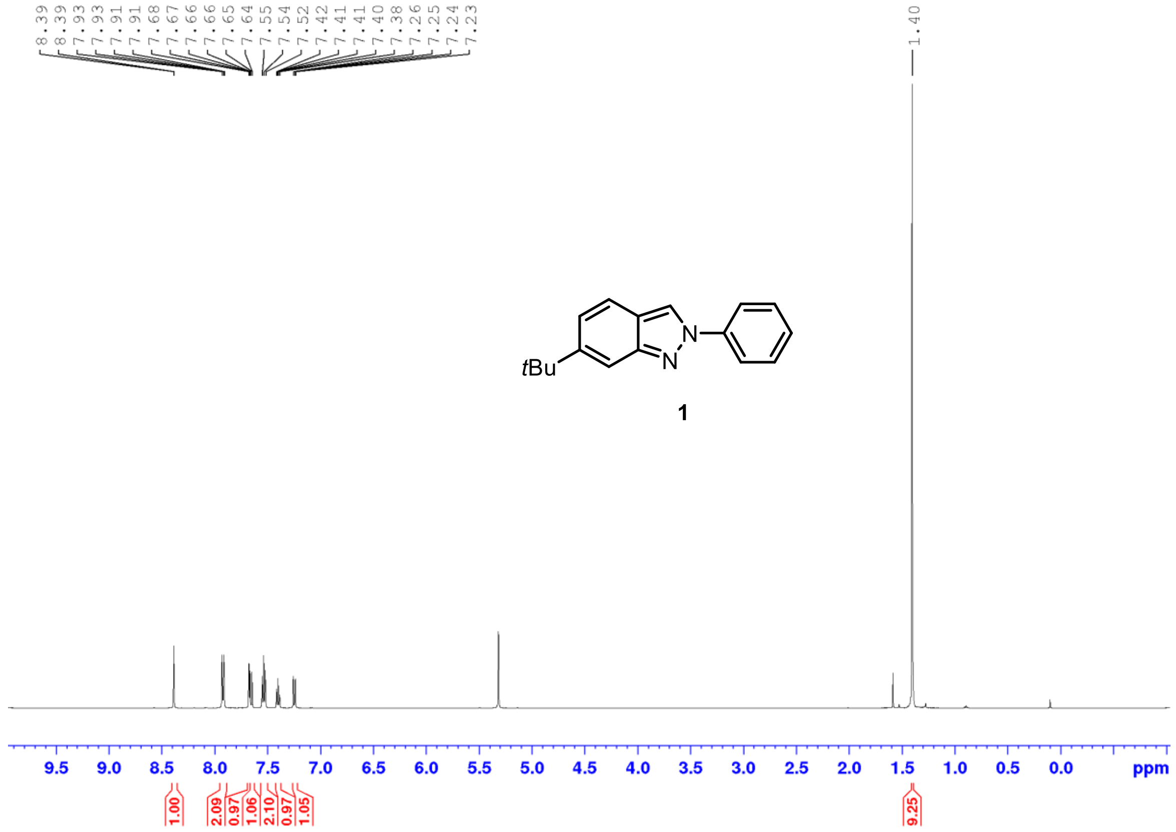

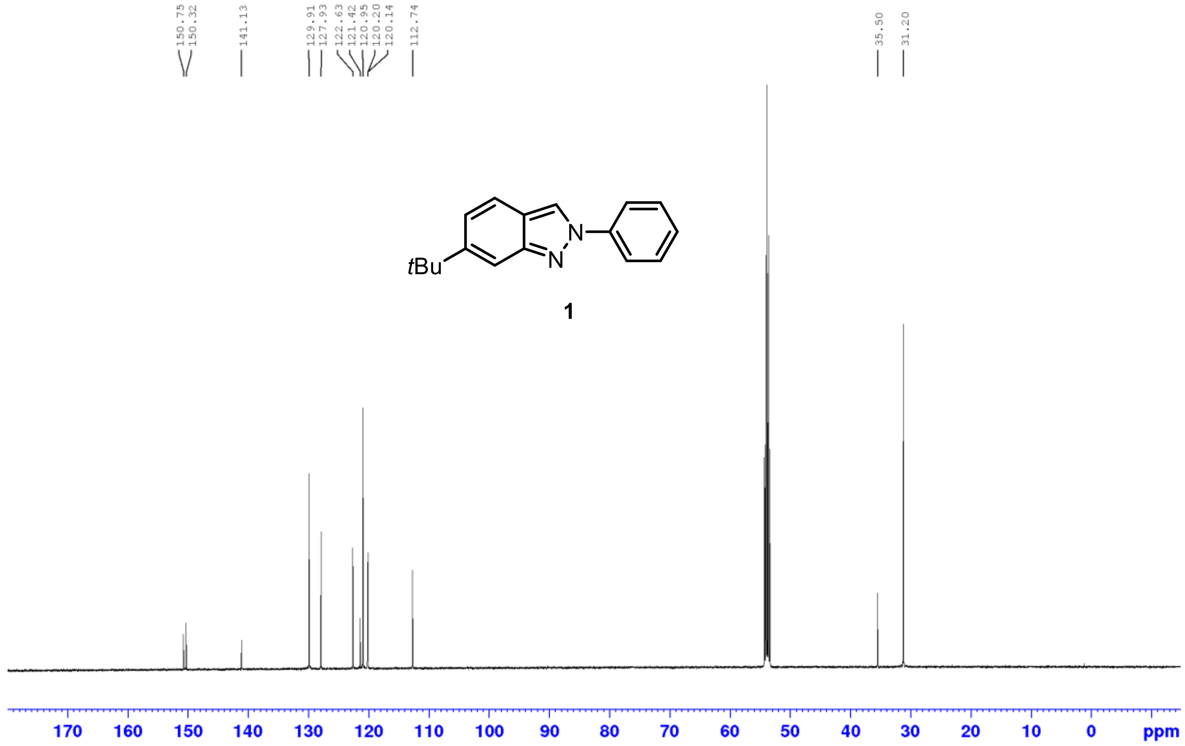

Figure S10. ^{1}H NMR (500 MHz) and ^{13}C NMR (127 MHz) spectrum of **1** in CD_2Cl_2.

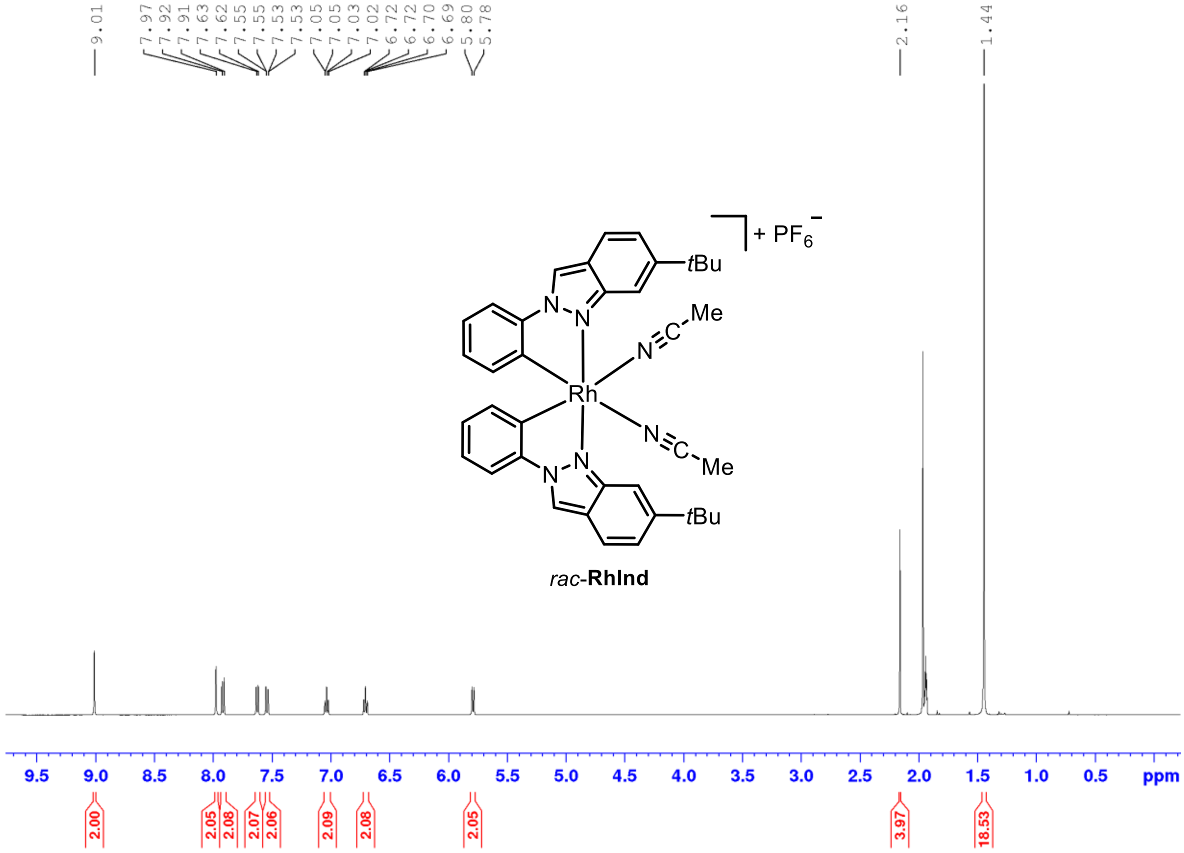
+ PF6
tBu
Me
Rh
Me
tBu
rac-RhInd
ppm

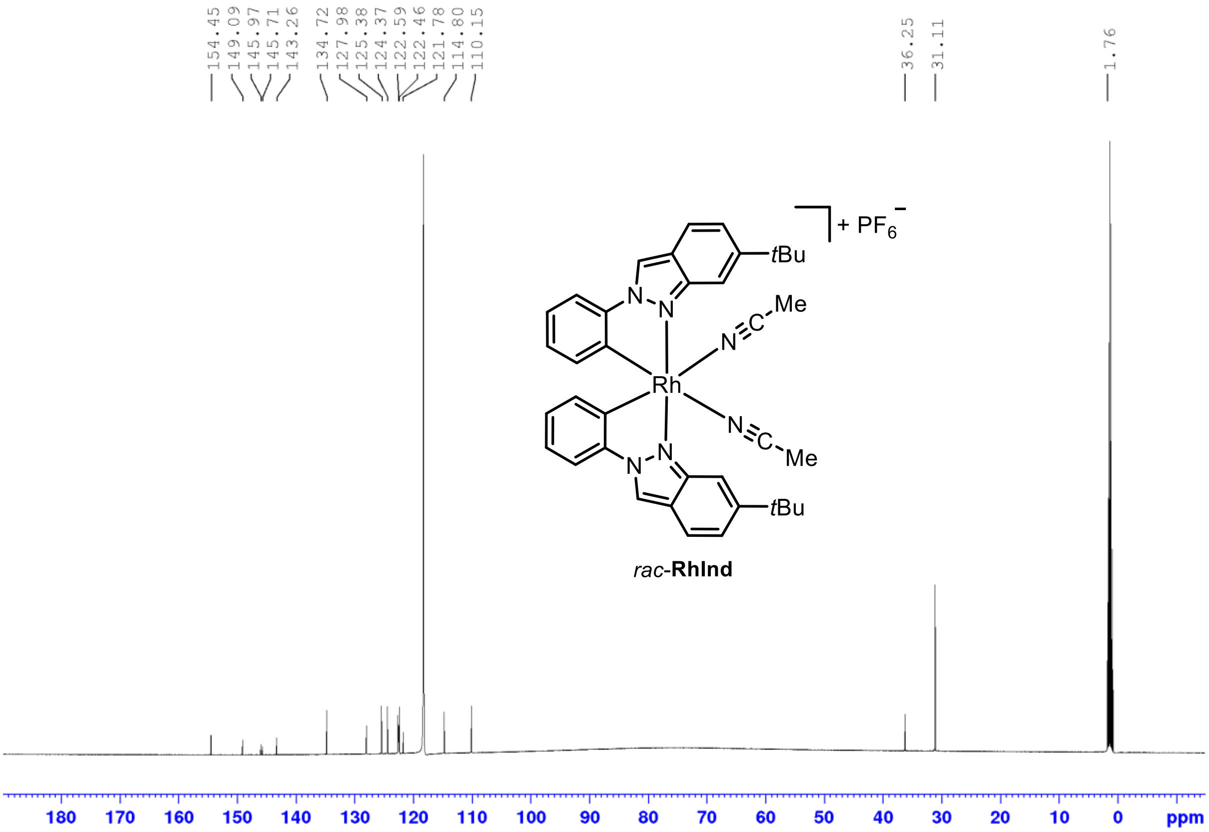
+ PF6
tBu
Me
Rh
Me
tBu
rac-RhInd
ppm

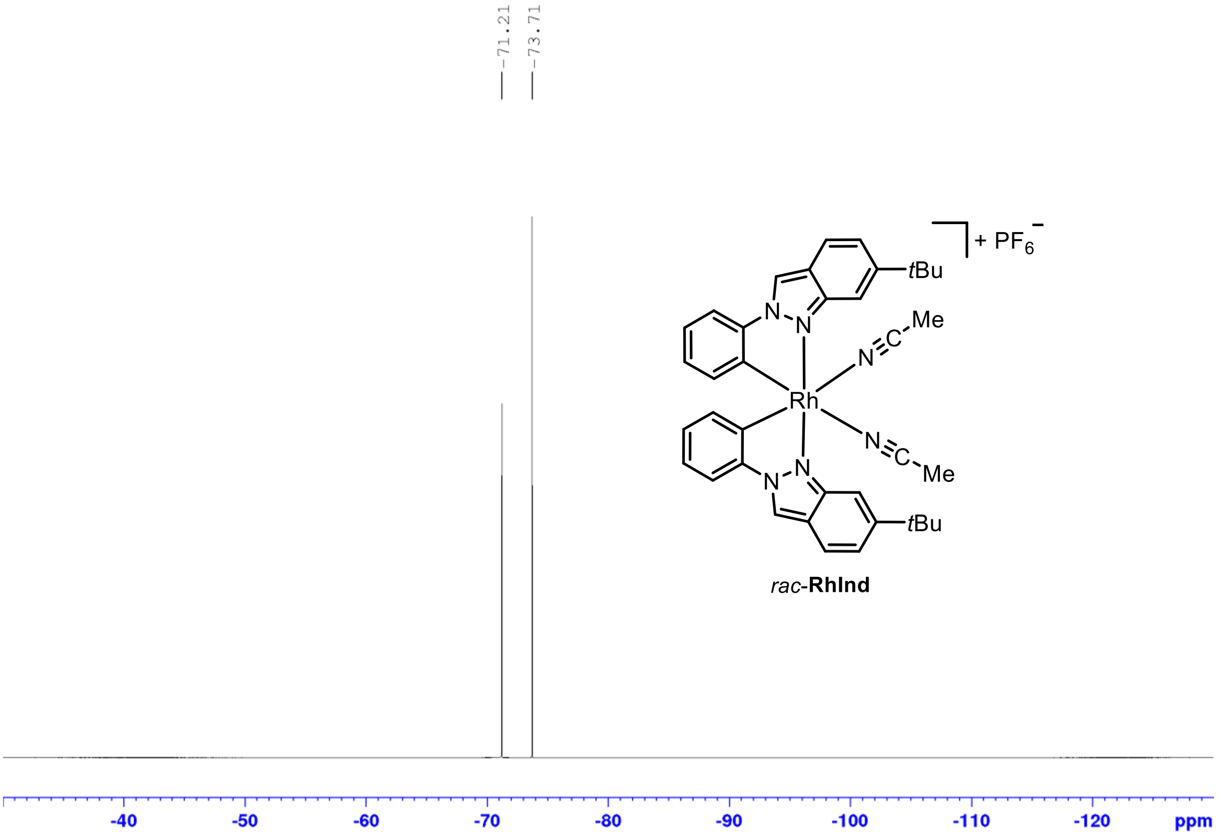

Figure S11. ^{1}H NMR (500 MHz), ^{13}C NMR (127 MHz), and ^{19}F NMR (283 MHz) spectra of *rac*-RhInd in CD_3CN.

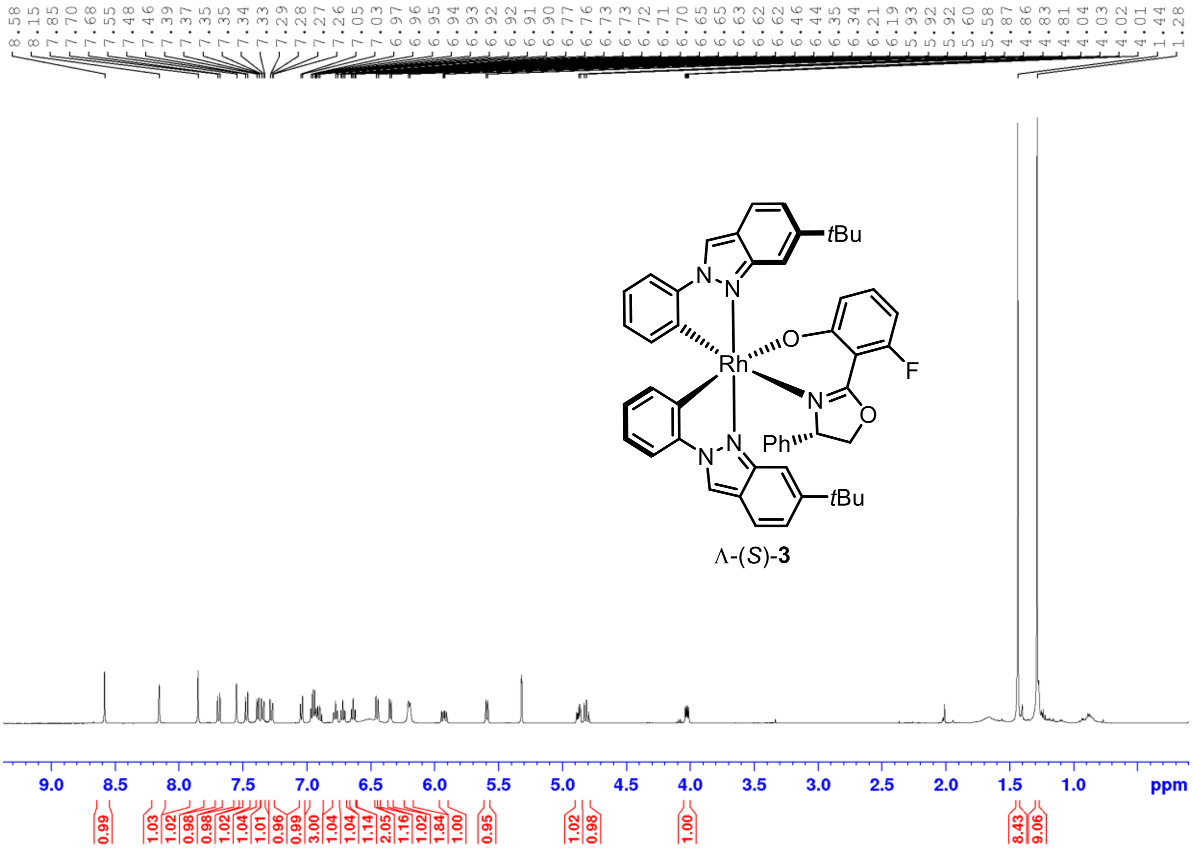

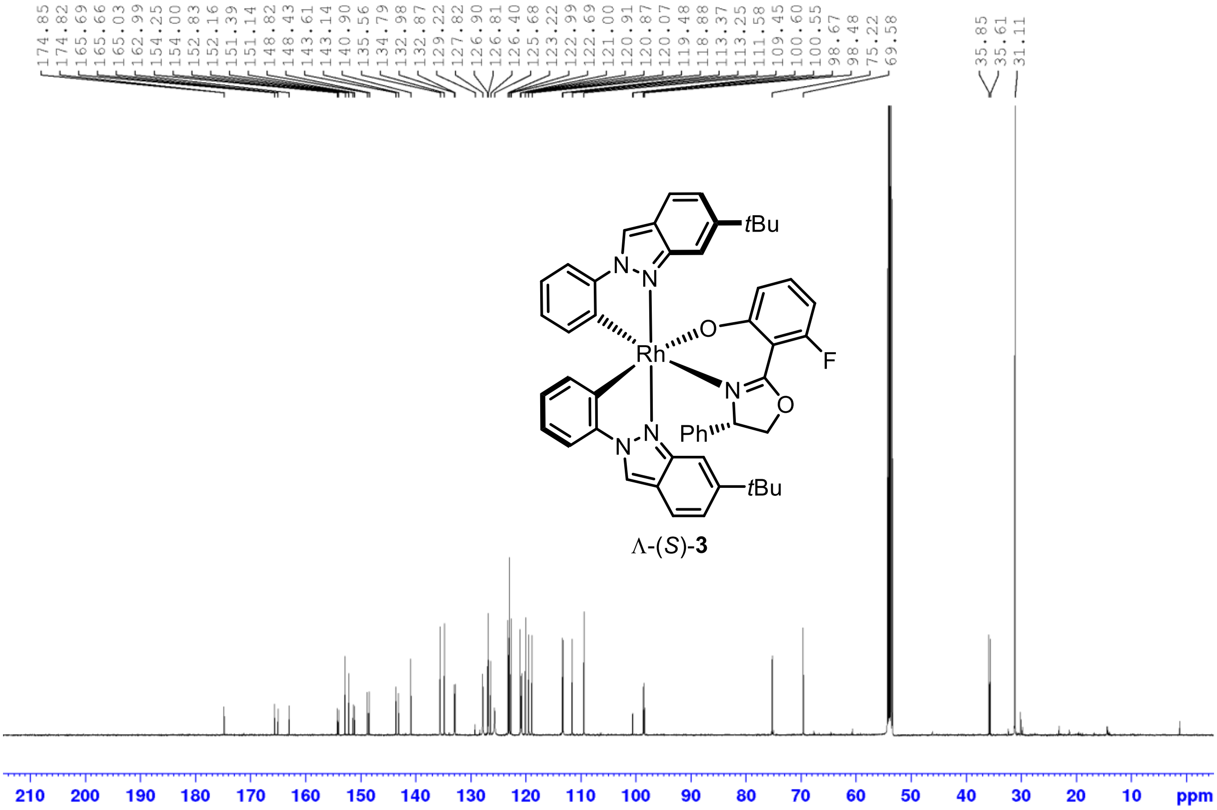

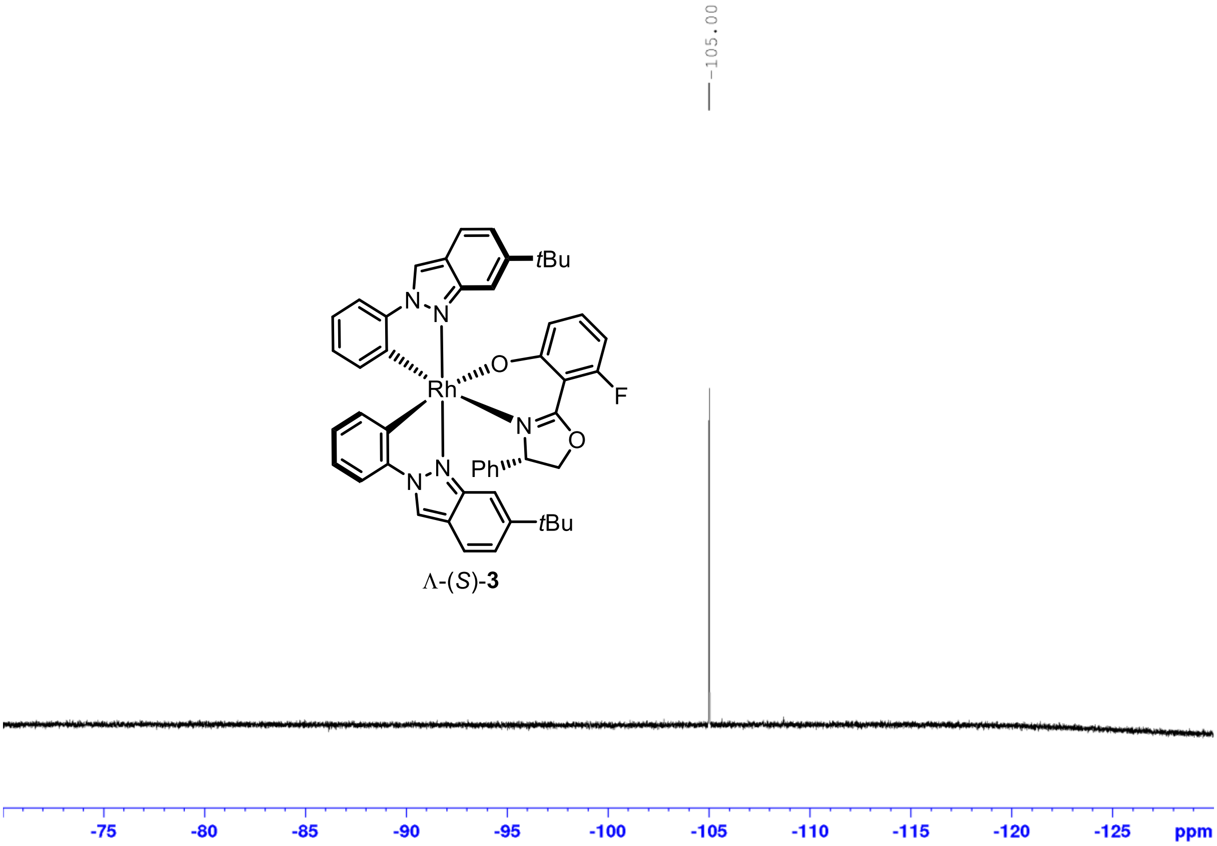

Figure S12. ^{1}H NMR (500 MHz), ^{13}C NMR (127 MHz), and ^{19}F NMR (235 MHz) spectra of Λ-(*S*)-**3** in CD_2Cl_2.

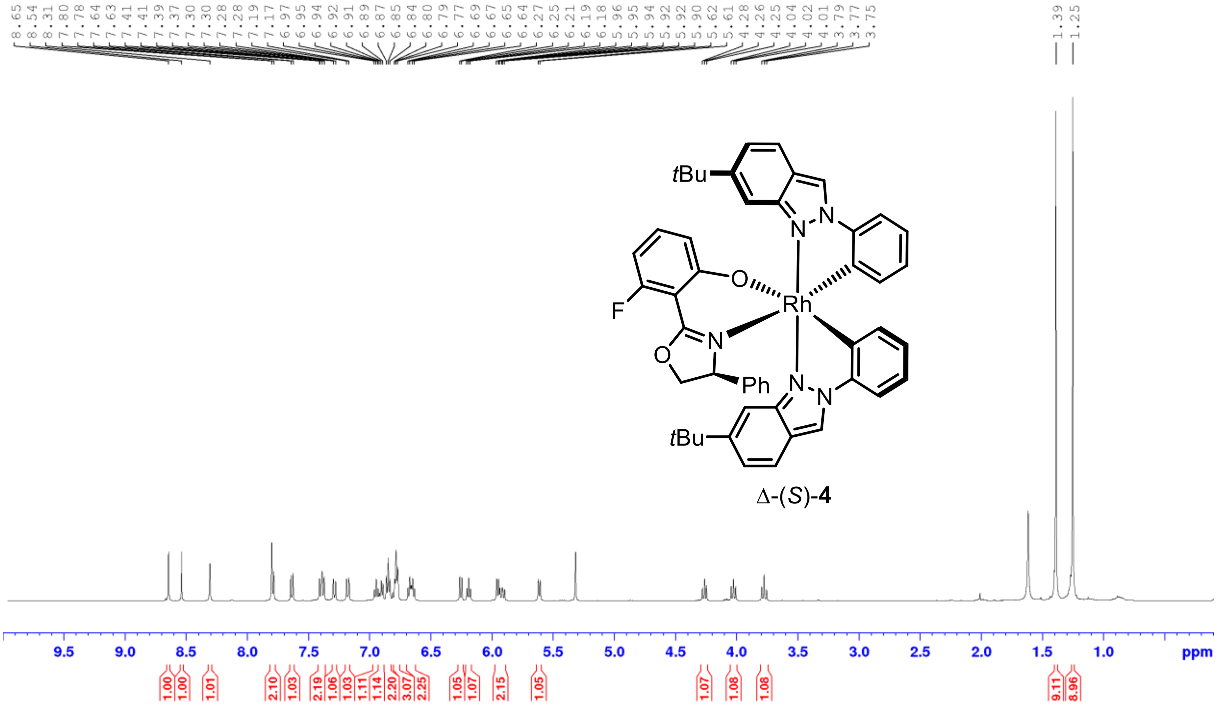
8.65 8.54 8.31 7.80 7.78 7.64 7.63 7.41 7.39 7.37 7.30 7.28 7.28 7.19 7.17 6.97 6.95 6.94 6.92 6.91 6.89 6.87 6.85 6.84 6.80 6.79 6.77 6.69 6.67 6.65 6.64 6.27 6.25 6.21 6.19 6.18 5.96 5.95 5.94 5.92 5.92 5.90 5.62 5.61 4.28 4.26 4.25 4.04 4.02 4.01 3.79 3.77 3.75
1.39 1.25
tBu
N
N
F
O
Rh
O
N
Ph
N
N
tBu
Δ-(S)-4
9.5 9.0 8.5 8.0 7.5 7.0 6.5 6.0 5.5 5.0 4.5 4.0 3.5 3.0 2.5 2.0 1.5 1.0 ppm
1.00 1.00 1.01 2.10 1.03 2.19 1.06 1.03 1.11 1.14 2.20 3.07 2.25 1.05 1.07 2.15 1.05 1.07 1.08 1.08 9.11 8.96

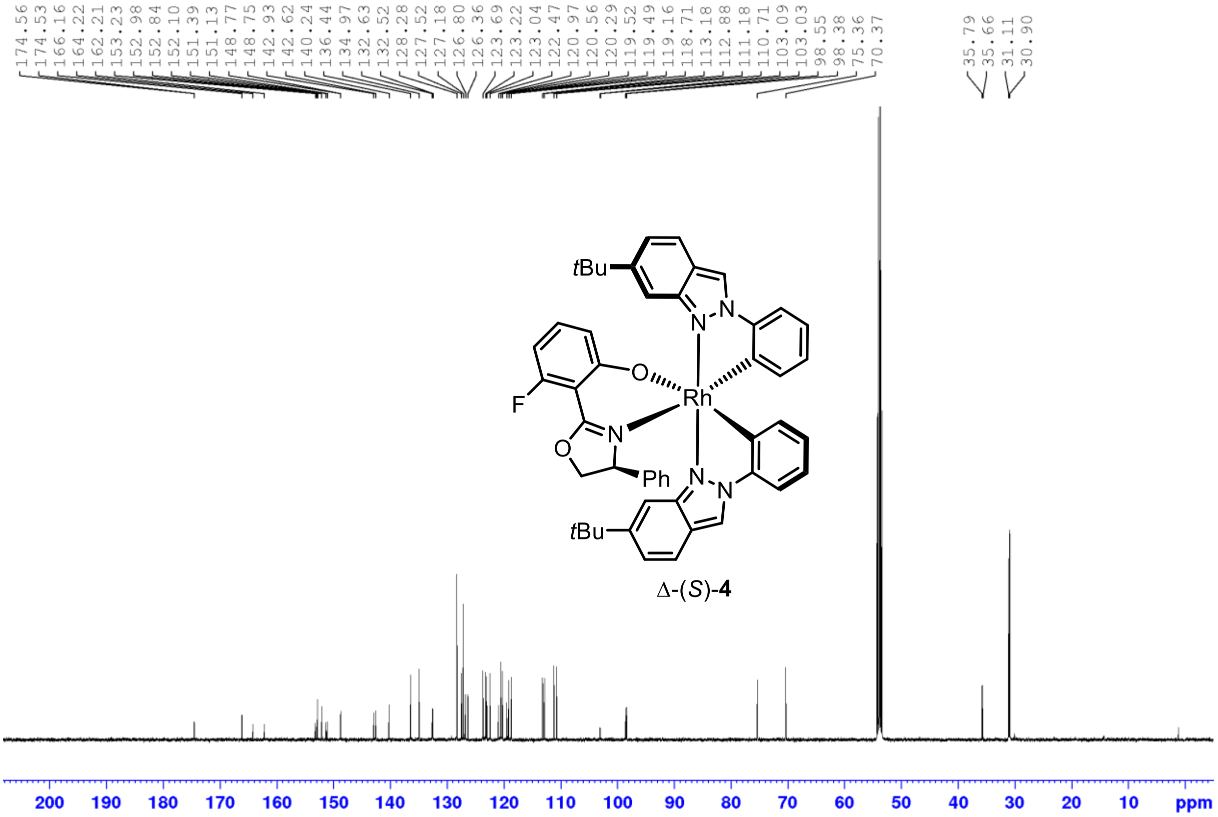
174.56 174.53 166.16 164.22 162.21 153.23 152.98 152.84 152.10 151.39 151.13 148.77 148.75 142.93 142.62 140.24 136.44 134.97 132.63 132.52 128.28 127.52 127.18 126.80 126.36 123.69 123.22 123.04 122.47 120.97 120.56 120.29 119.52 119.49 119.16 118.71 113.18 112.88 111.18 110.71 110.09 103.09 103.03 98.55 98.38 75.36 70.37
35.79 35.66 31.11 30.90
tBu
N
N
F
O
Rh
O
N
Ph
N
N
tBu
Δ-(S)-4
200 190 180 170 160 150 140 130 120 110 100 90 80 70 60 50 40 30 20 10 ppm

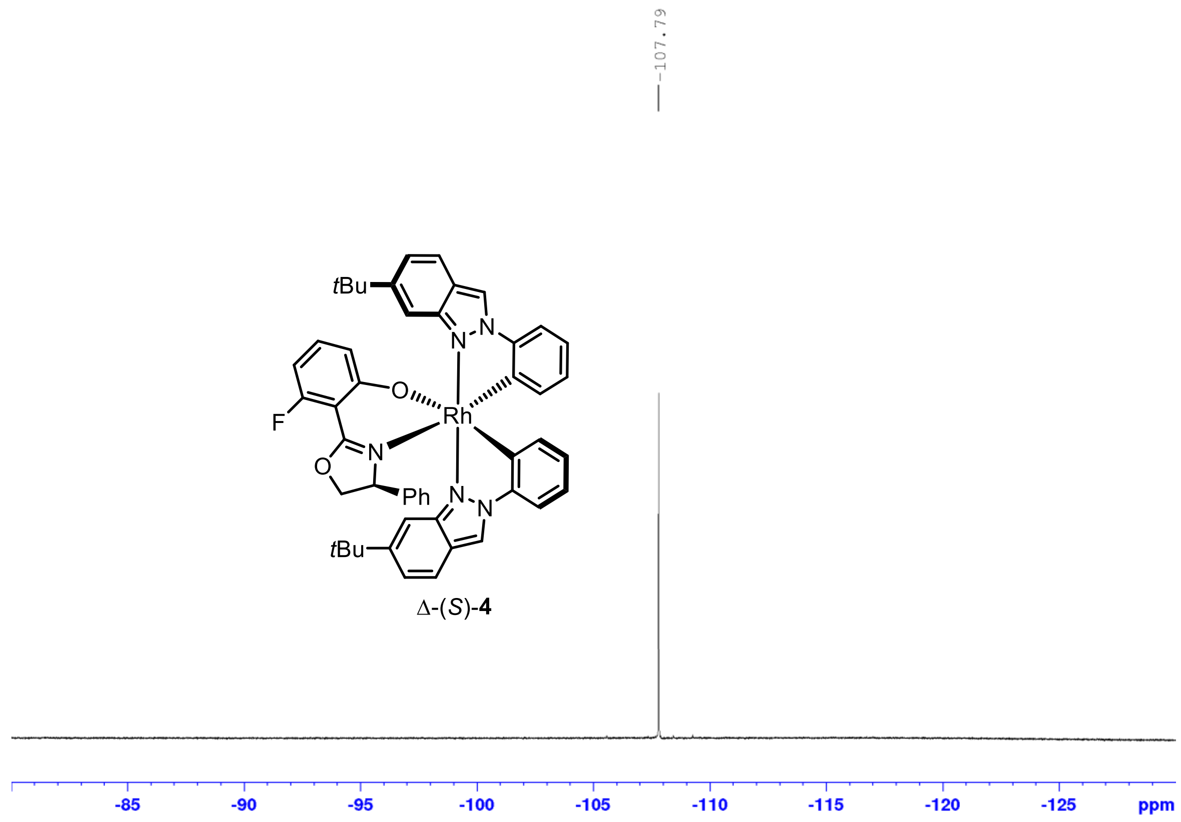

Figure S13. ^{1}H NMR (500 MHz), ^{13}C NMR (127 MHz), and ^{19}F NMR (235 MHz) spectra of Δ-(*S*)-**4** in CD_2Cl_2.

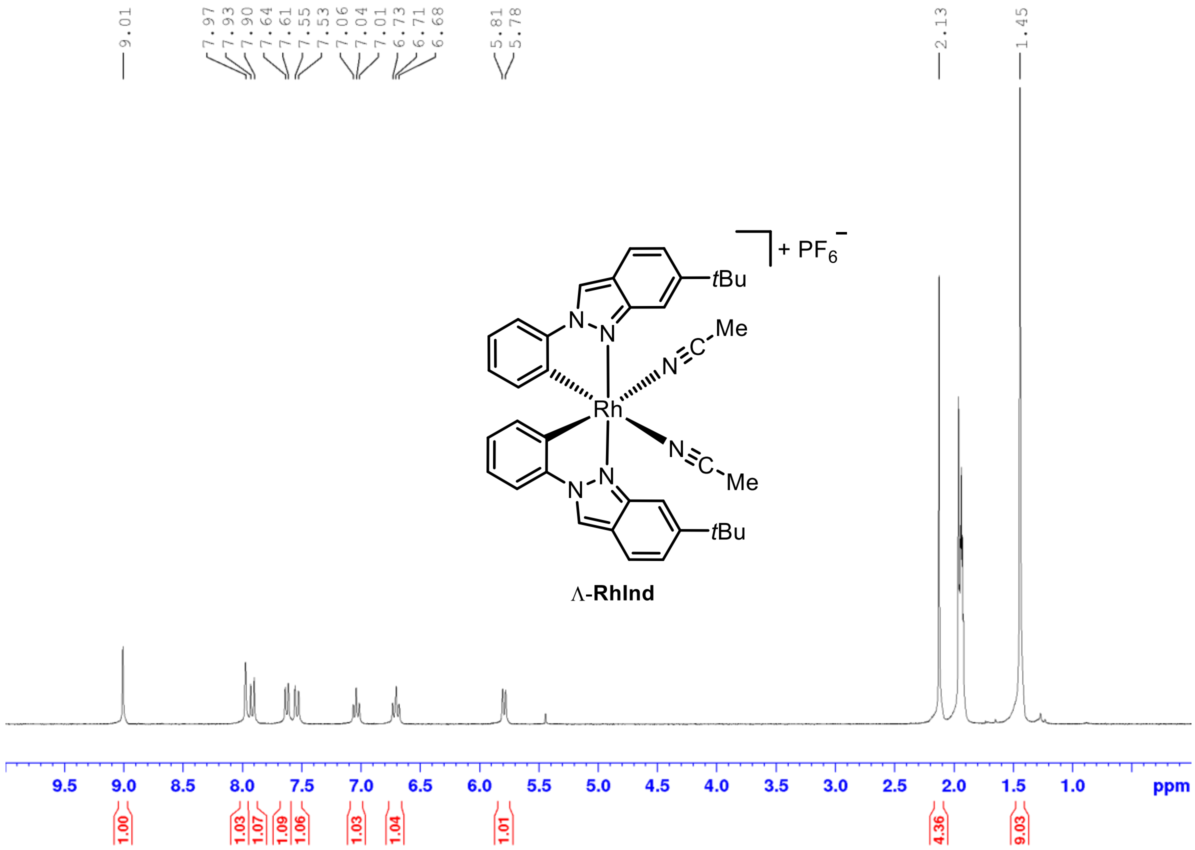

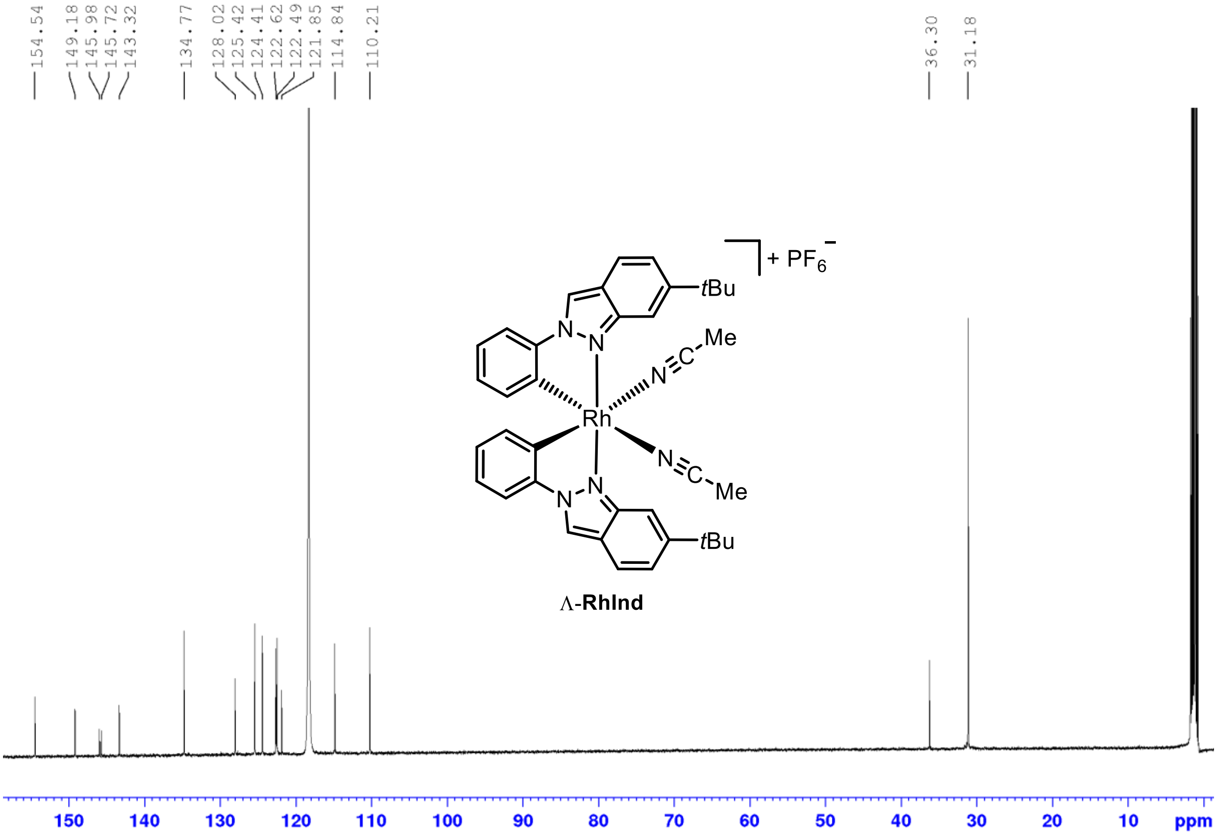

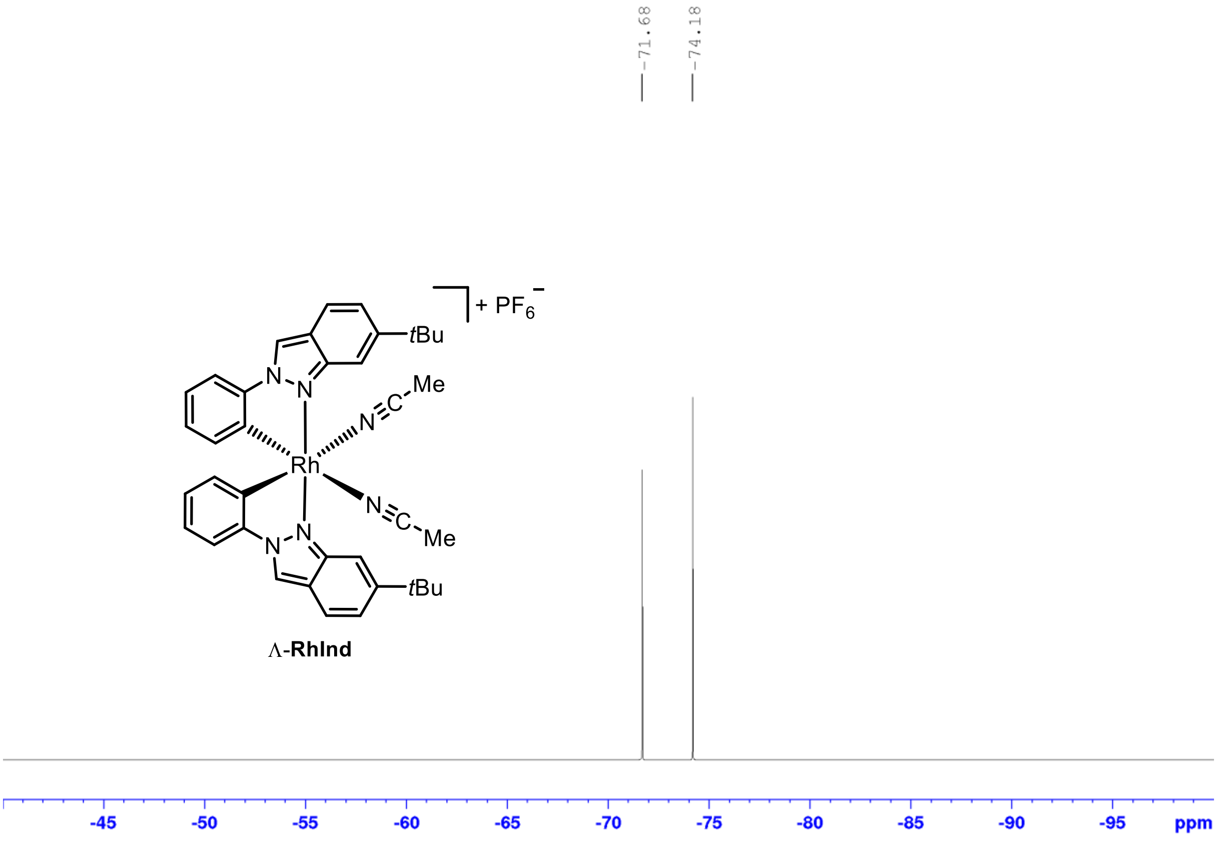

Figure S14. ^{1}H NMR (300 MHz), ^{13}C NMR (127 MHz), and ^{19}F NMR (283 MHz) spectra of **Λ-RhInd** in CD_3CN. The spectra of Δ-RhInd are identical and will not be shown.

5.2 NMR Spectra of Substrate Scope for α-Cyanomethylation

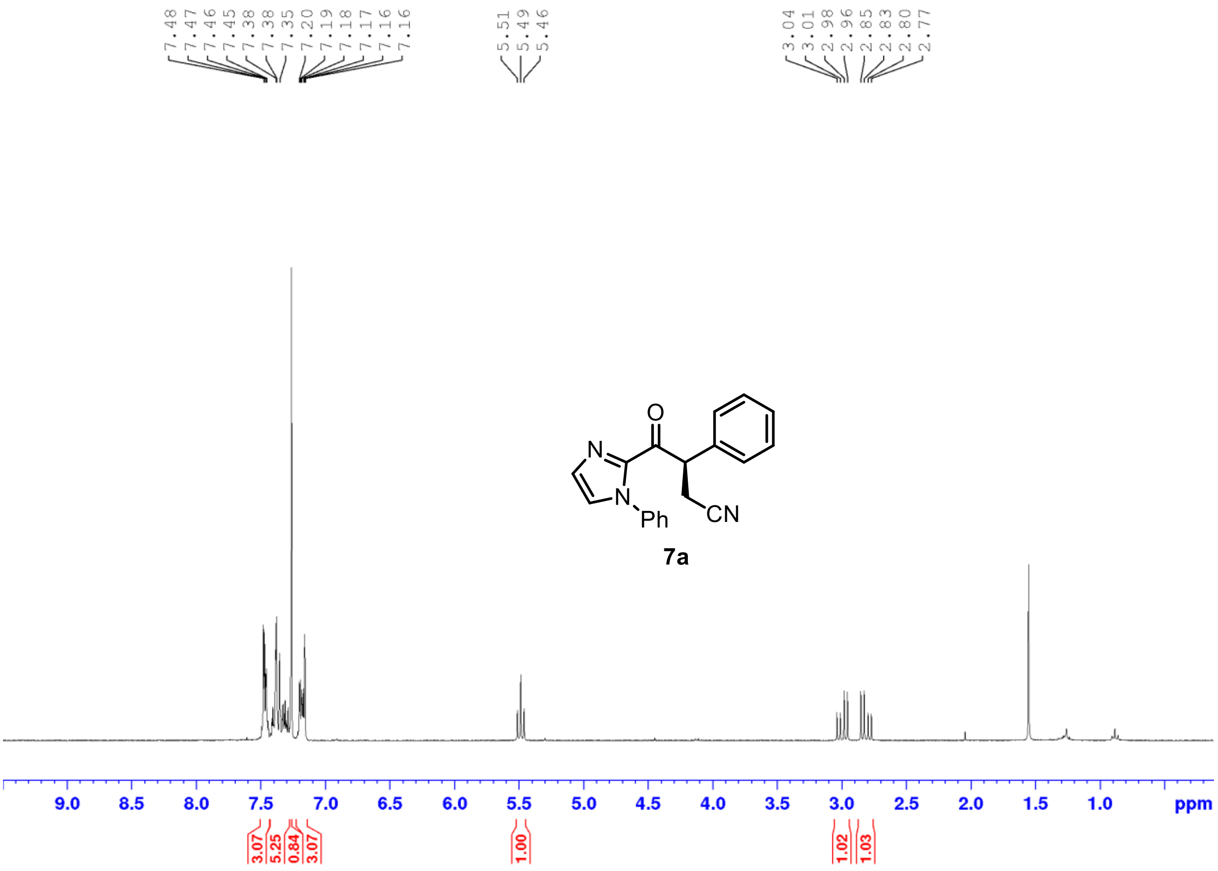

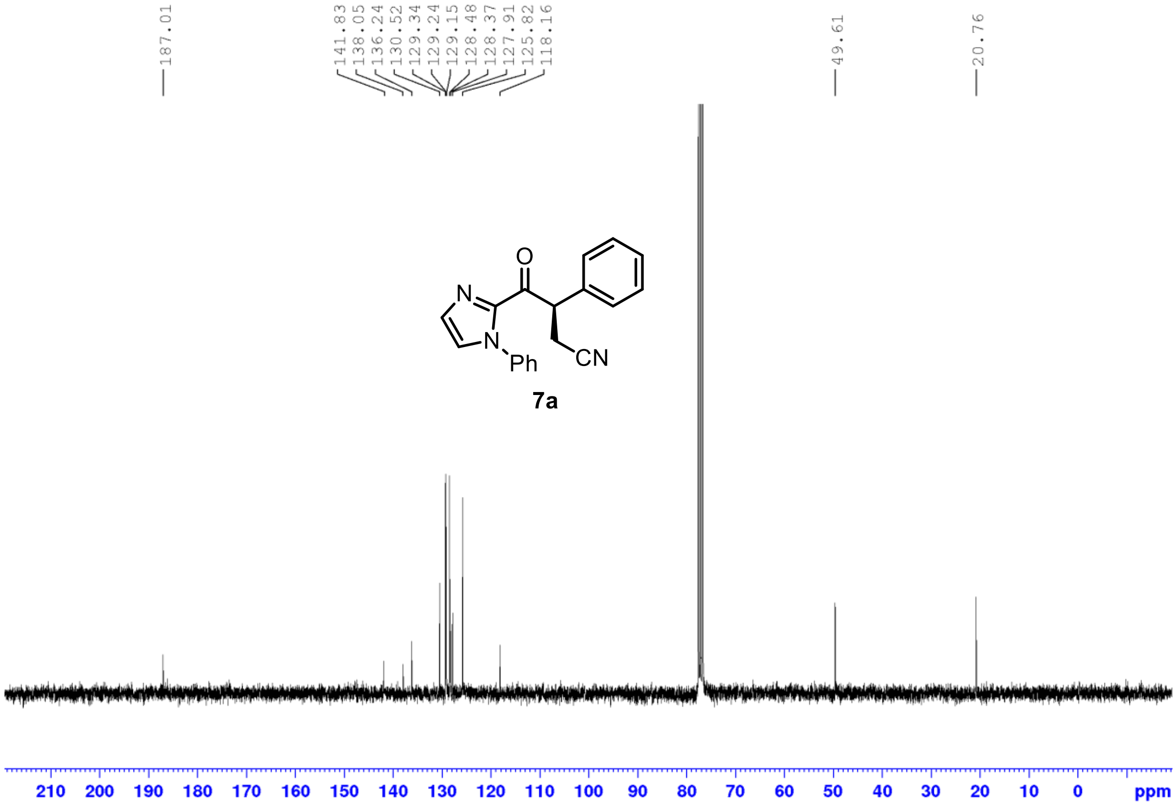

Figure S15. ^{1}H (300 MHz) and ^{13}C (75 MHz) spectra of **7a** in $CDCl_3$.

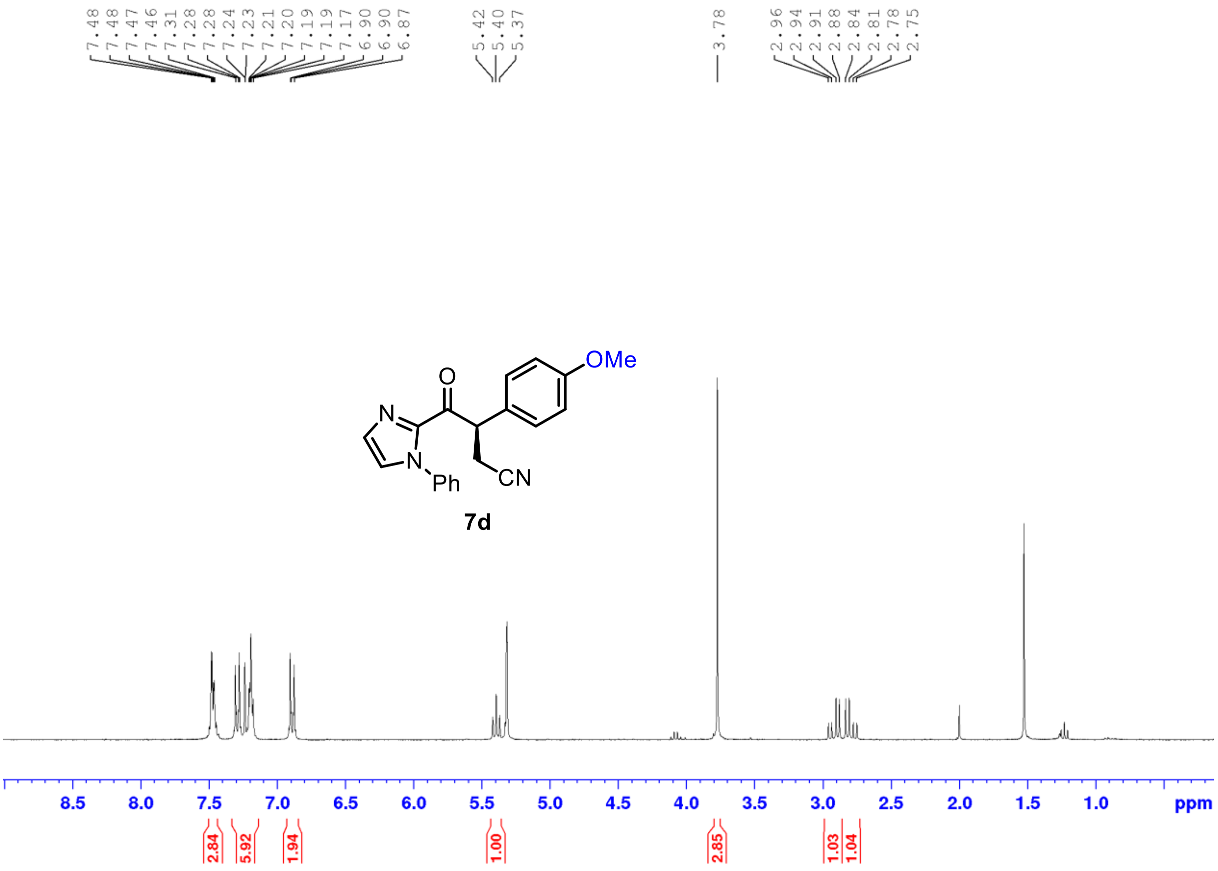

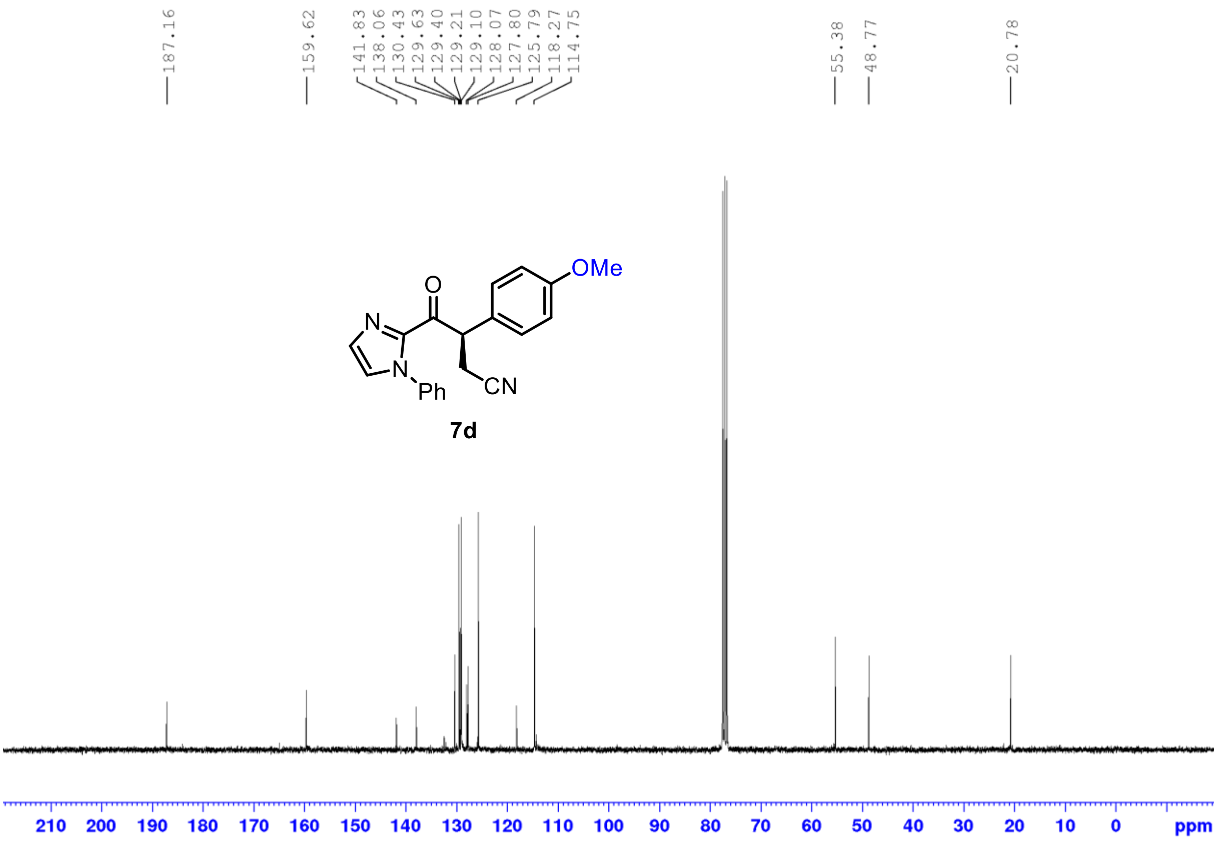

Figure S16. ^{1}H (300 MHz, CD_2Cl_2) and ^{13}C (75 MHz, $CDCl_3$) spectra of **7d**.

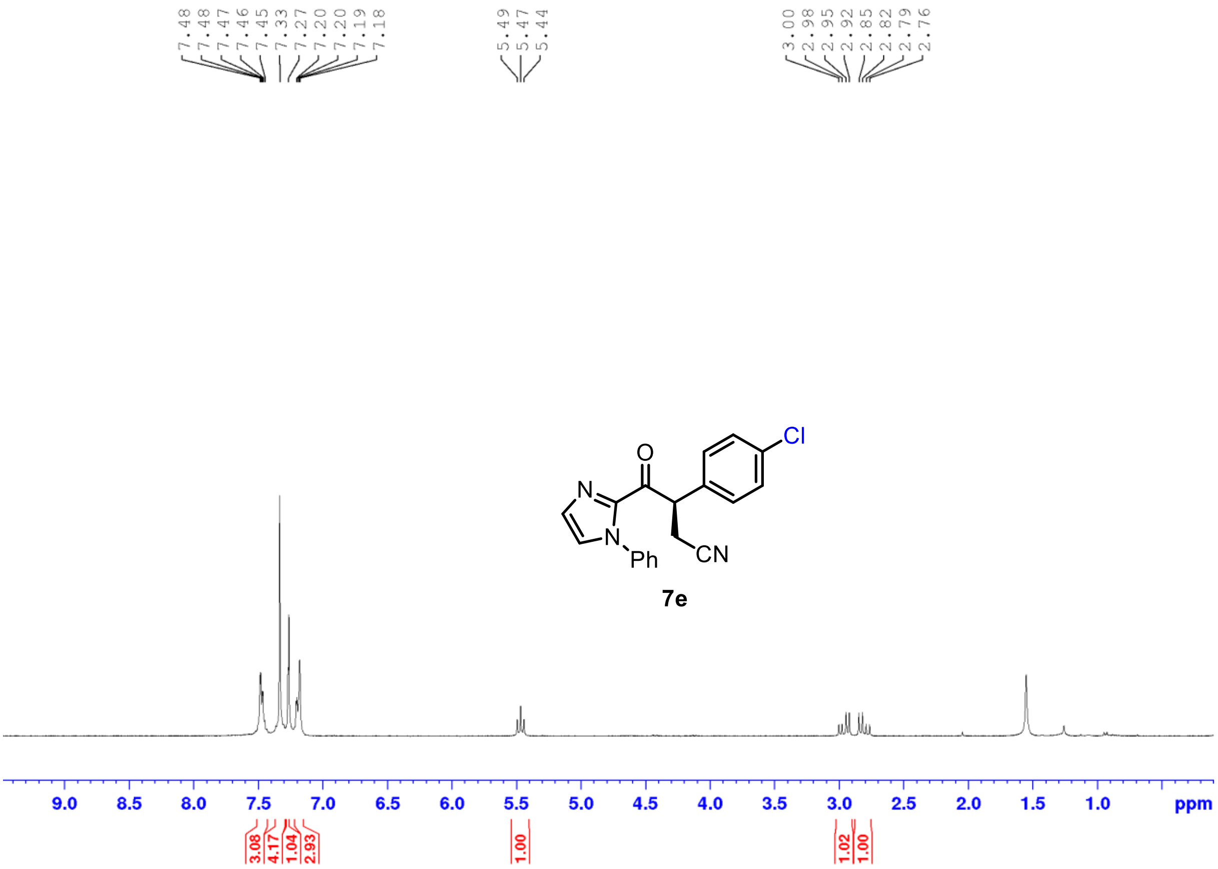

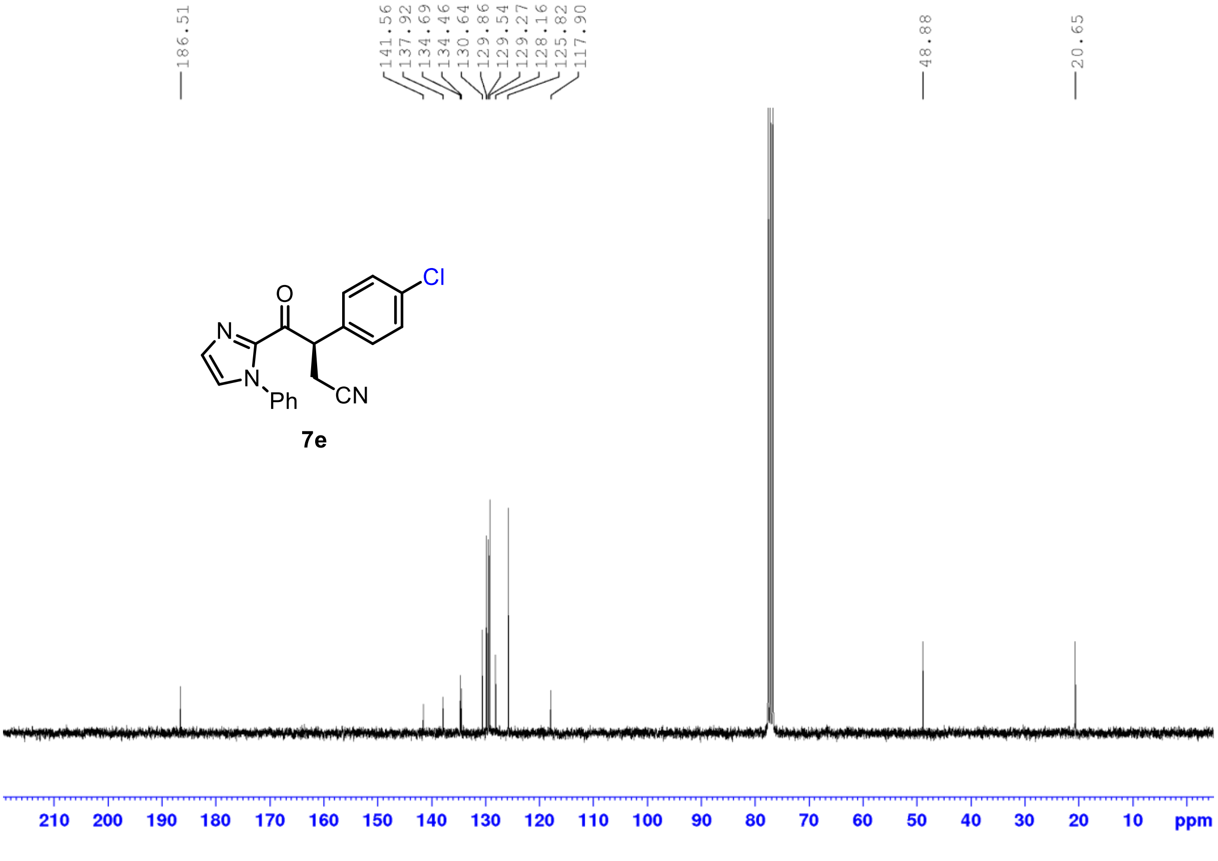

Figure S17. ^{1}H (300 MHz) and ^{13}C (75 MHz) spectra of **7e** in $CDCl_3$.

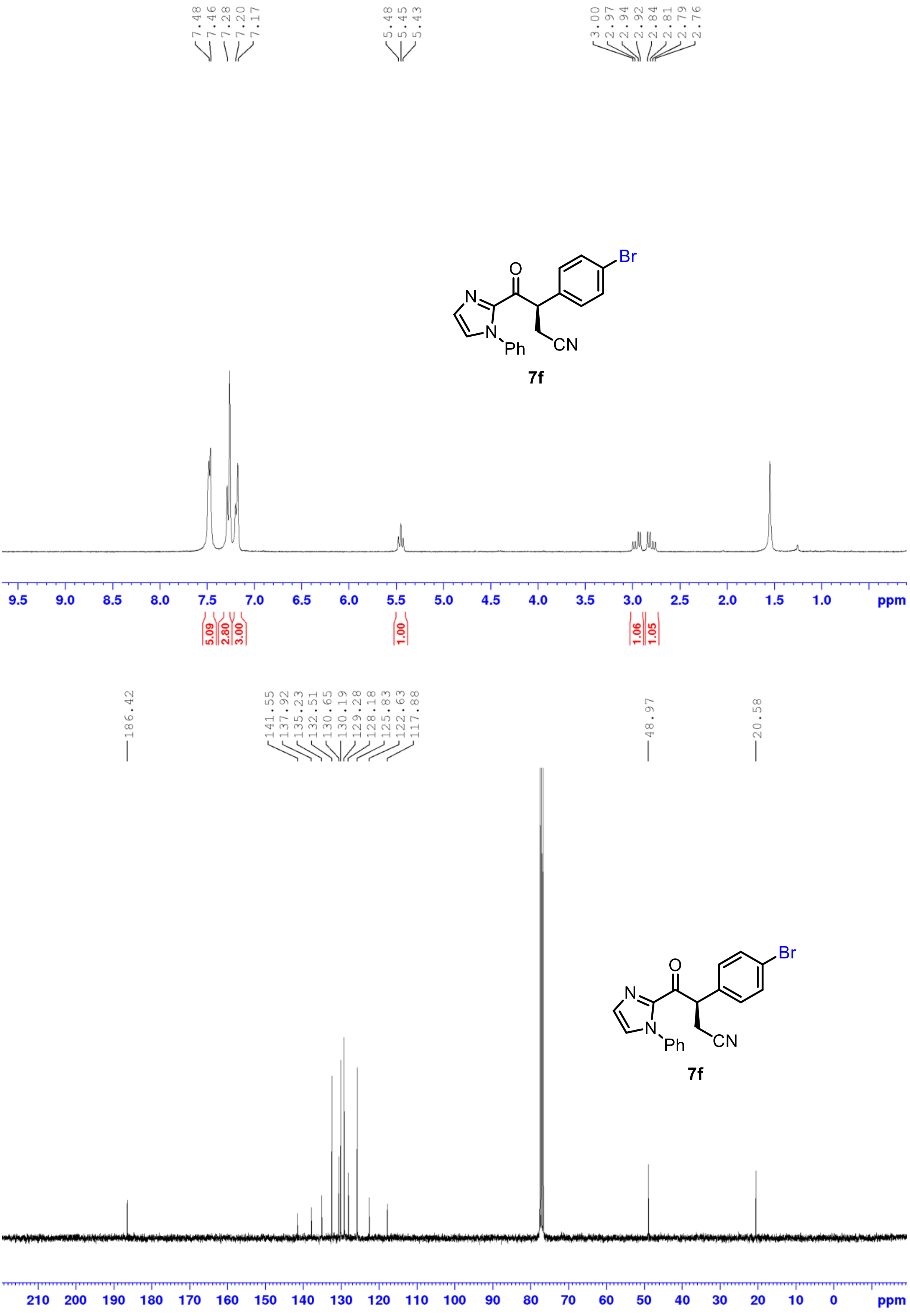

Figure S18. ^{1}H (300 MHz) and ^{13}C (75 MHz) spectra of **7f** in $CDCl_3$.

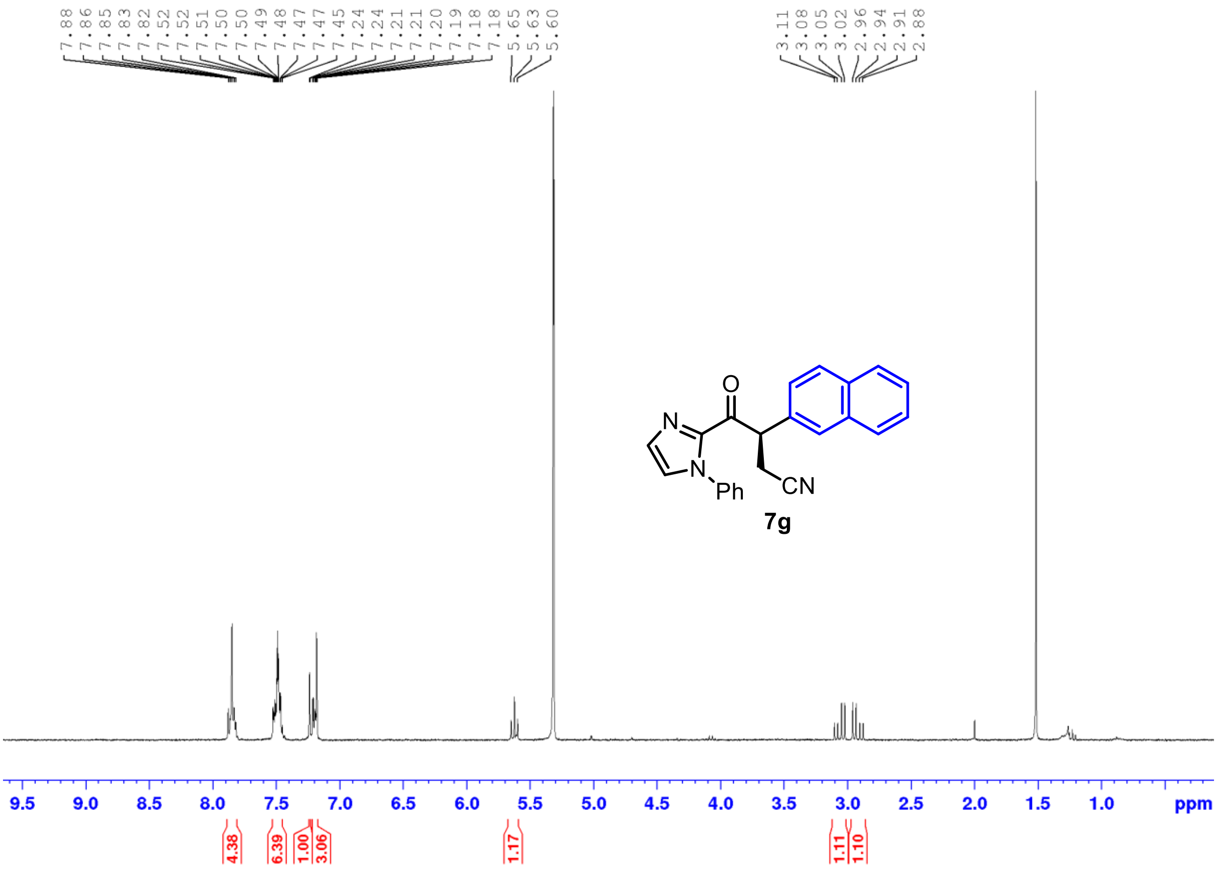

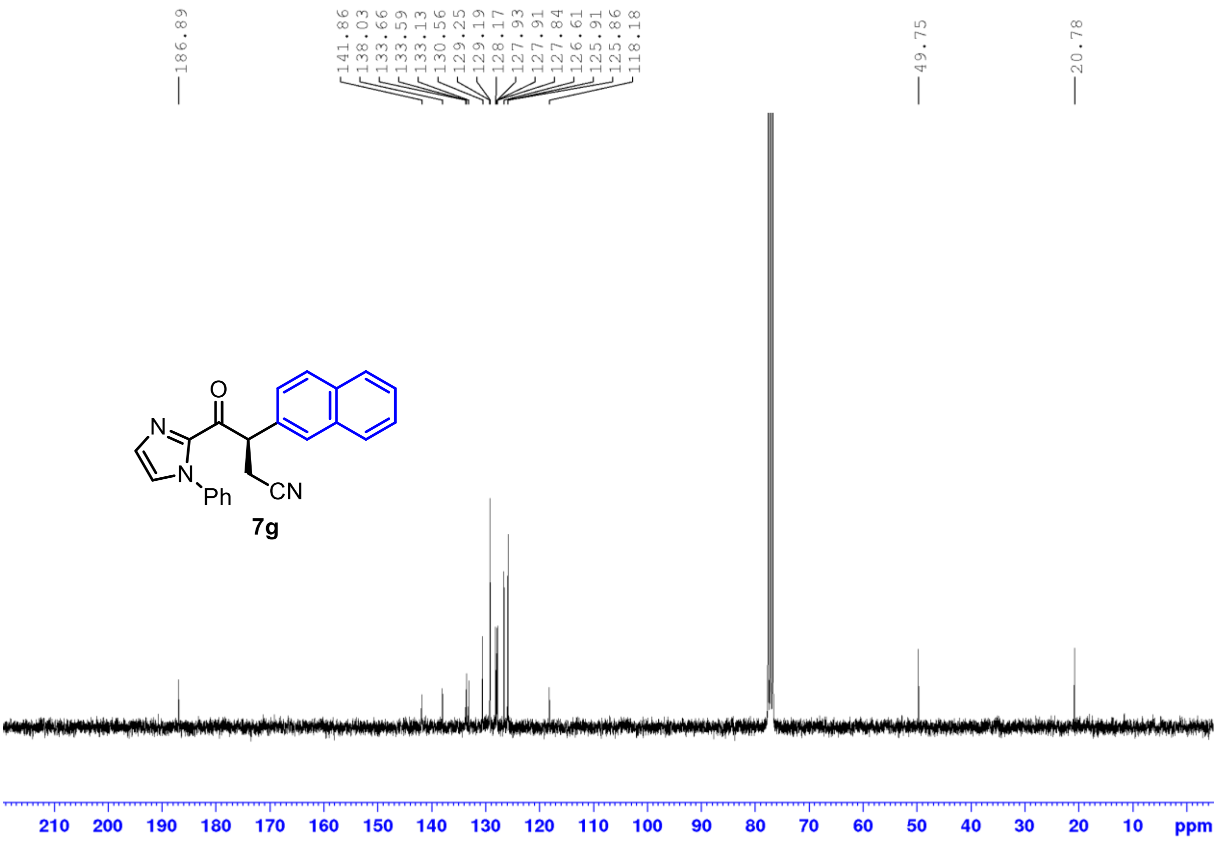

Figure S19. ^{1}H (300 MHz, CD_2Cl_2) and ^{13}C (75 MHz, $CDCl_3$) spectra of **7g**.

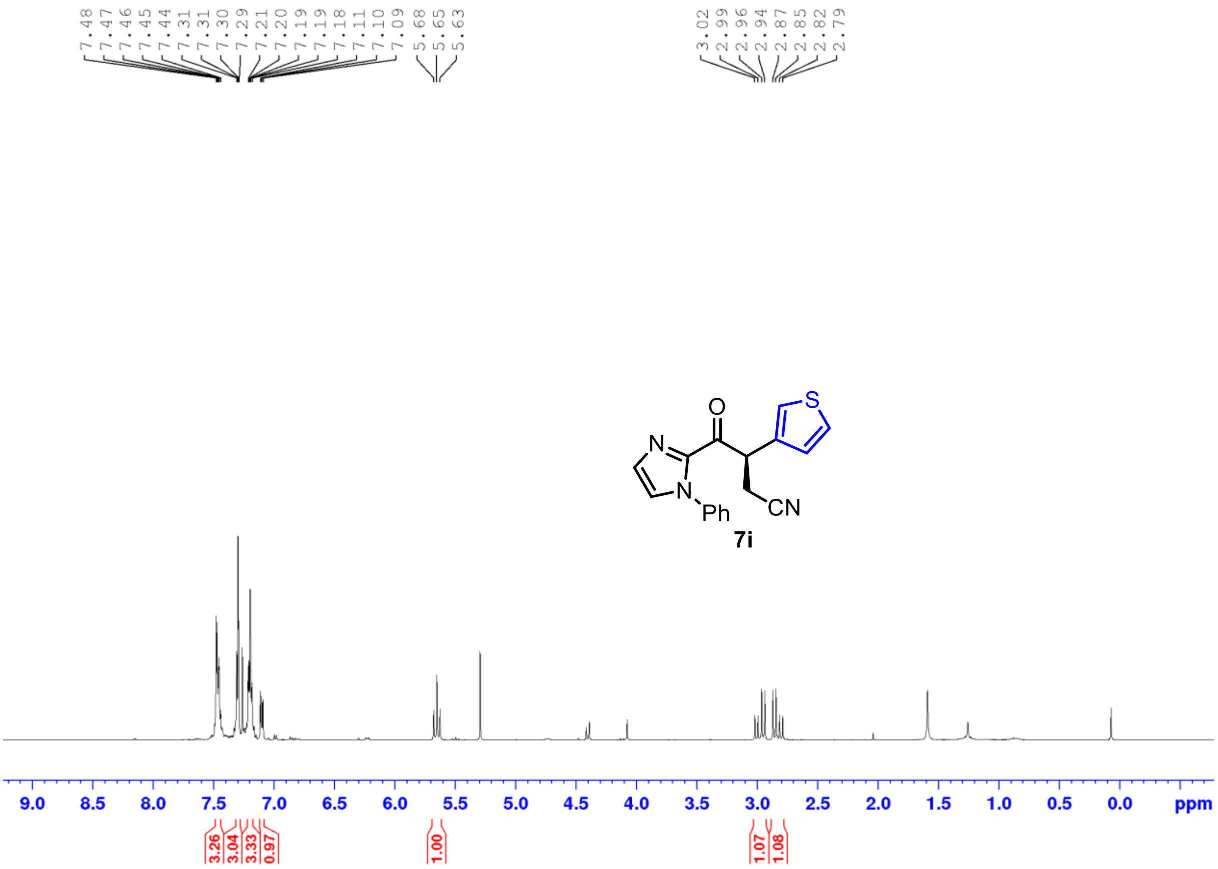

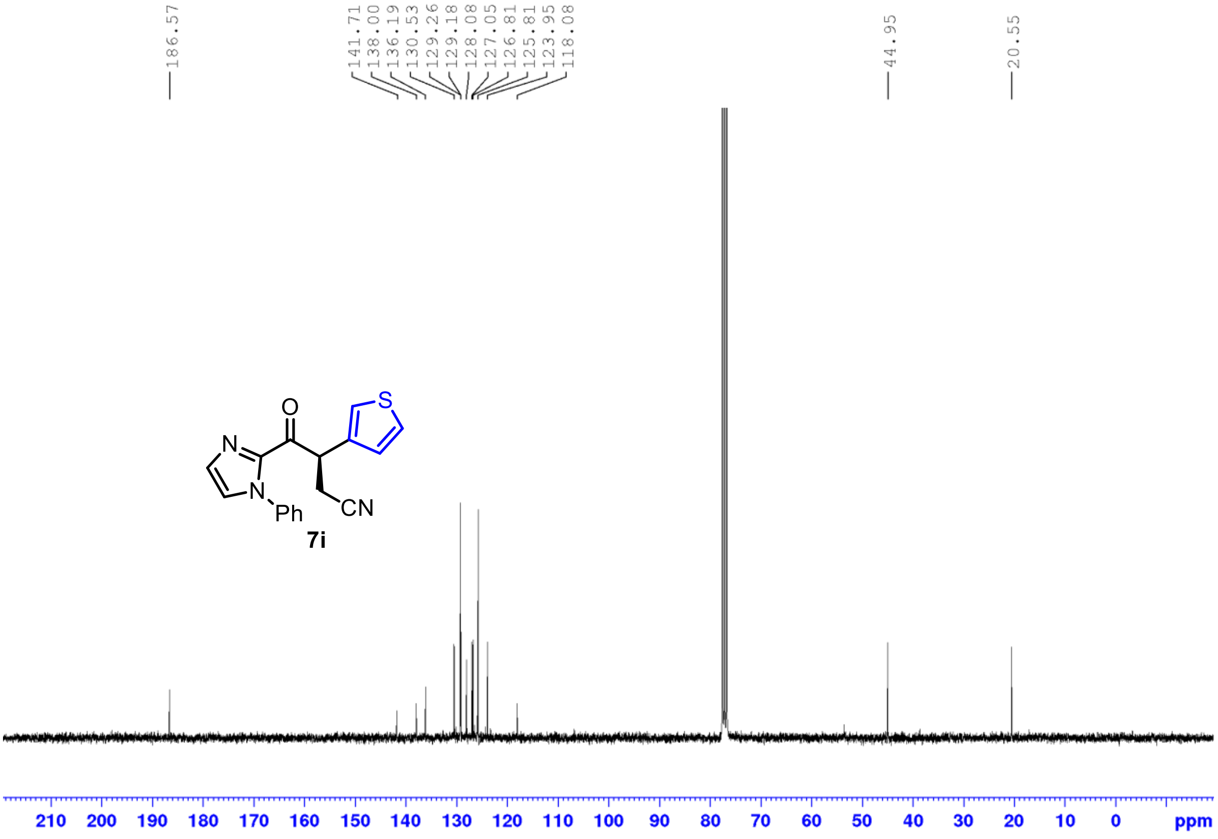

Figure S20. ^{1}H (300 MHz) and ^{13}C (75 MHz) spectra of **7i** in $CDCl_3$.

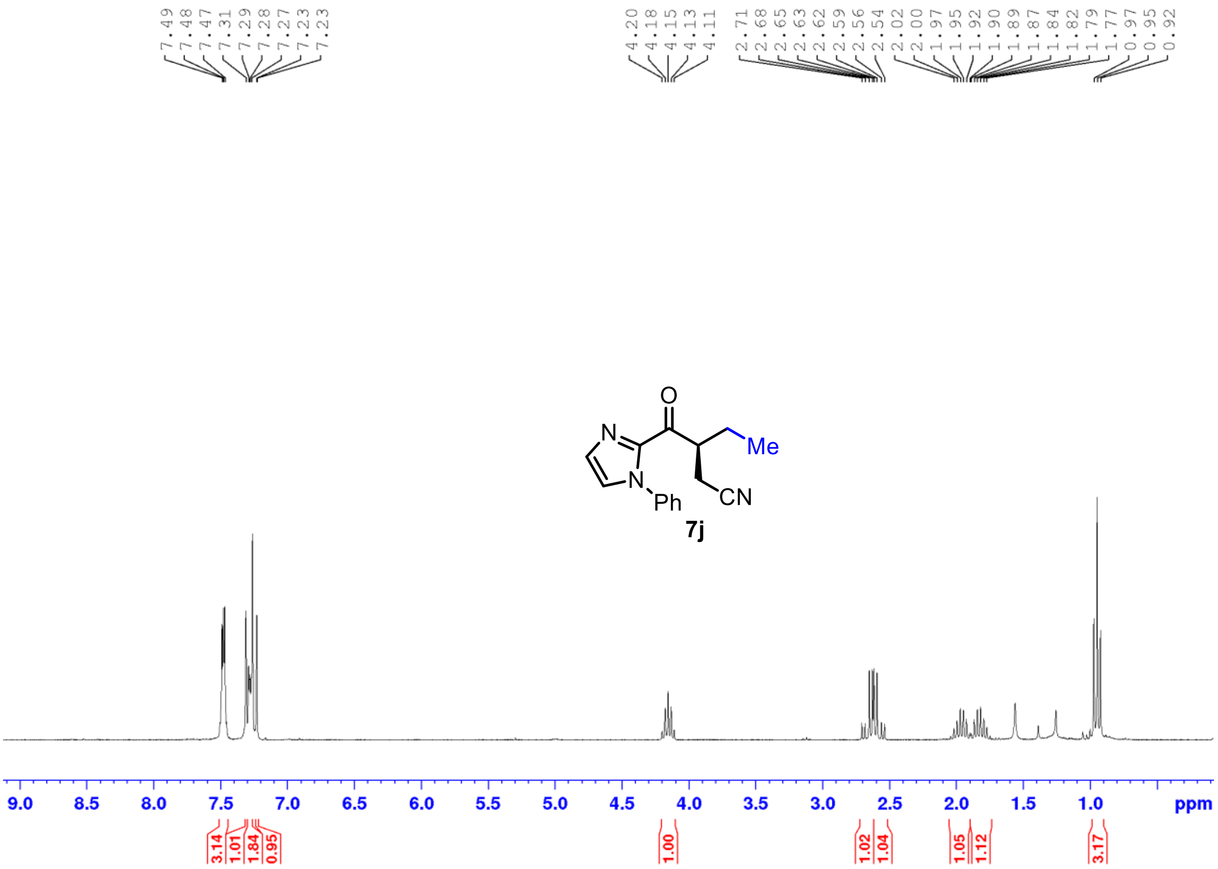

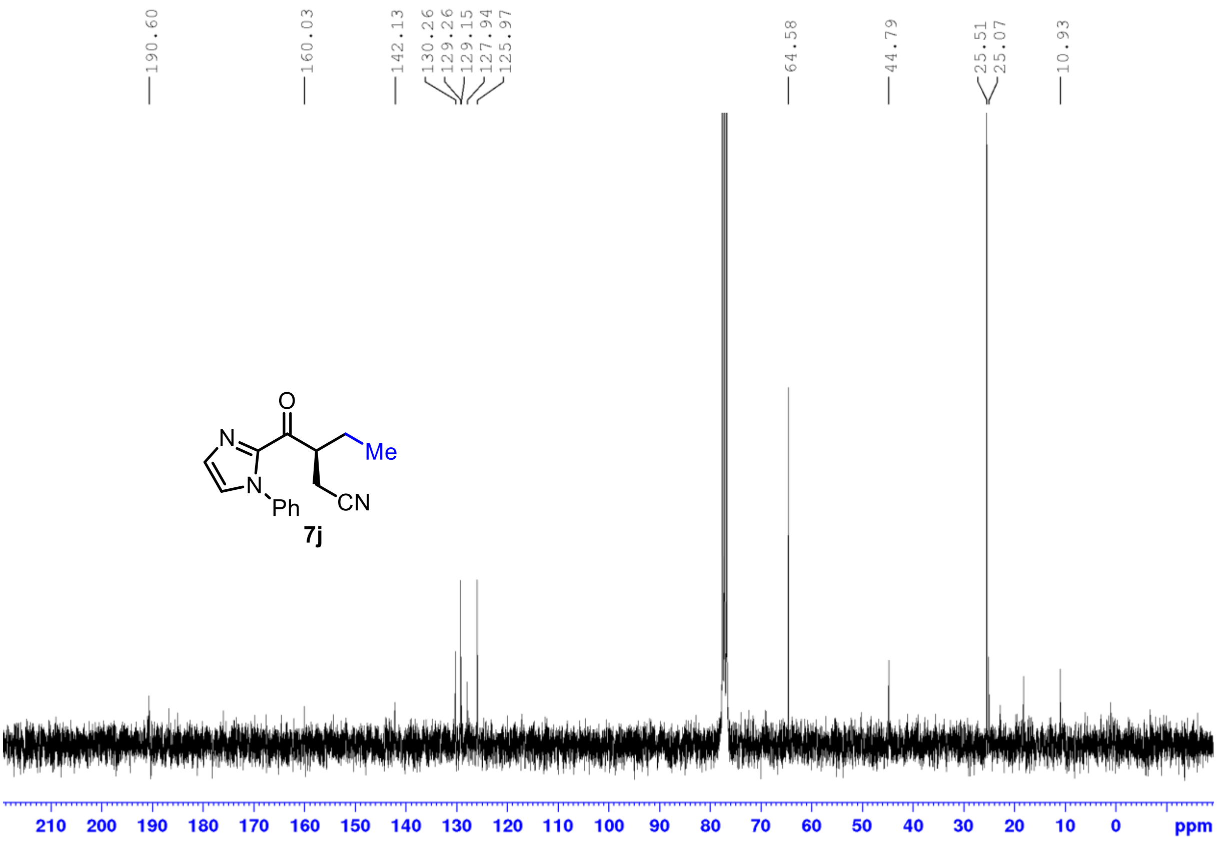

Figure S21. ^{1}H (300 MHz) and ^{13}C (75 MHz) spectra of **7j** in $CDCl_3$.

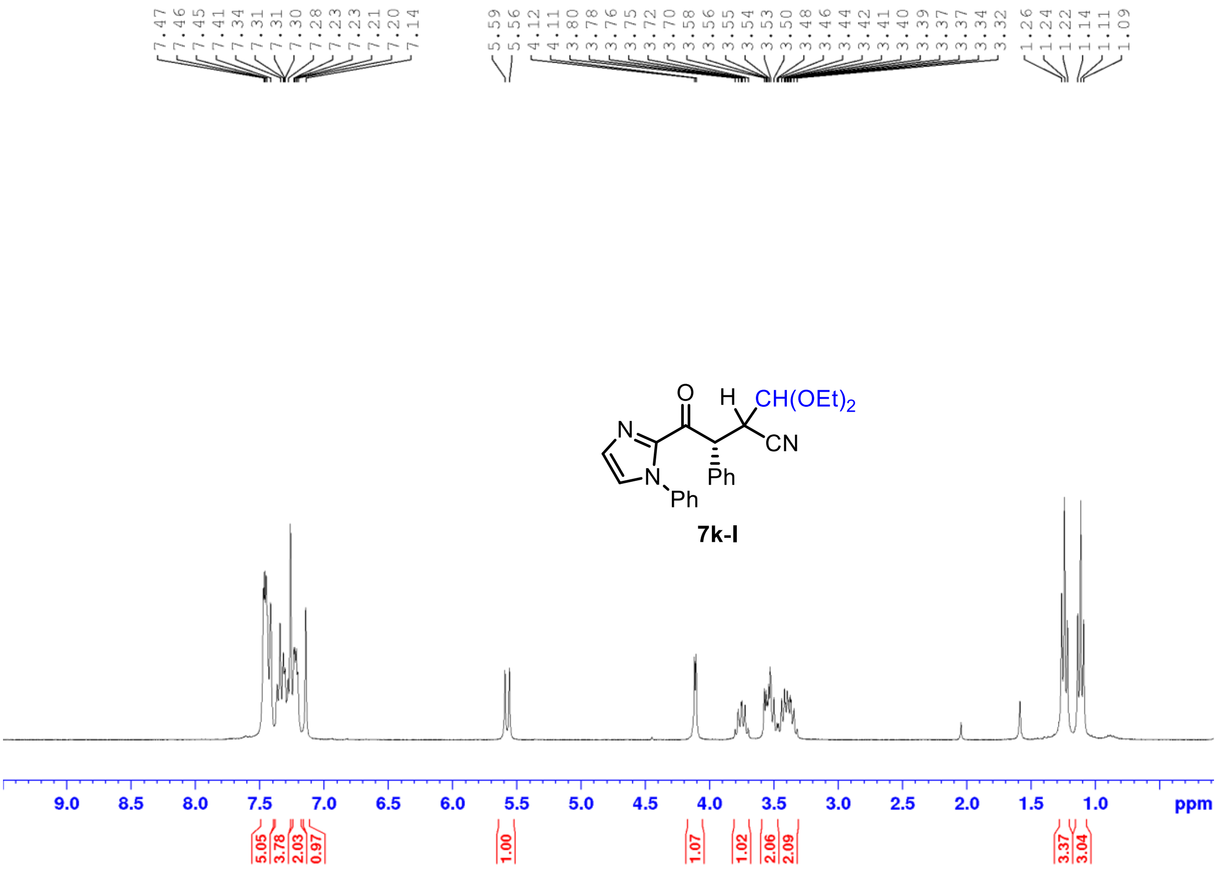

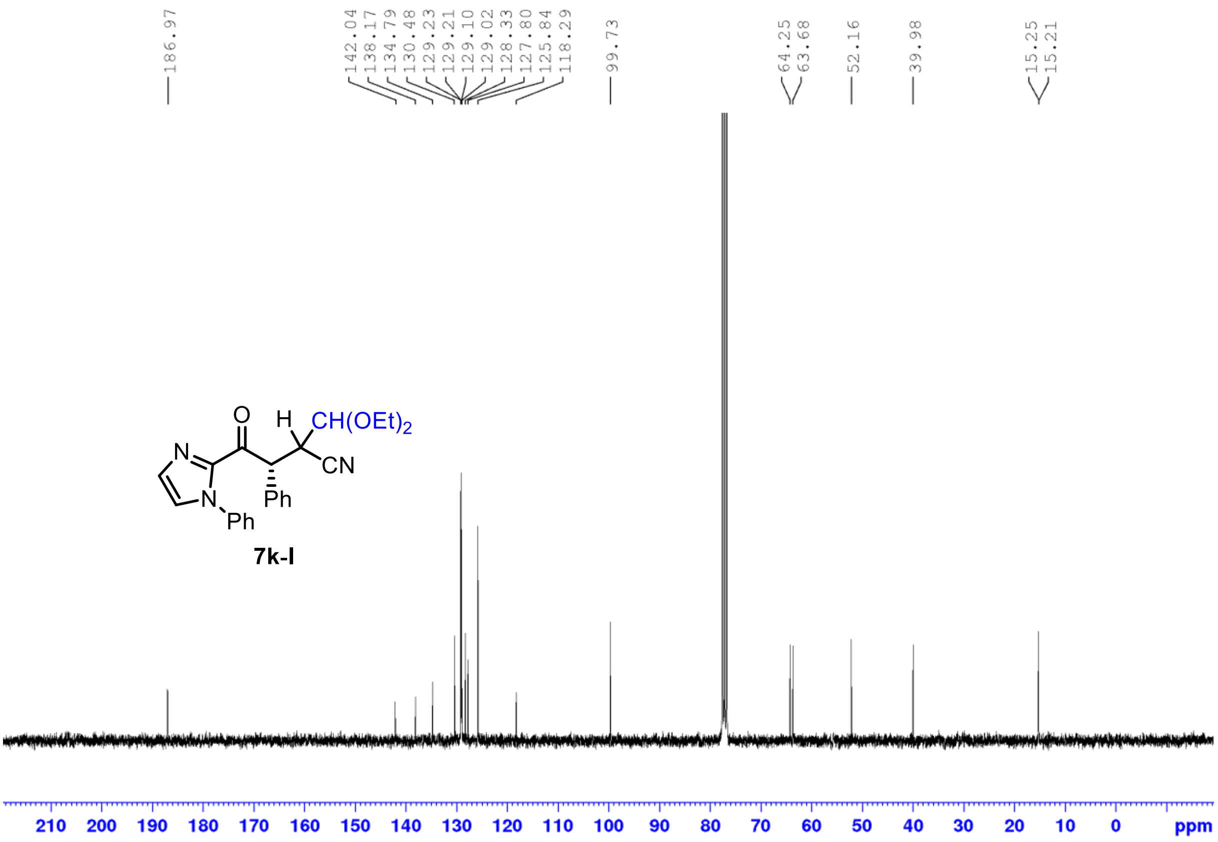

Figure S22. ^{1}H (300 MHz) and ^{13}C (75 MHz) spectra of **7k-I** in $CDCl_3$.

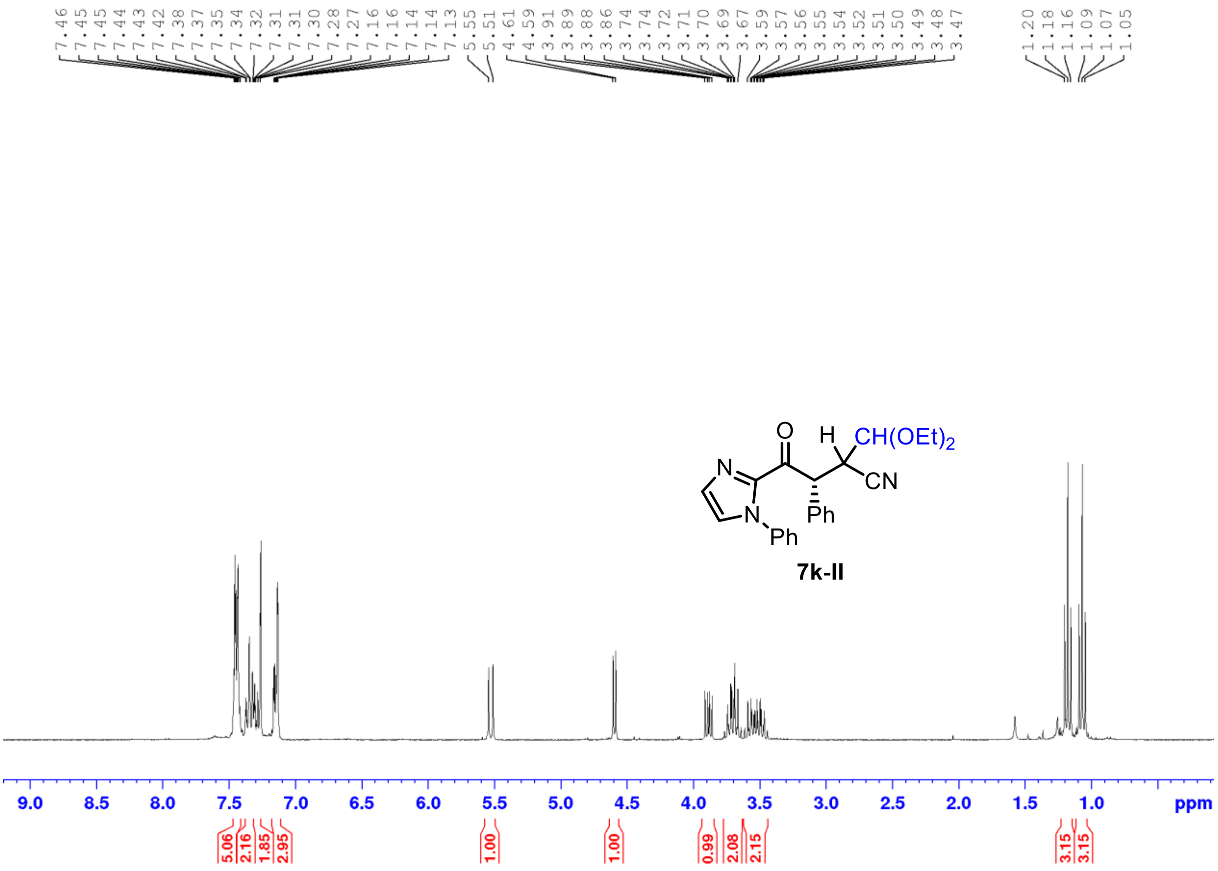

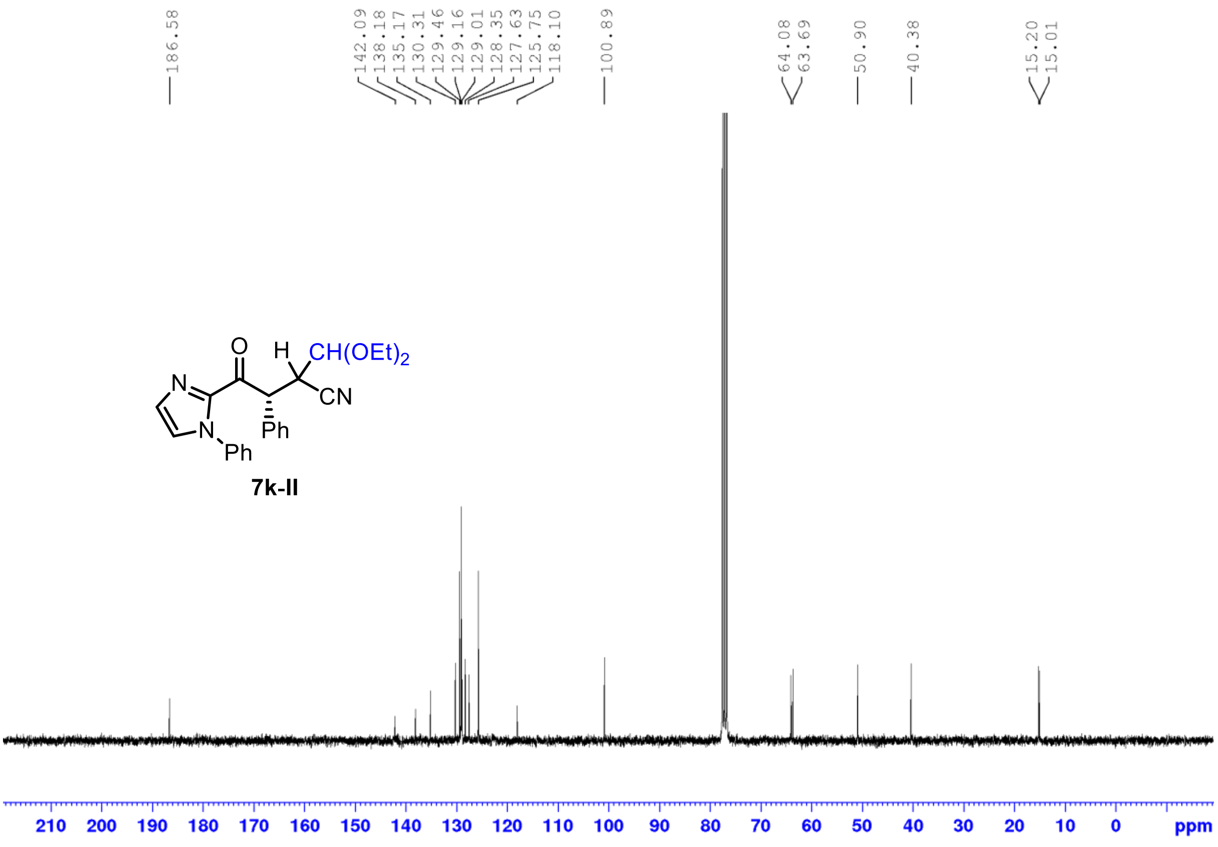

Figure S23. ^{1}H (300 MHz) and ^{13}C (75 MHz) spectra of **7k-II** in $CDCl_3$.

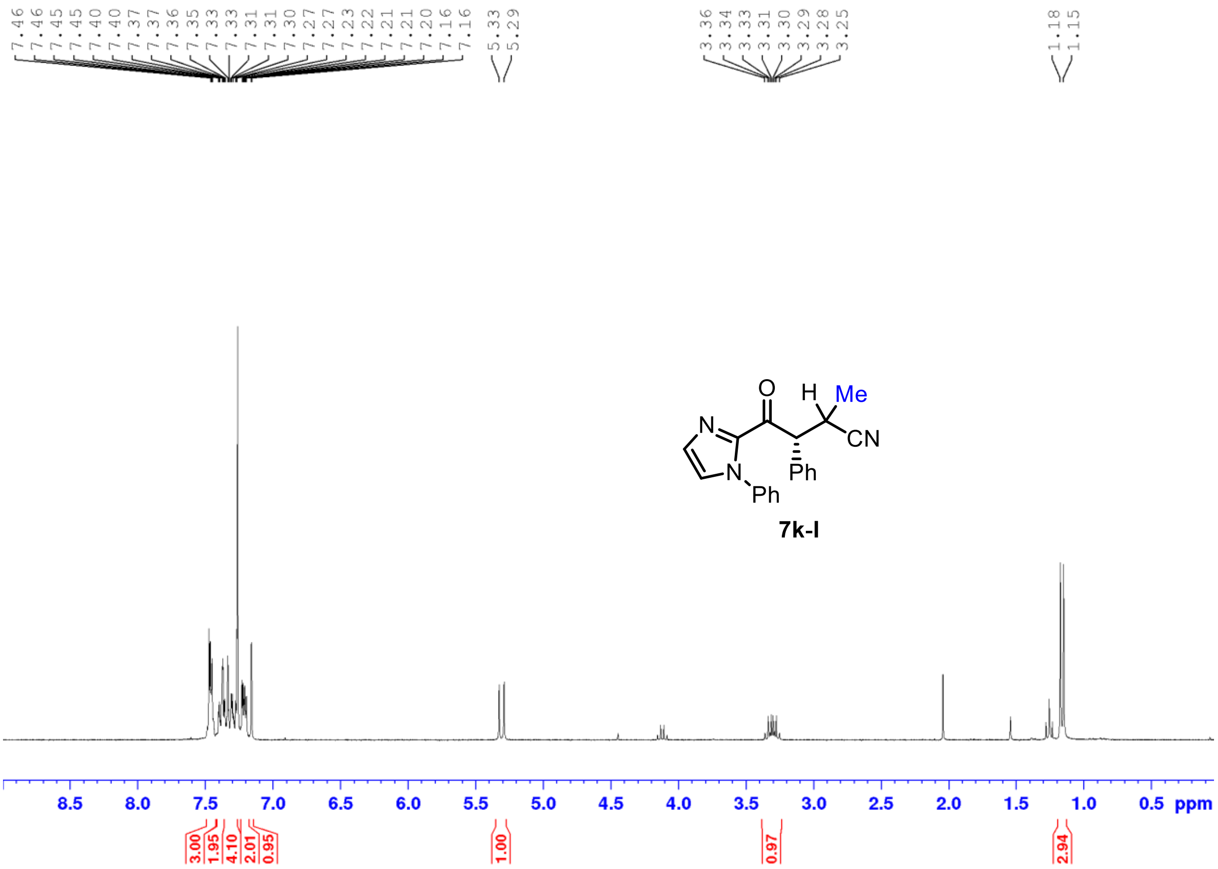

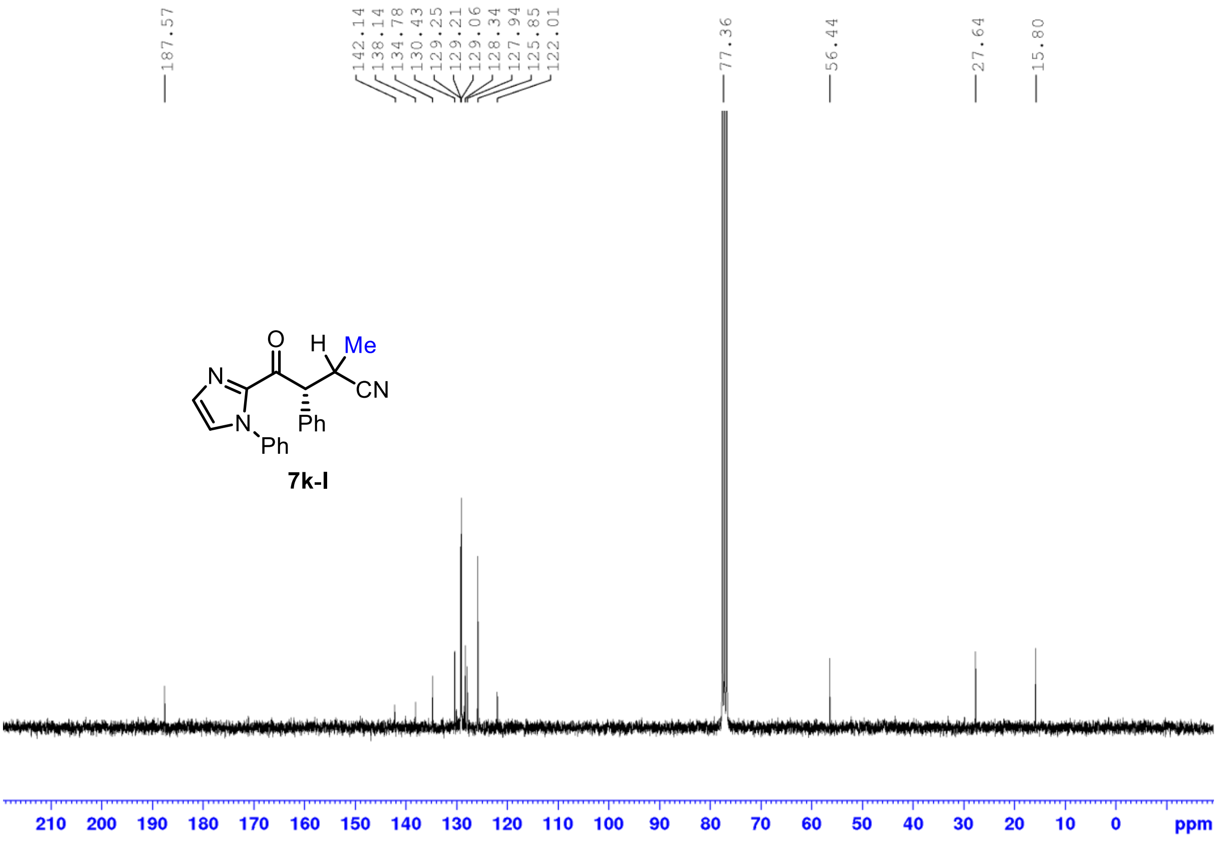

Figure S24. ^{1}H (300 MHz) and ^{13}C (75 MHz) spectra of **7l-l** in $CDCl_3$.

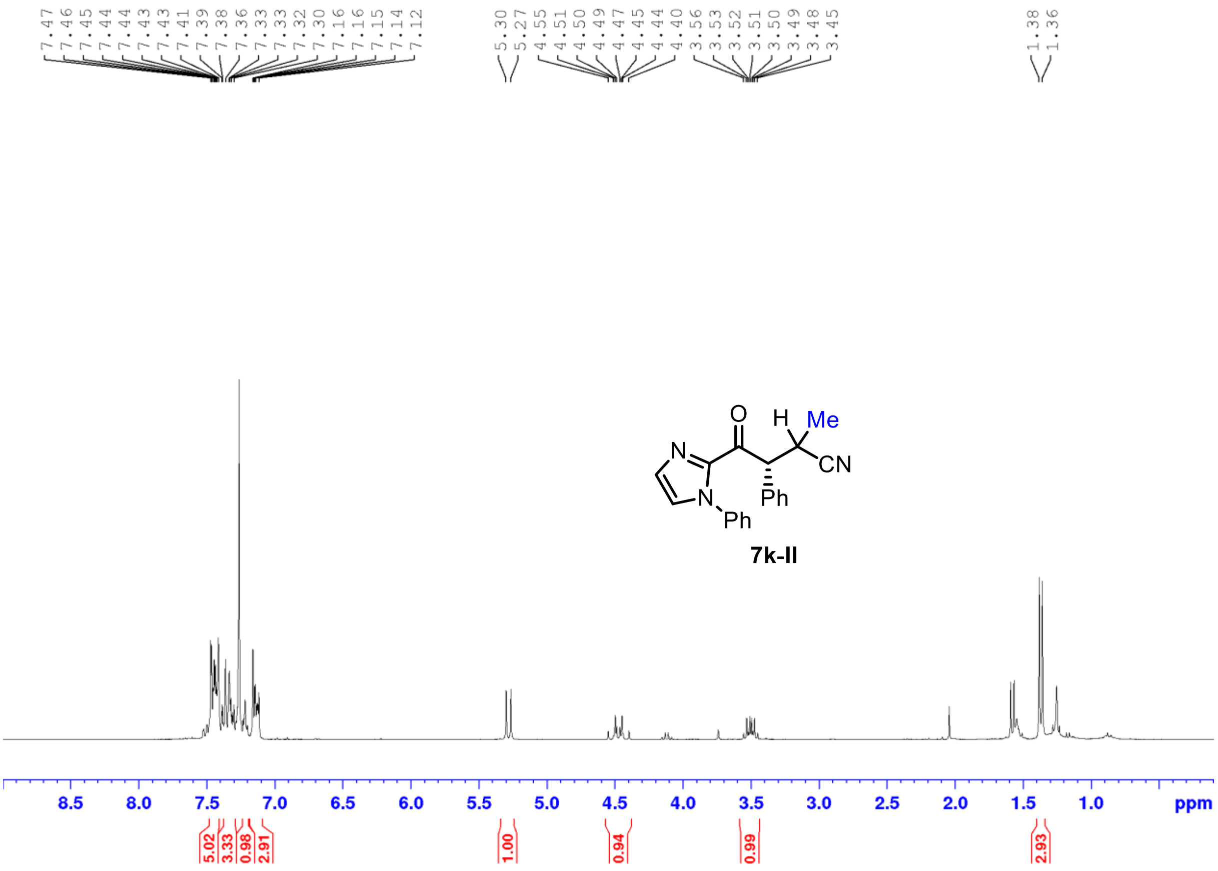

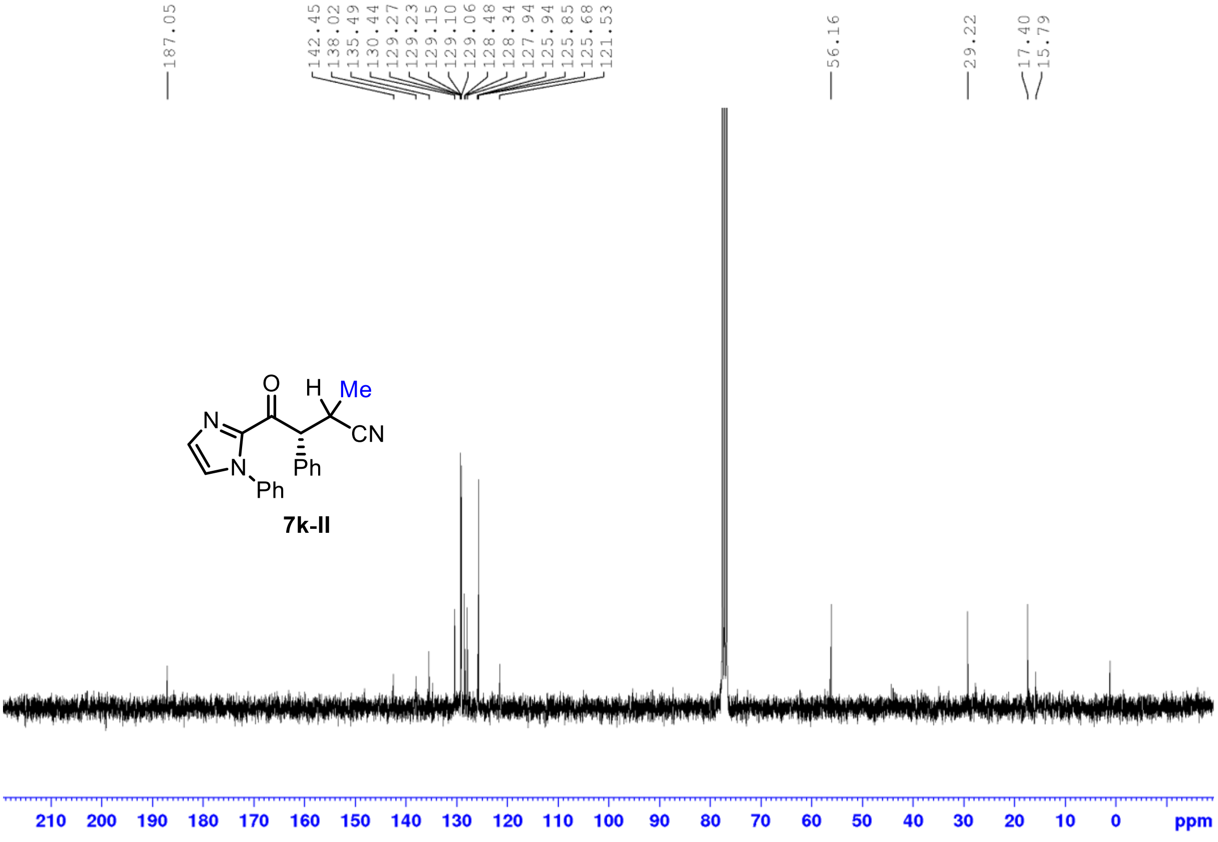

Figure S25. ^{1}H (300 MHz) and ^{13}C (75 MHz) spectra of **7l-II** in $CDCl_3$.

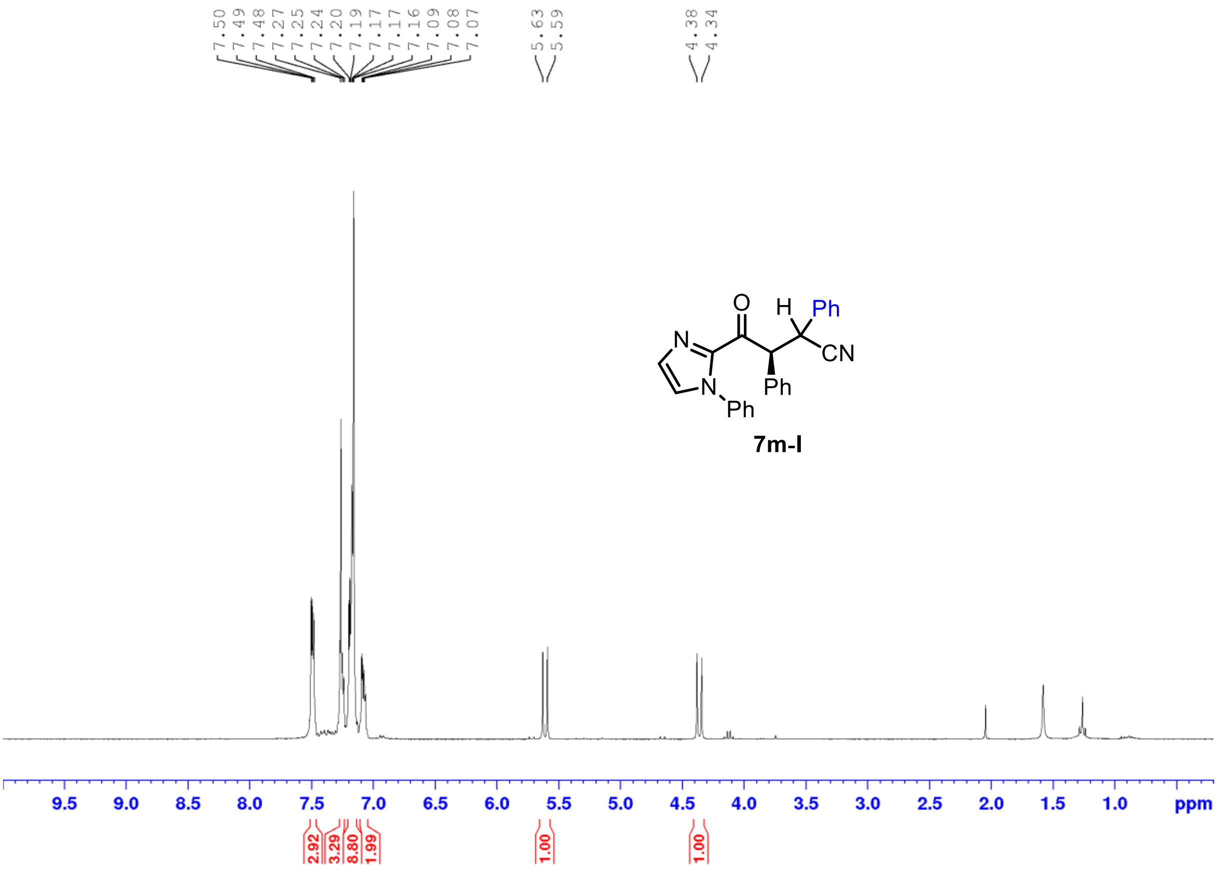

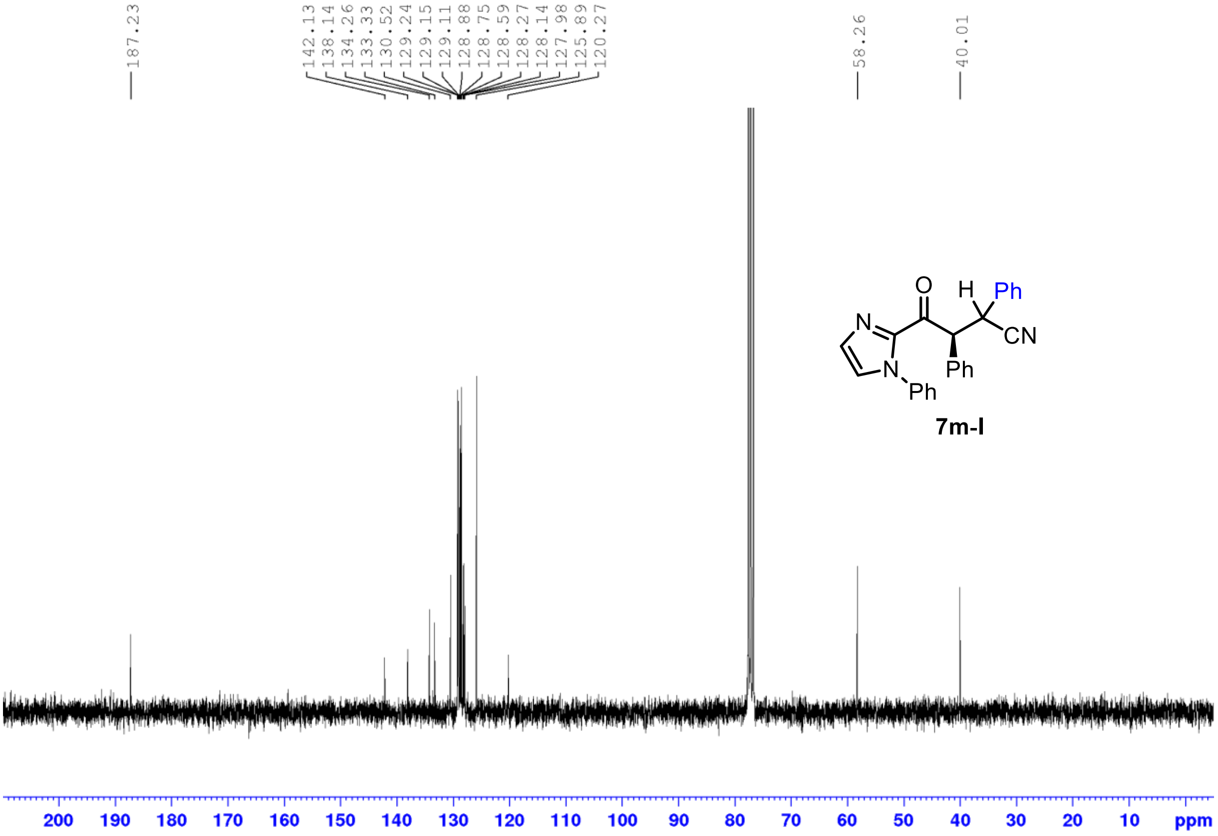

Figure S 26. ^{1}H (300 MHz) and ^{13}C (75 MHz) spectra of **7m-I** in $CDCl_3$.

6 Chiral HPLC Traces

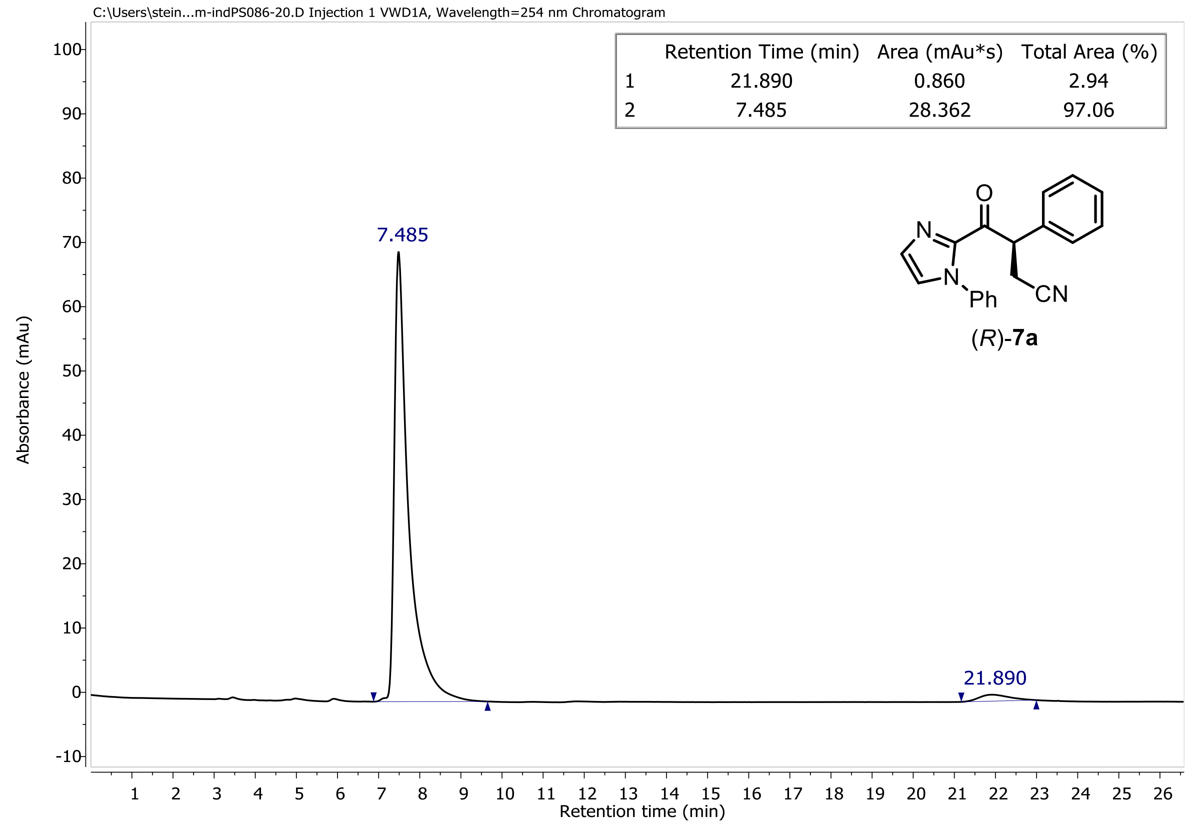

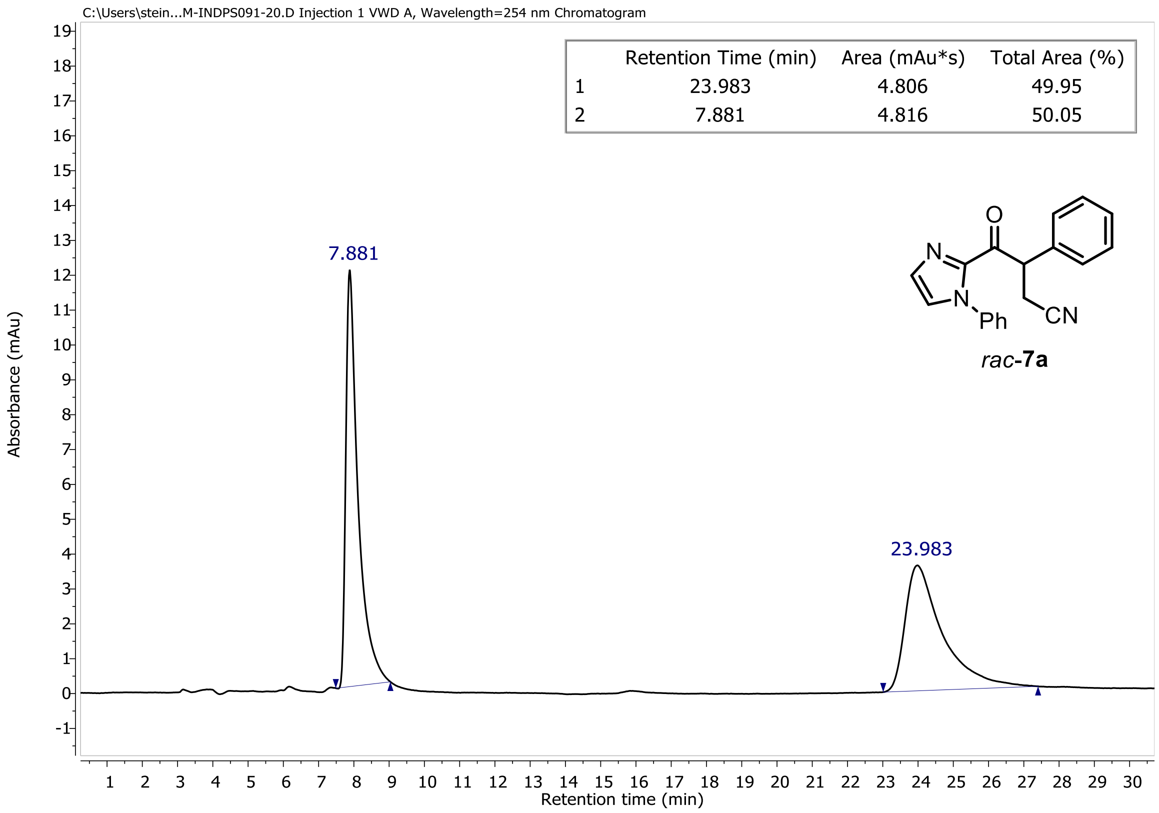

Figure S27. HPLC traces of (*R*)-**7a** (94% *ee*) and *rac*-**7a**.

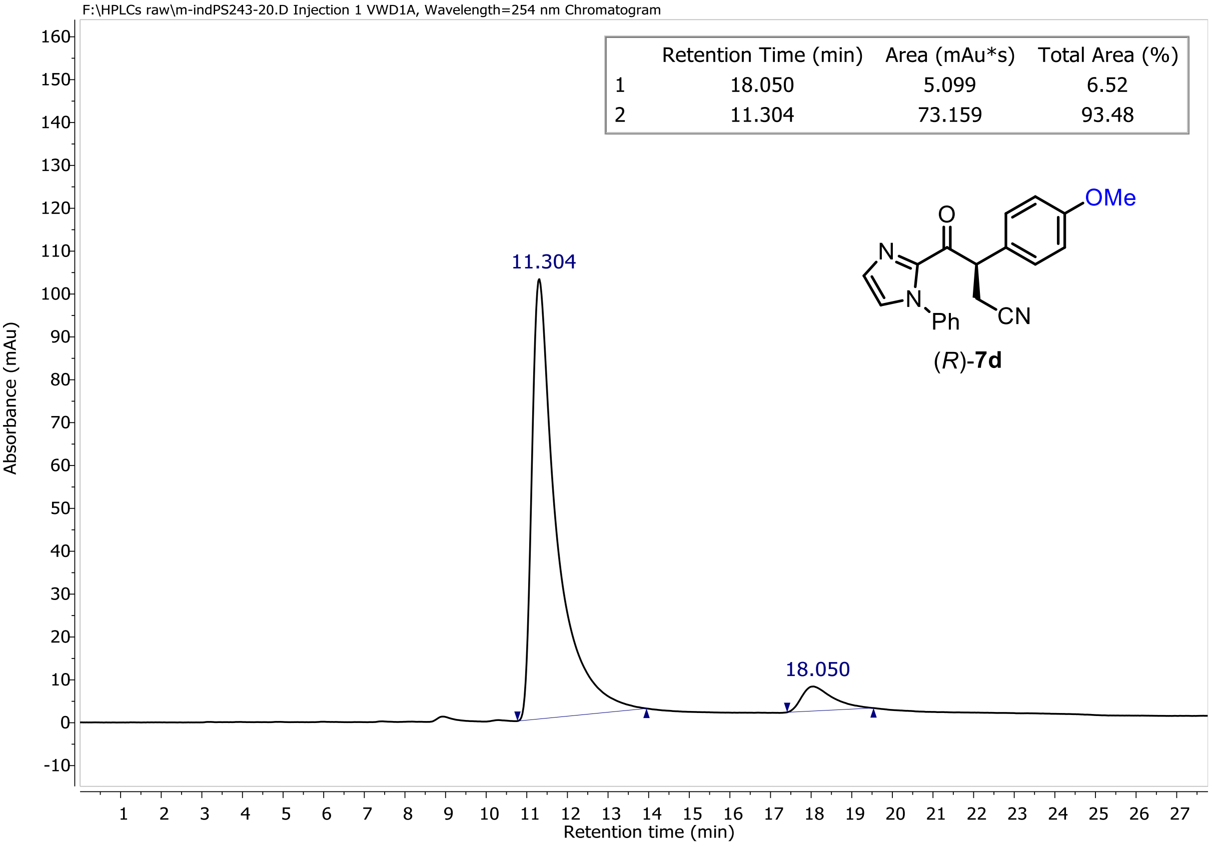

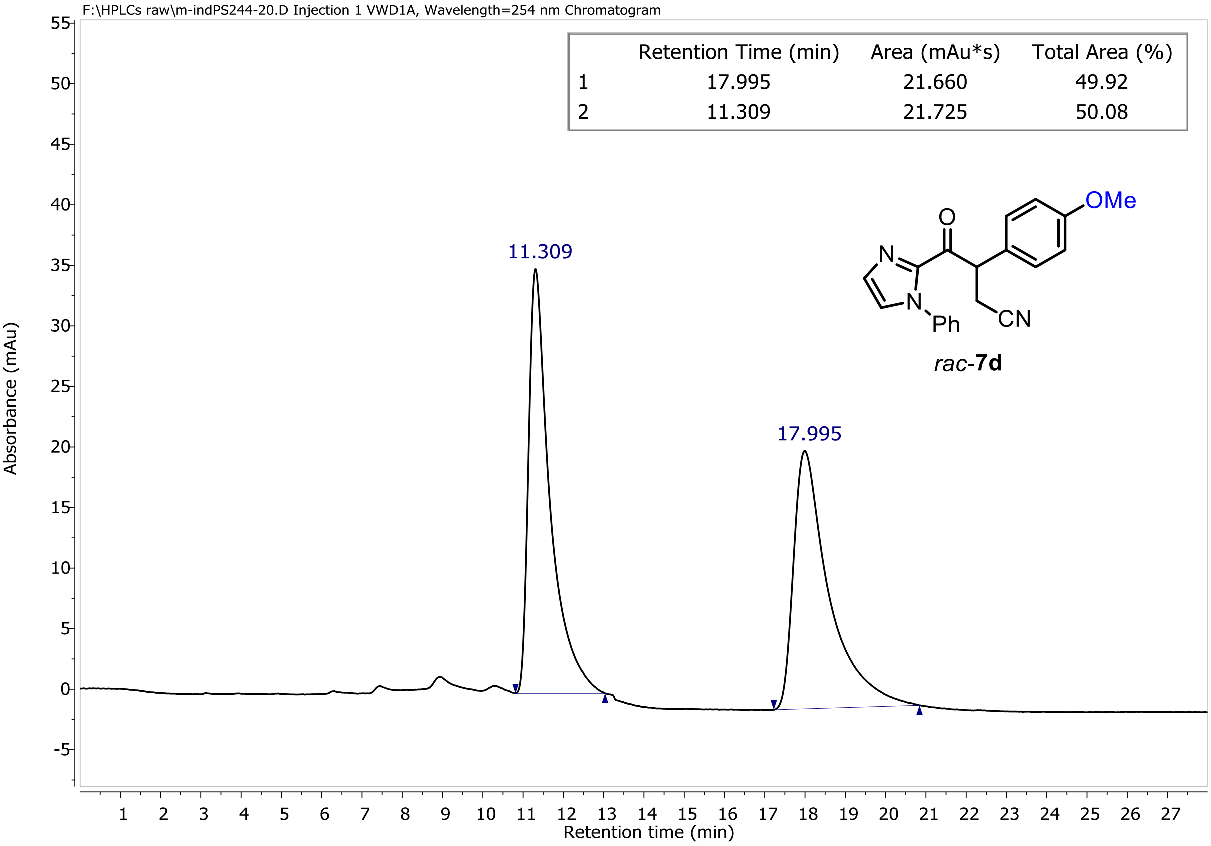

Figure S28. HPLC traces of (*R*)-**7d** (87% *ee*) and *rac*-**7d**.

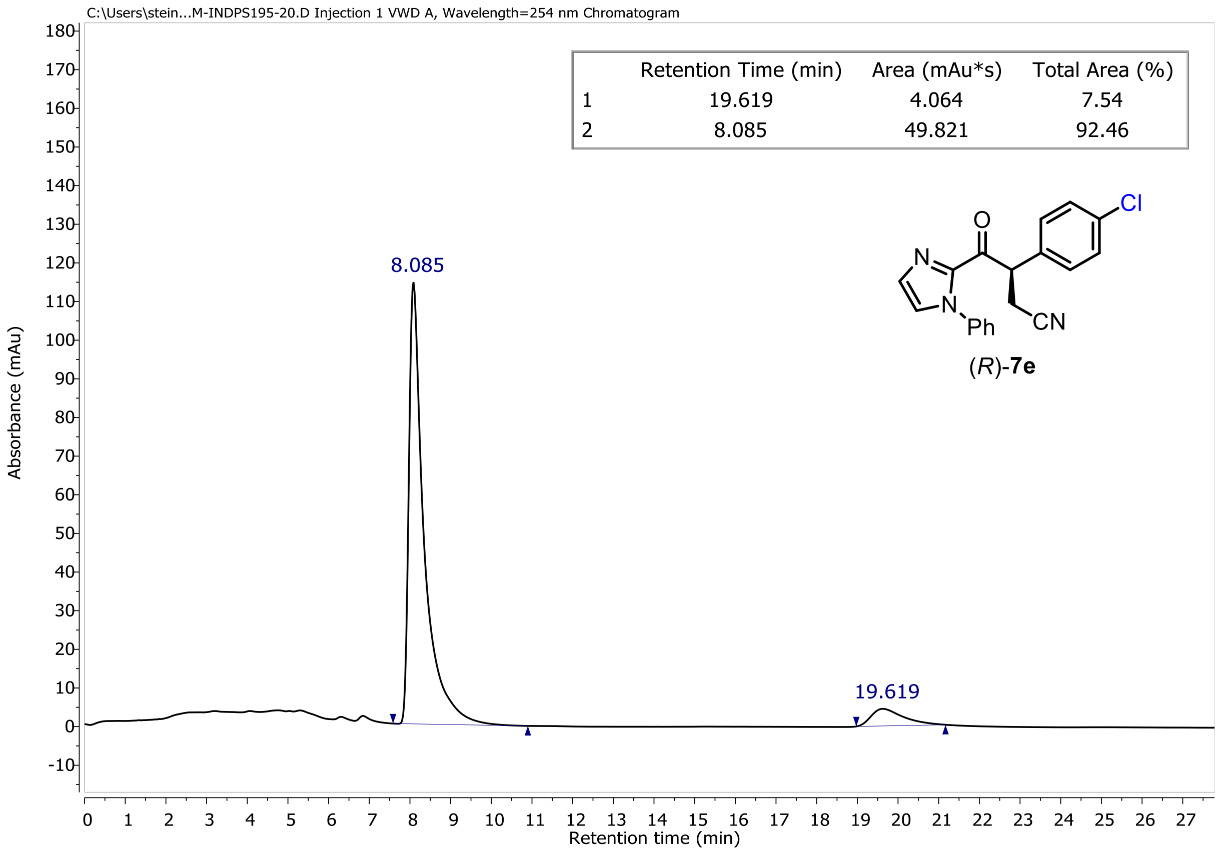

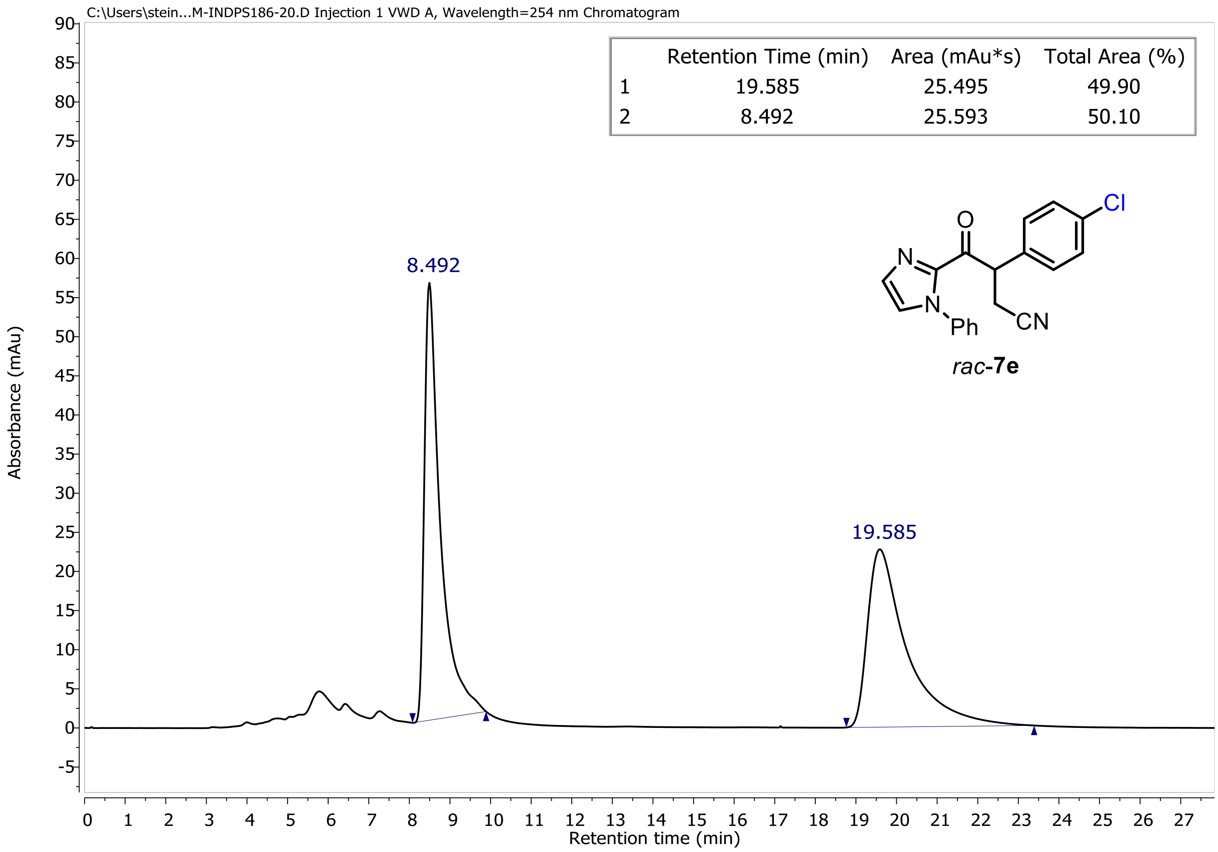

Figure S29. HPLC traces of (*R*)-**7e** (85% *ee*) and *rac*-**7e**.

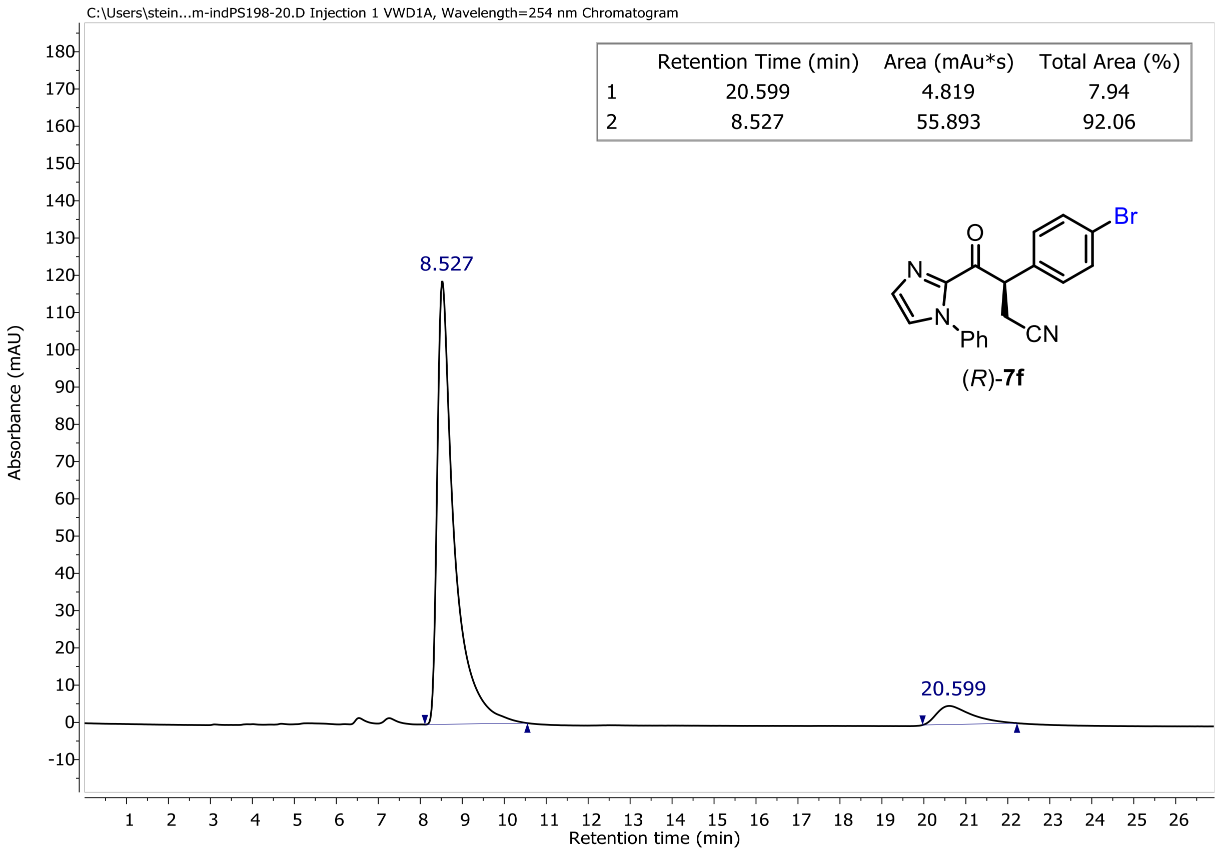

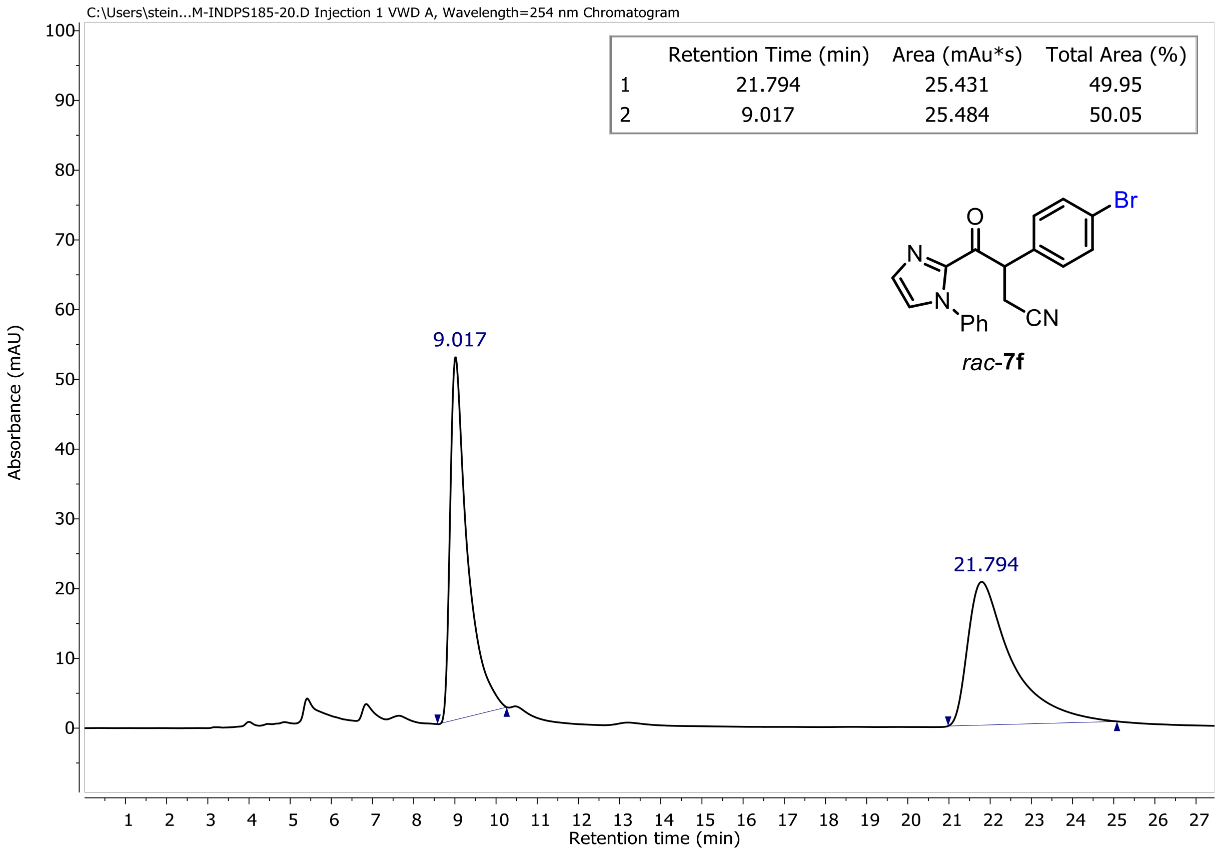

Figure S30. HPLC traces of (*R*)-**7f** (84% *ee*) and *rac*-**7f**.

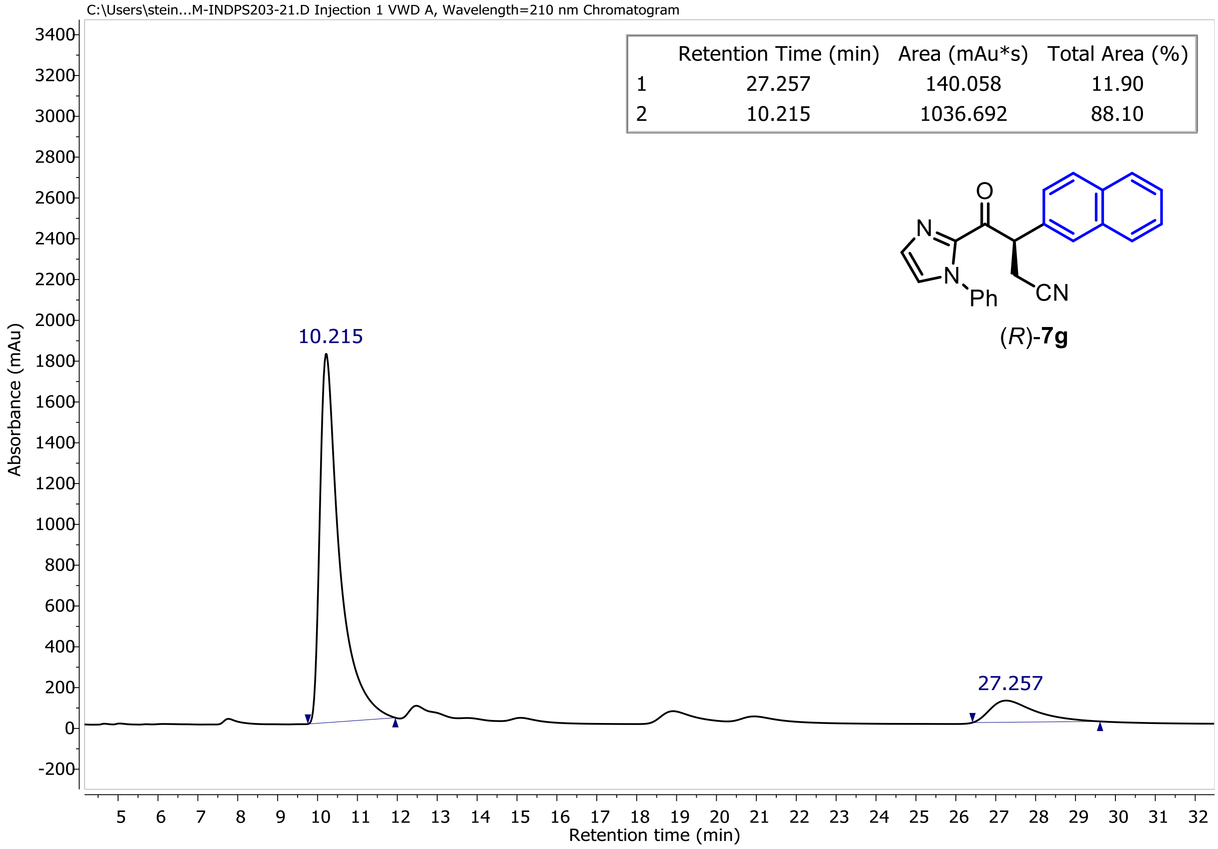

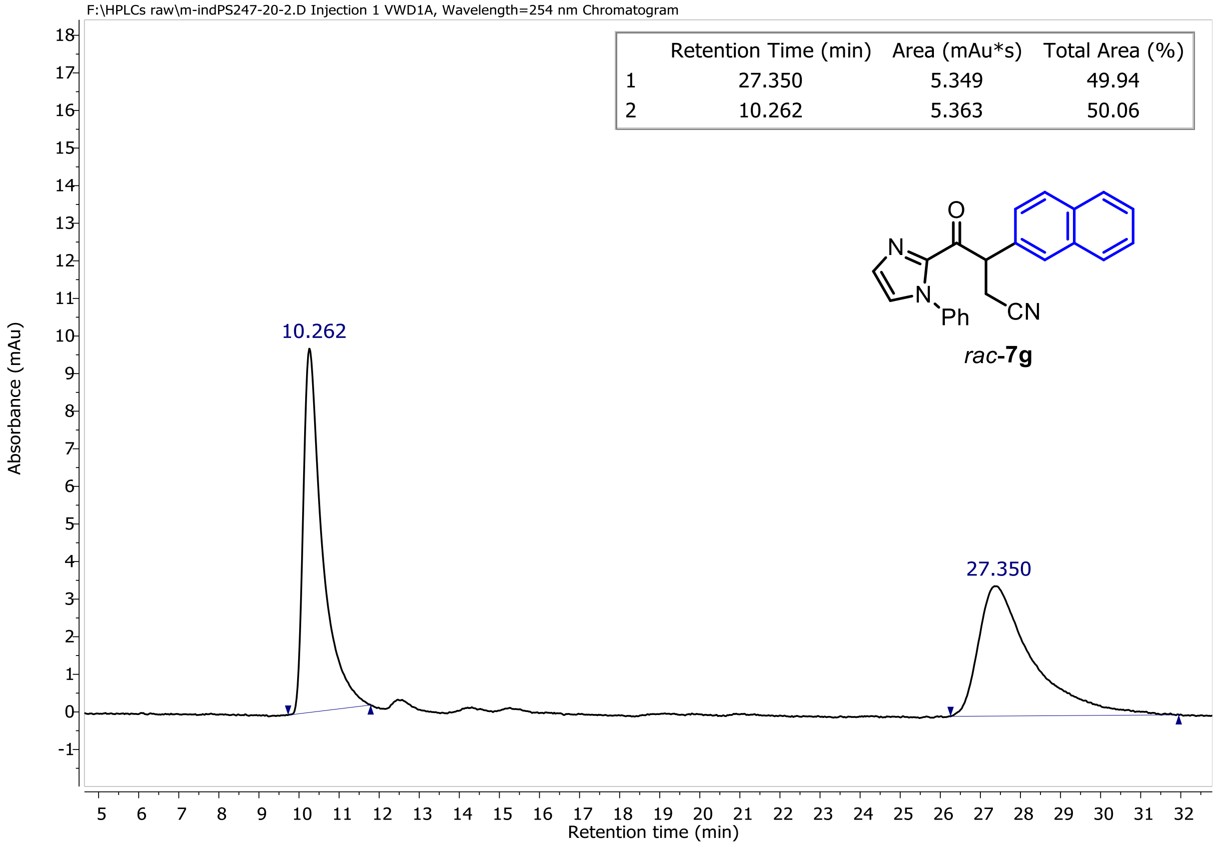

Figure S31. HPLC traces of (*R*)-**7g** (76% *ee*) and *rac*-**7g**.

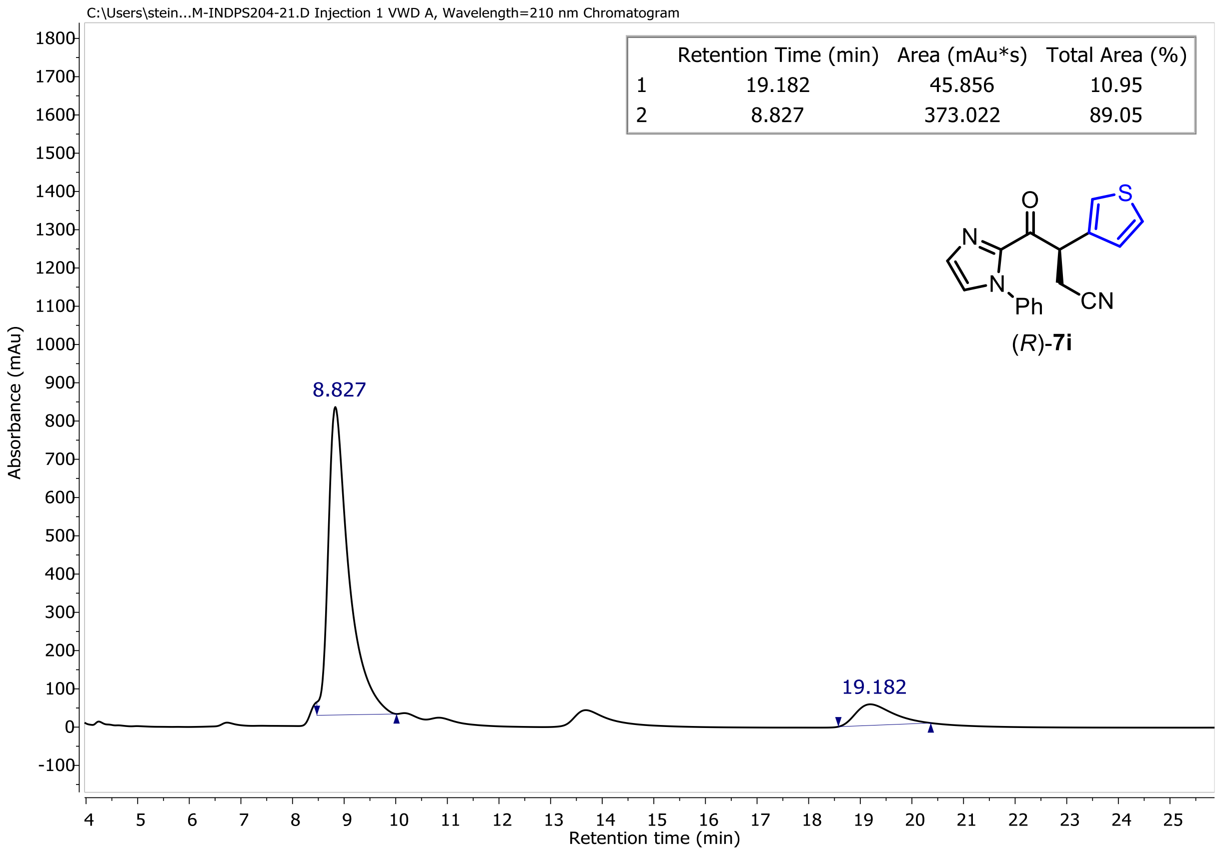

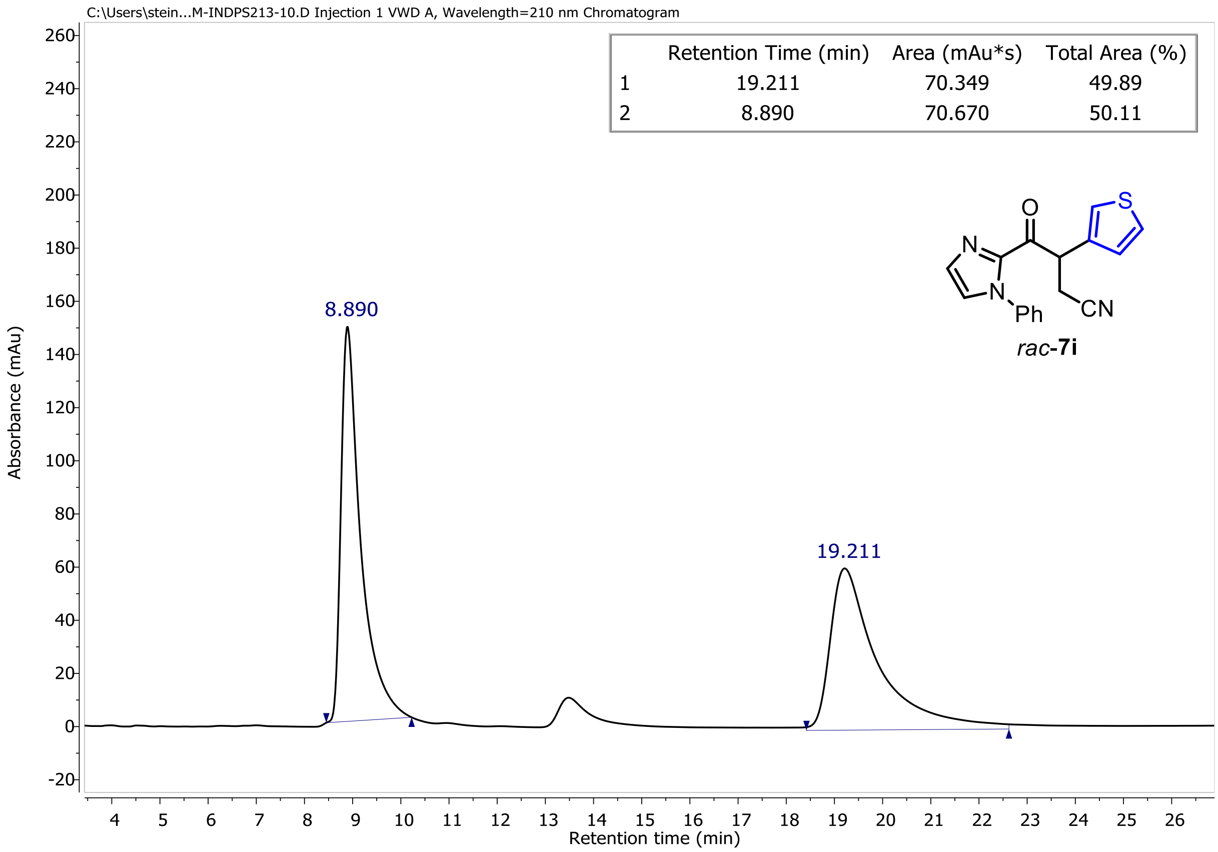

Figure S32. HPLC traces of (*R*)-**7i** (78% *ee*) and *rac*-**7i**.

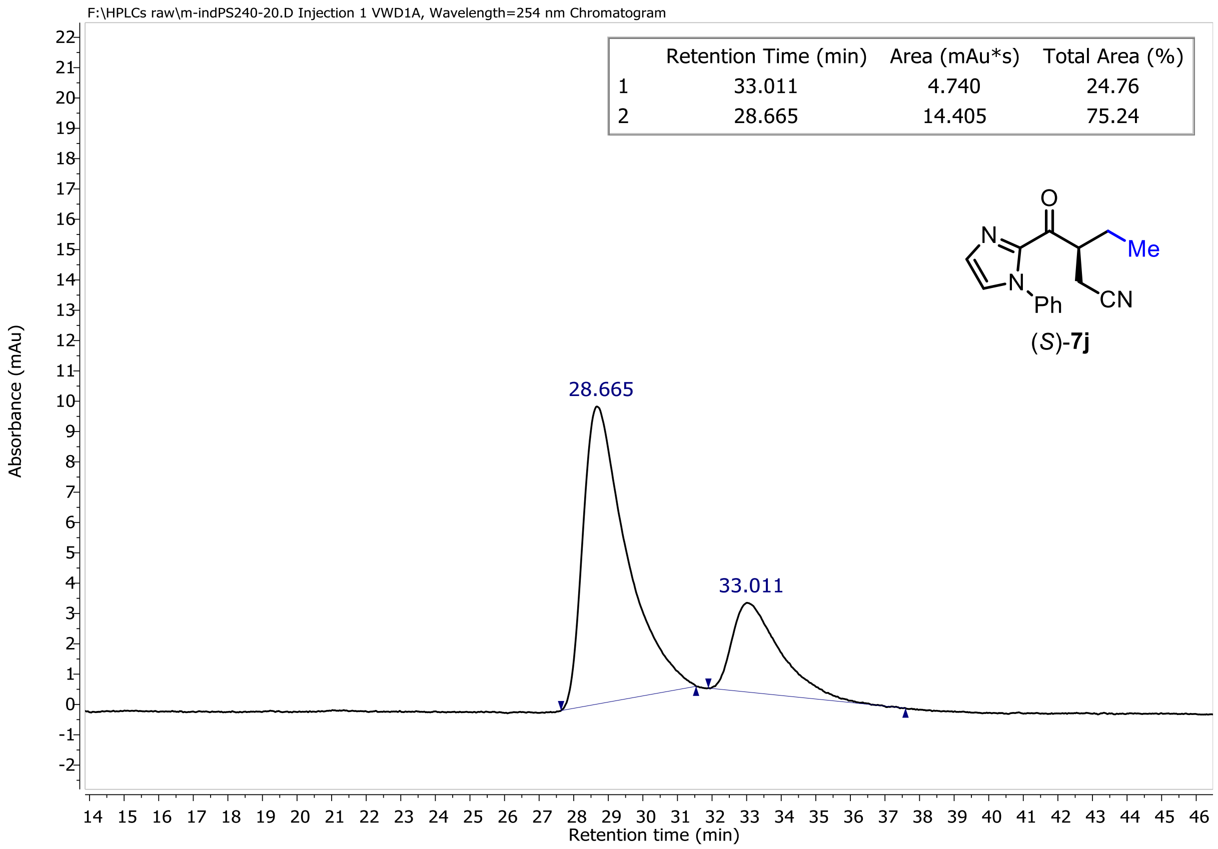

	Retention Time (min)	Area (mAu*s)	Total Area (%)
1	33.011	4.740	24.76
2	28.665	14.405	75.24

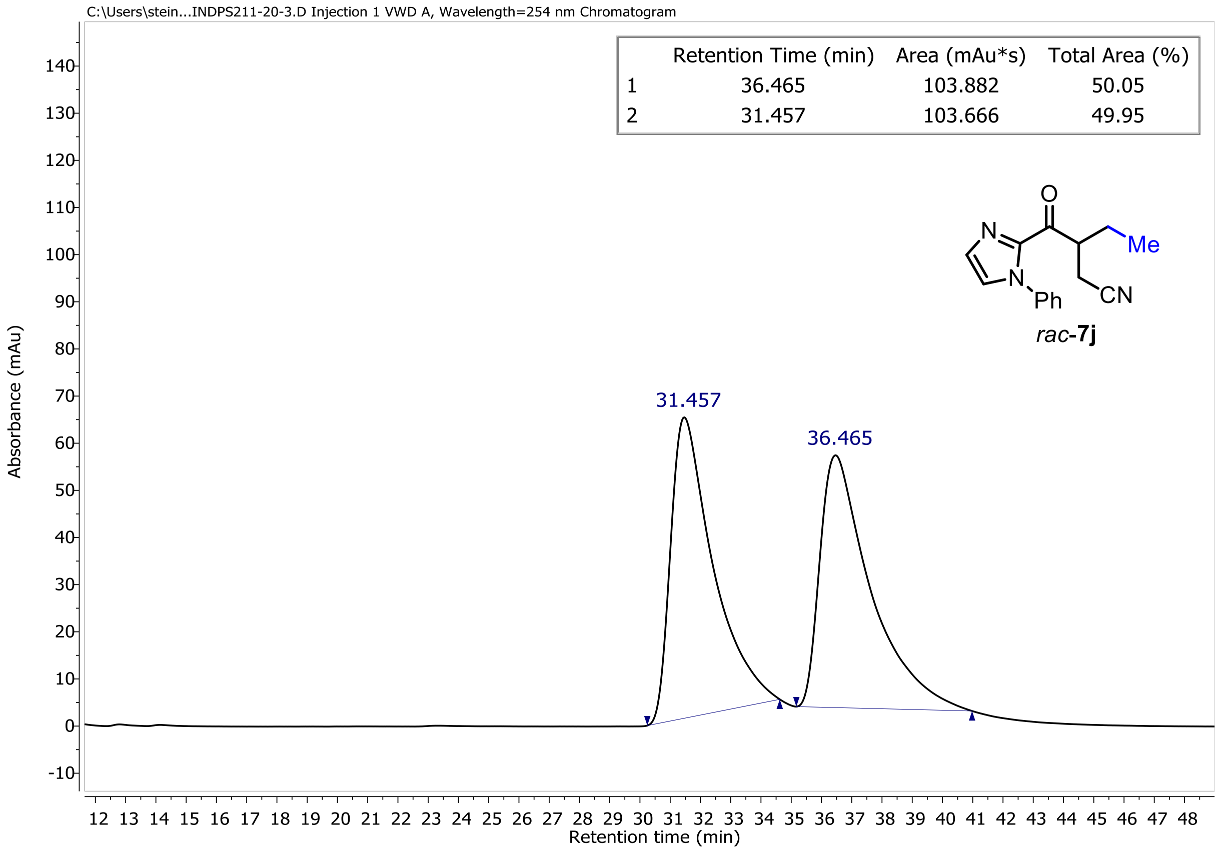

	Retention Time (min)	Area (mAu*s)	Total Area (%)
1	36.465	103.882	50.05
2	31.457	103.666	49.95

Figure S33. HPLC traces of (*S*)-**7j** (50% *ee*) and *rac*-**7j**.

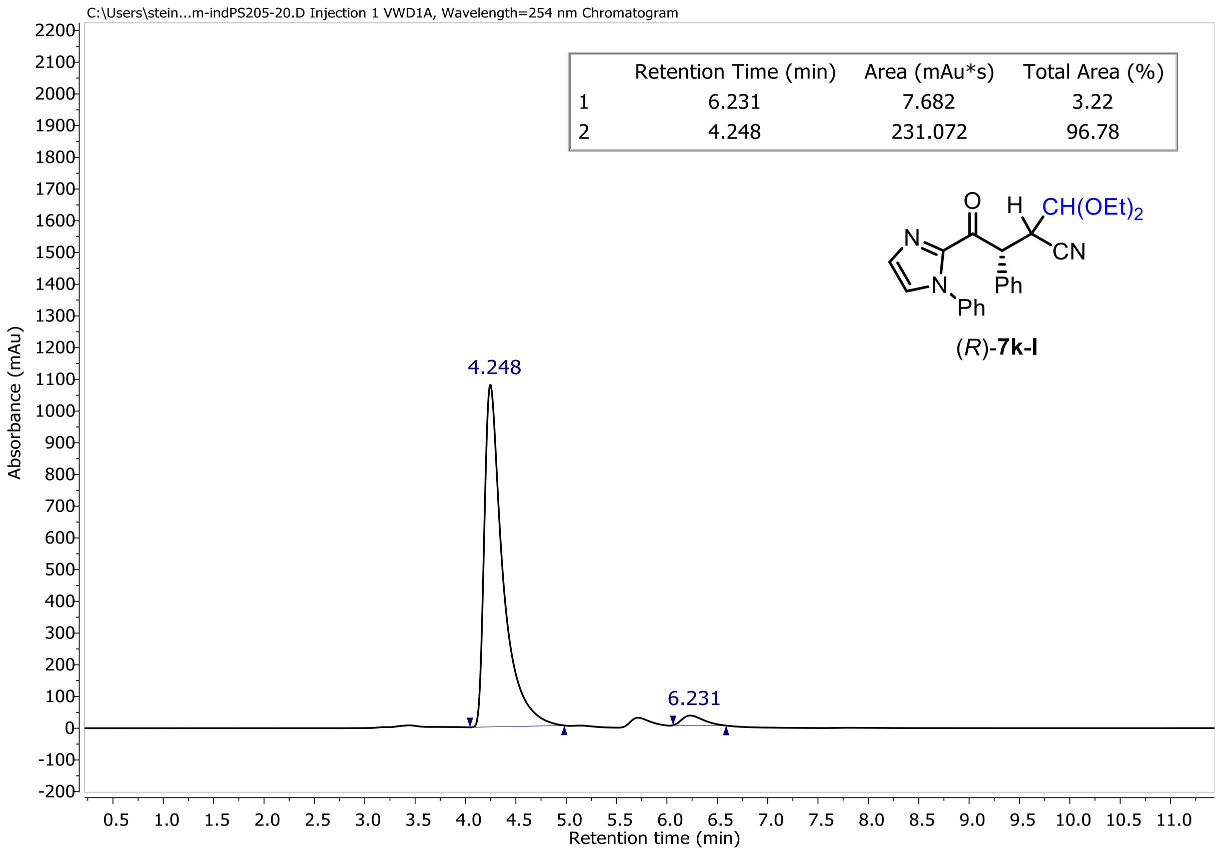

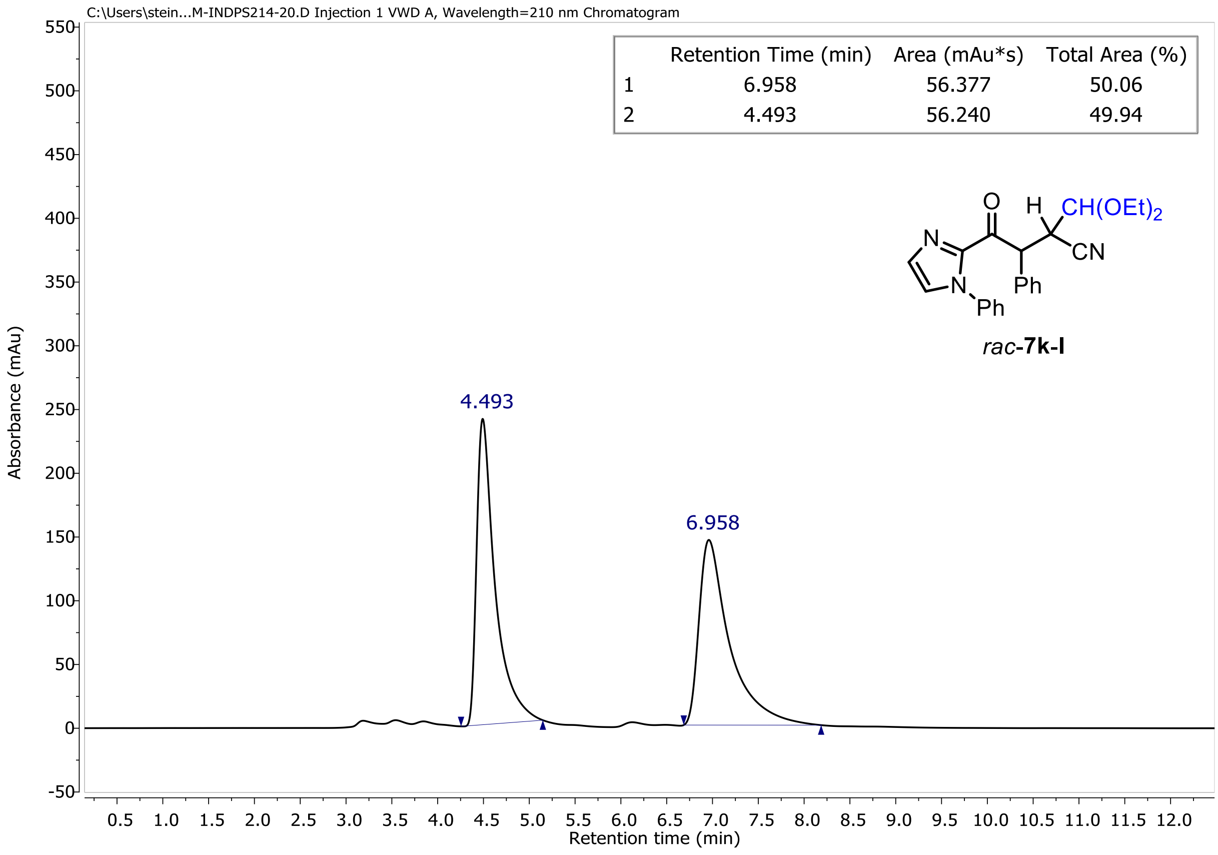

Figure S34. HPLC traces of (*R*)-**7k-I** (94% *ee*) and *rac*-**7k-I**.

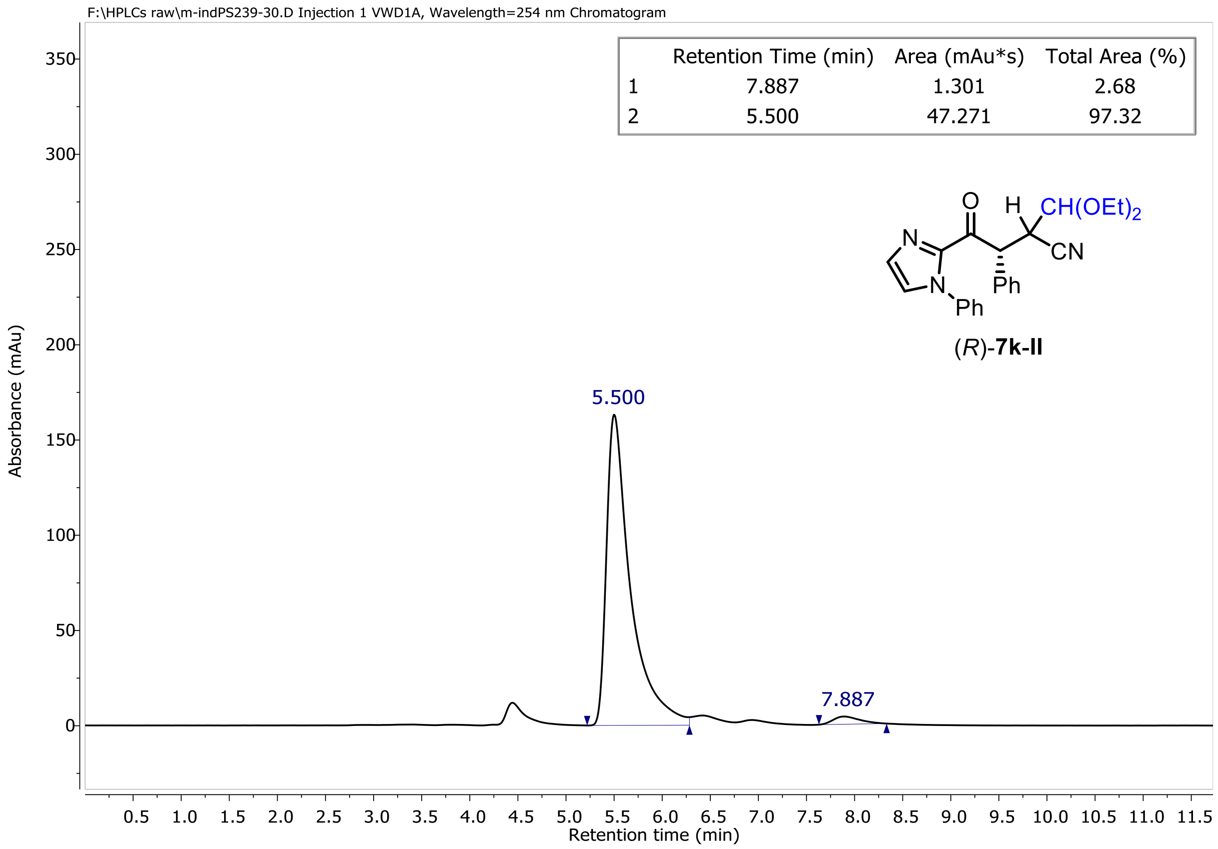

	Retention Time (min)	Area (mAu*s)	Total Area (%)
1	7.887	1.301	2.68
2	5.500	47.271	97.32

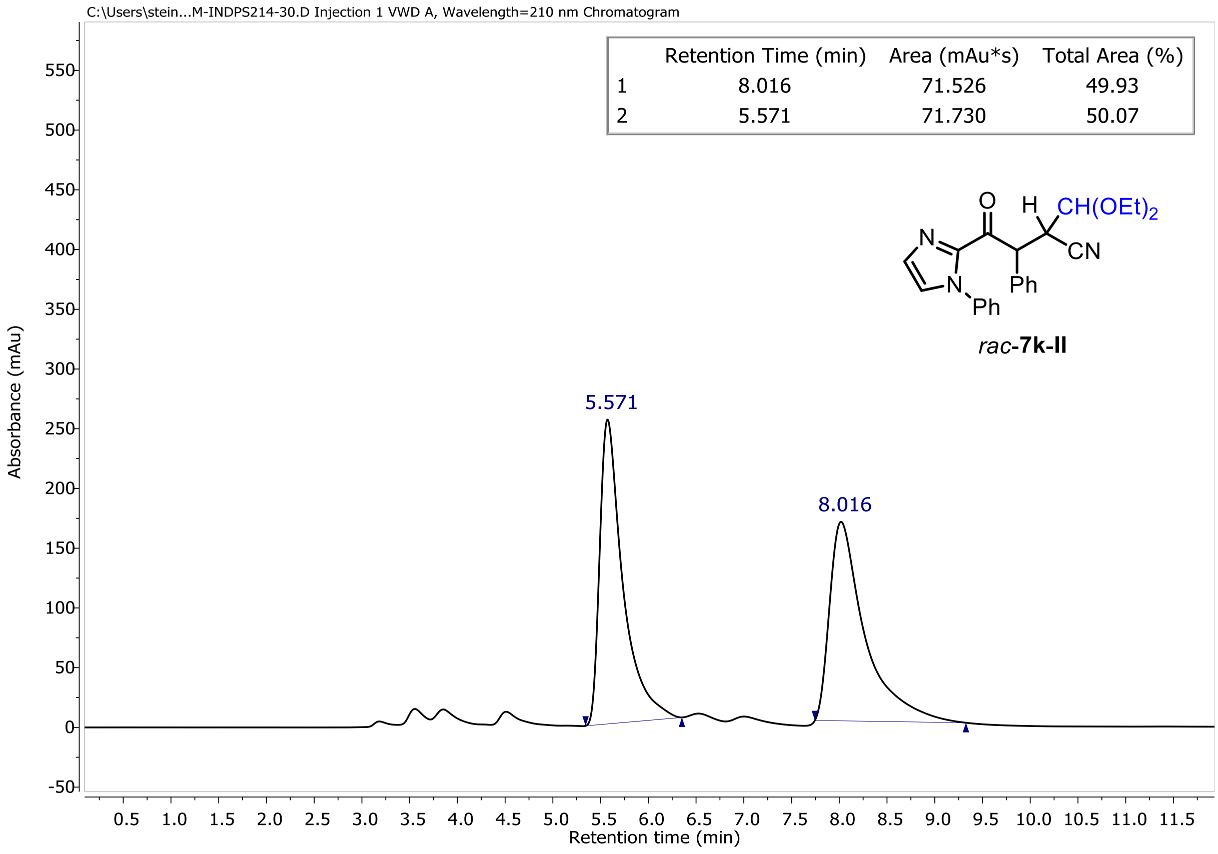

	Retention Time (min)	Area (mAu*s)	Total Area (%)
1	8.016	71.526	49.93
2	5.571	71.730	50.07

Figure S35. HPLC traces of (*R*)-**7k-II** (95% *ee*) and *rac*-**7k-II**.

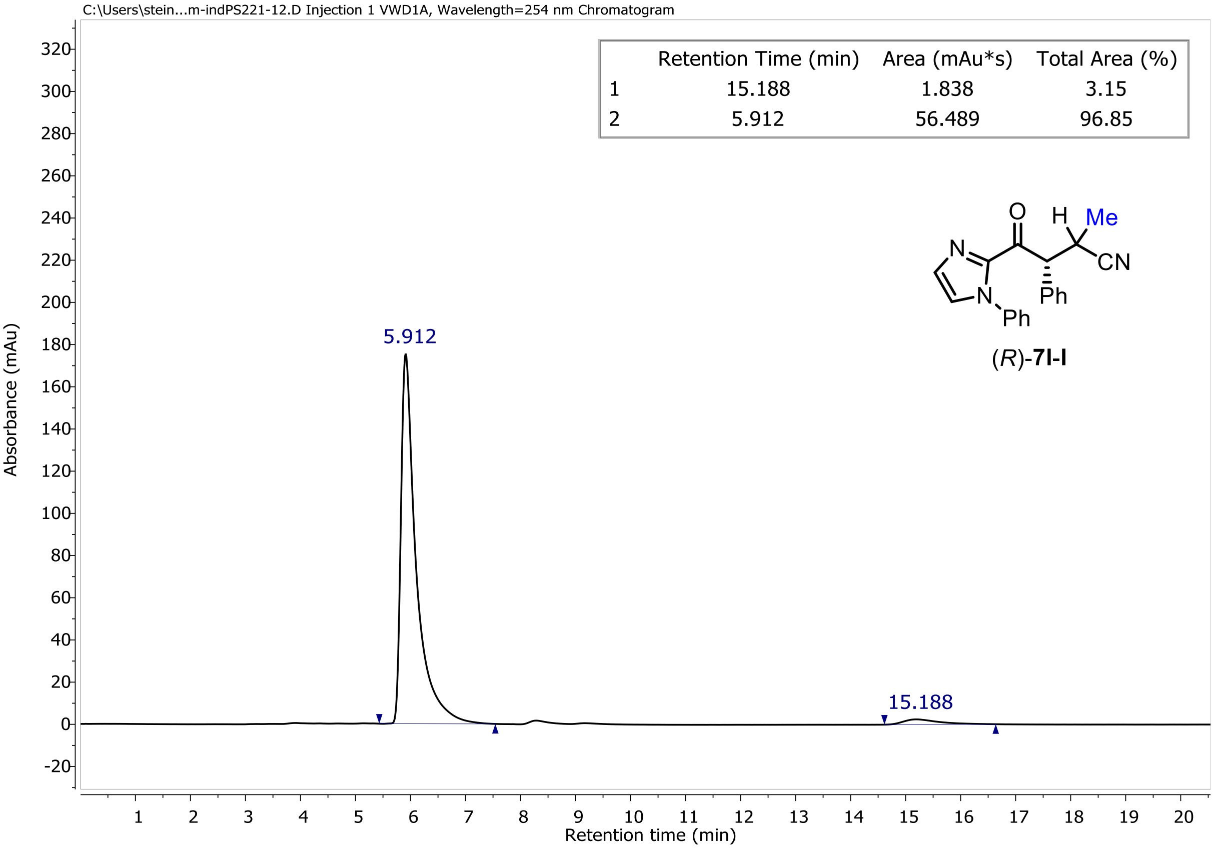

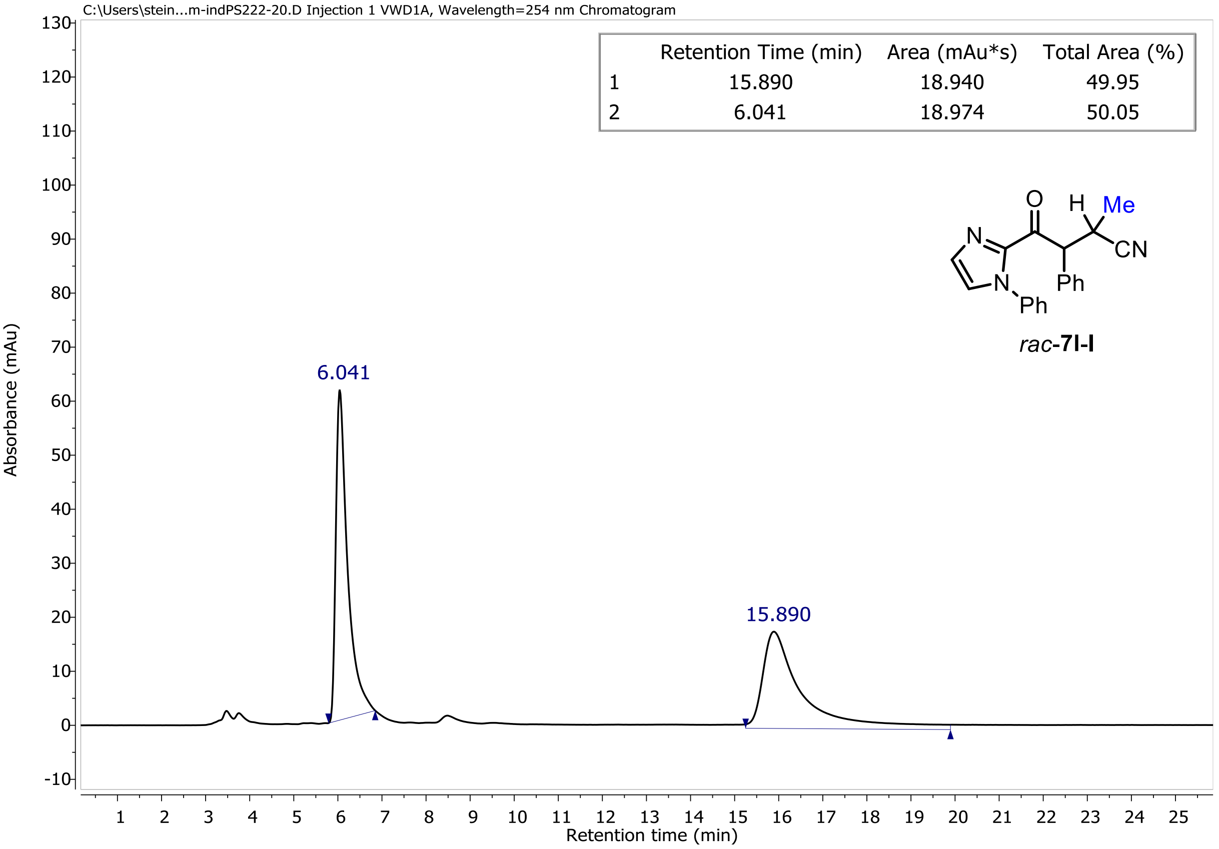

Figure S36. HPLC traces of (*R*)-**7l-I** (94% *ee*) and *rac*-**7l-I**.

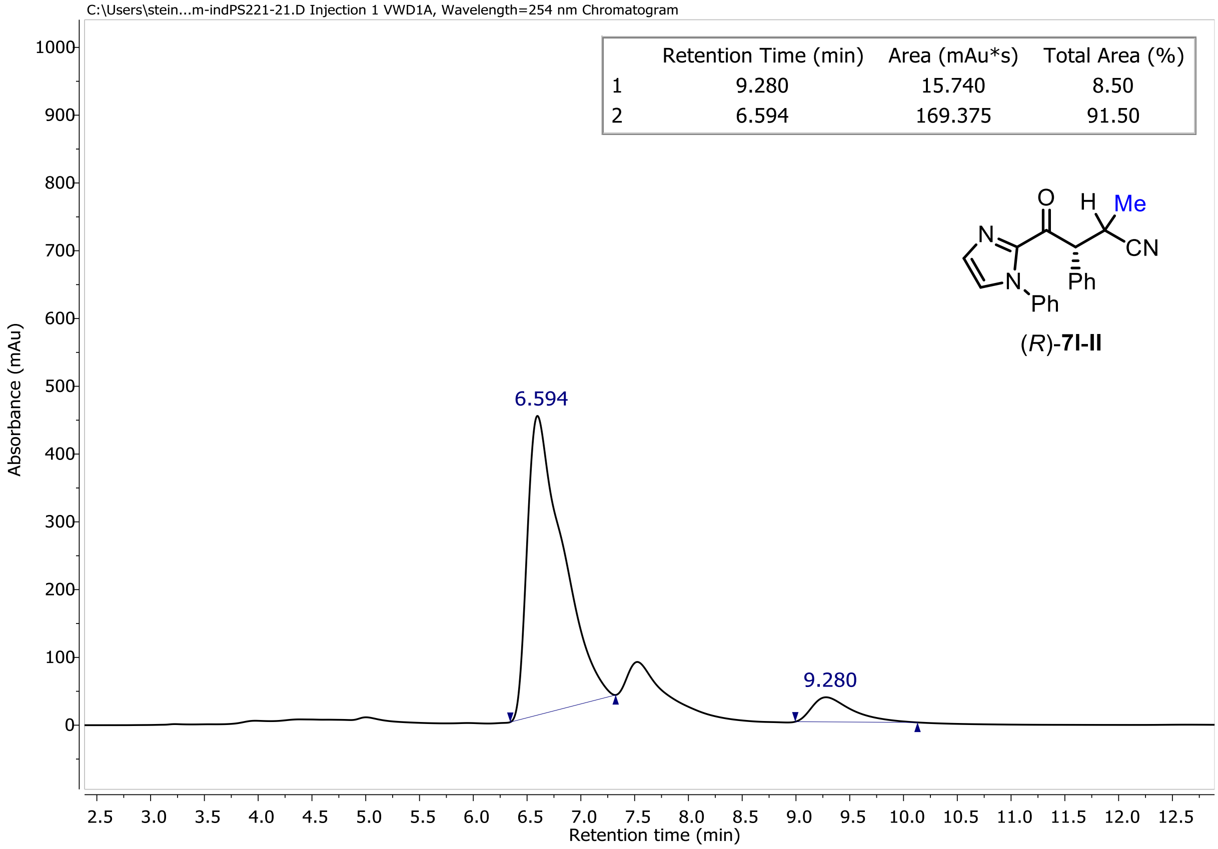

	Retention Time (min)	Area (mAu*s)	Total Area (%)
1	9.280	15.740	8.50
2	6.594	169.375	91.50

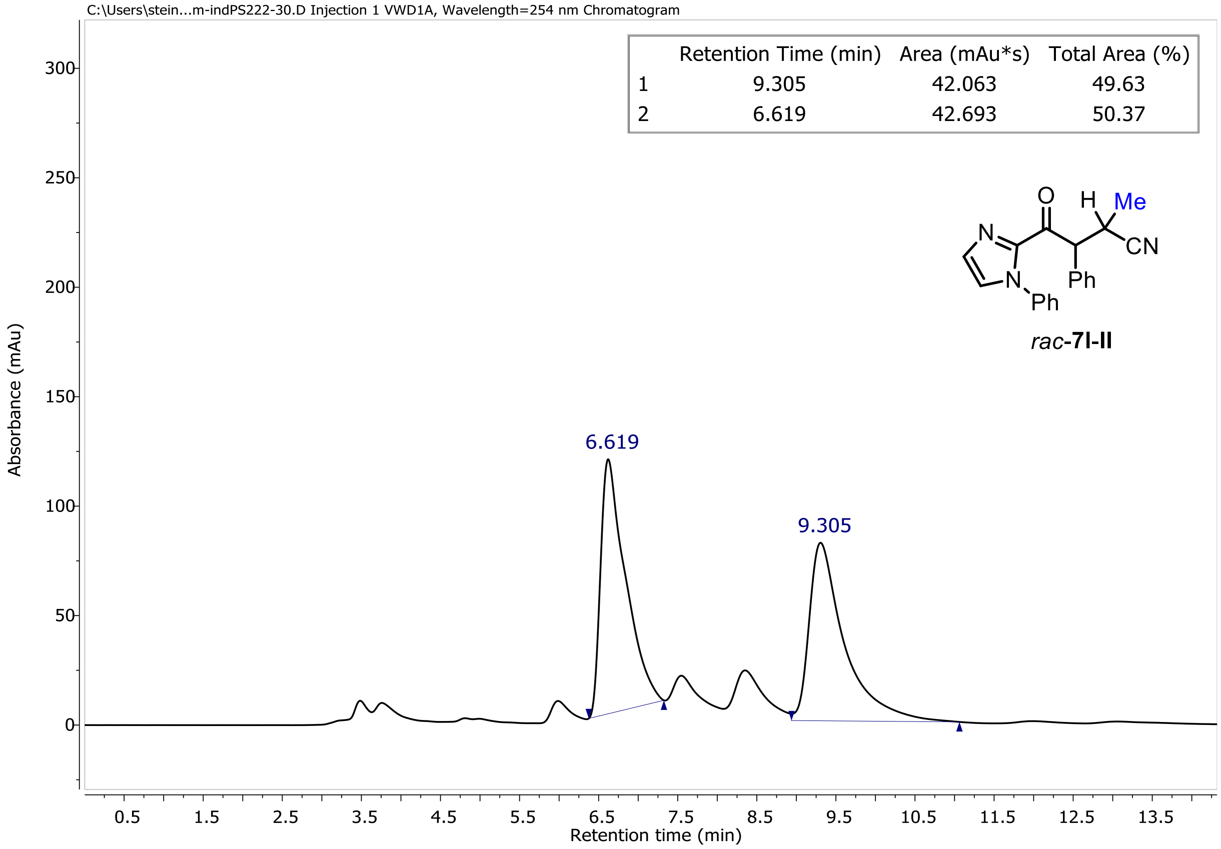

	Retention Time (min)	Area (mAu*s)	Total Area (%)
1	9.305	42.063	49.63
2	6.619	42.693	50.37

Figure S37. HPLC traces of (*R*)-**7l-II** (83% *ee*) and *rac*-**7l-II**.

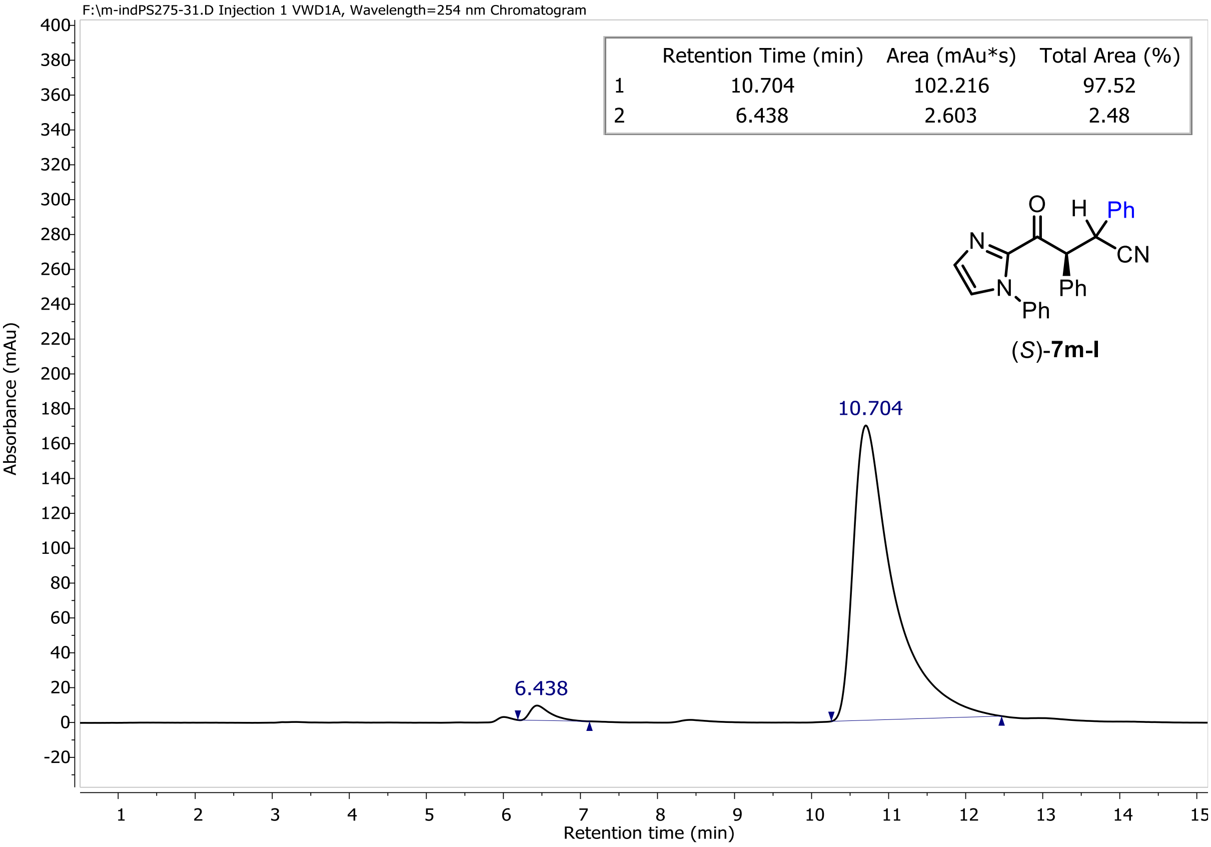

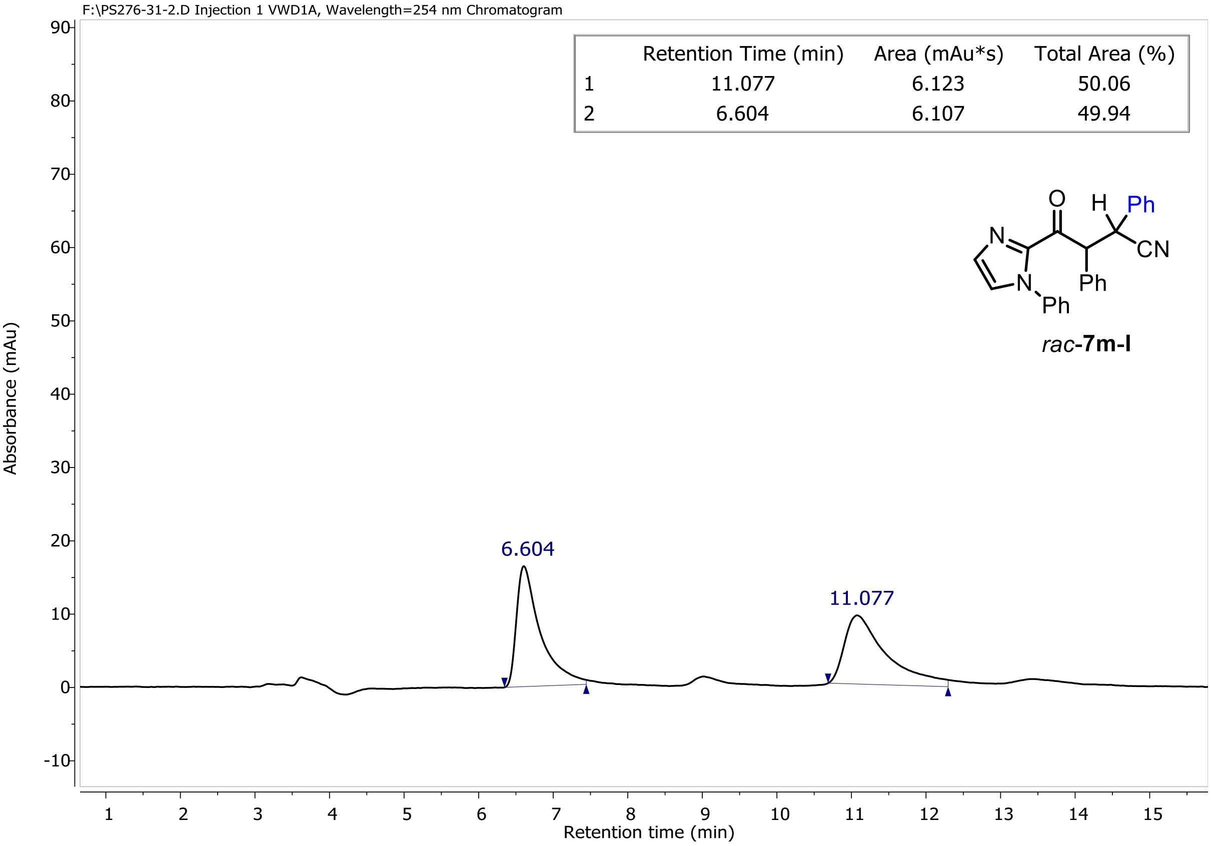

Figure S 38. HPLC traces of (*S*)-**7m-I** (95% *ee*) and *rac*-**7m-I**.

7 Single Crystal X-Ray Diffraction

X-ray data were collected[18] with a STOE STADIVARI diffractometer equipped with CuK_α radiation, a graded multilayer mirror monochromator ($\lambda = 1.54186$ Å) and a DECTRIS PILATUS 300 K detector using an oil-coated shock-cooled crystal at 100(2) K. Absorption effects were corrected semi-empirical using multiscanned reflexions (STOE LANA, absorption correction by scaling of reflection intensities[19,20]). The number of observed reflections of the data collection used for cell constant refinement is mentioned in Table S8 (cell determination).[21] The structure was solved by direct methods by using the program XT V2014/1 (Bruker AXS Inc., 2014)[22] and refined by full matrix least squares procedures on F^2 using SHELXL-2018/3 (Sheldrick, 2018).[23] The non-hydrogen atoms have been refined anisotropically, carbon bonded hydrogen atoms were included at calculated positions and refined using the 'riding model' with isotropic temperature factors at 1.2 times (for CH_3 groups 1.5 times) that of the preceding carbon atom. CH_3 groups were allowed to rotate about the bond to their next atom to fit the electron density. The absolute configuration of Λ-**RhInd** and compound **S7** has been determined.

Single crystals suitable for X-ray diffraction were obtained by preparation of a saturated solution of the complex (about 20 mg) in CH_2Cl_2 in a mass vial and slowly adding drops of Et_2O. After addition of three drops of Et_2O, the solution was left standing over night in the closed vessel. If no crystals were observed in the morning, the solution was placed in a fridge for ten hours. If again no crystals grew, another two drops of Et_2O were added and the solution again left standing over night at r.t.. This was repeated until single crystals suitable for X-ray diffraction could be obtained. Crystal structures, data and details of the structure determination are presented in Figure S39-40 and in Table S9.

Table S9. Crystal data and details for structure determintation.

	**Λ-RhInd**	(S)-**S7**
Identification code	**PS029**	**ZuoE-183e**
Habitus, colour	nugget, pale yellow	plate, colorless
Empiric formula	$C_{40}H_{44}Cl_4N_6RhF_6P$	$C_{20}H_{16}BrN_3O$
Cell determination	60111	9896
Formula weight	998.49	394.27
Crystal system, space group	Monoclinic<br>$C2$	$P2_12_12_1$
a, b, c (Å)	21.2750(2)<br>14.5957(2)<br>14.9938(1)	9.7170(4)<br>12.4043(5)<br>15.5021(7)
α, β, γ (°)	90<br>108.509(1)<br>90	90<br>90<br>90
V (Å^3)	4415.09(8)	1868.51(14)
Z	4	4
μ (mm^{-1})	6.232	2.212
Crystal size (mm)	0.40 x 0.25 x 0.14	0.61 x 0.14 x 0.03
No. of measured, independent and observed [$I > 2\sigma(I)$] reflections	43660<br>8761<br>8634	41914<br>4301<br>4085
R(int)	0.0246	0.0363
Goodness-of-fit on F^2	1.015	1.108
R index (all data)	wR2 = 0.0498	wR2 = 0.0753
R index conventional [$I > 2\sigma(I)$]	R1 = 0.0191	R1 = 0.0277
No. of reflections	8761	4301
No. of parameters	559	330
No. of restraints	1	798
T_{max}, T_{min}	0.0416, 0.0084	0.94, 0.65

$\Delta\rho_{max}$, $\Delta\rho_{min}$ (e Å^{-3})	0.328 and –0.384	0.854 and –0.429
Temperature (K)	100(2)	115(2)
Wavelength (Å)	1.54186	0.71073
Flack parameter[24] (absolute structure)	–0.015	–0.010
CCDC no.	1939572	1939571

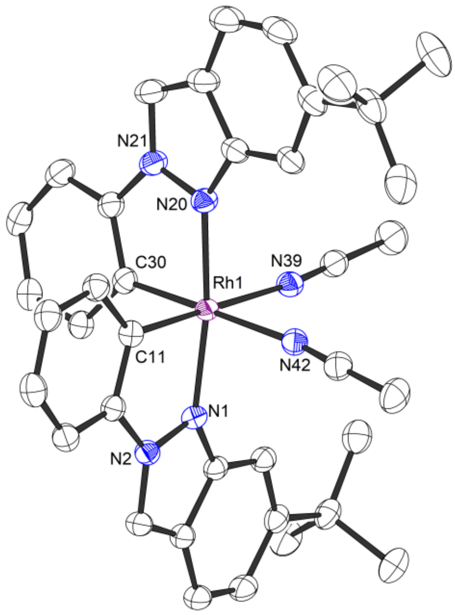

Figure S39. Crystal structure of Λ-**RhInd**. Solvent molecules and the hexafluorophosphate counterion are omitted for clarity. ORTEP drawing with 50% probability of thermal ellipsoids.

Absolute configurations of the products for the α-cyanoalkylation of 2-acyl imidazoles are based on the comparison with the crystal structure of enantiopure compound (*S*)-**S7**. The crystal structure is shown in Figure S40. Compound (*S*)-**S7** was obtained using Δ-**RhS** as catalyst.

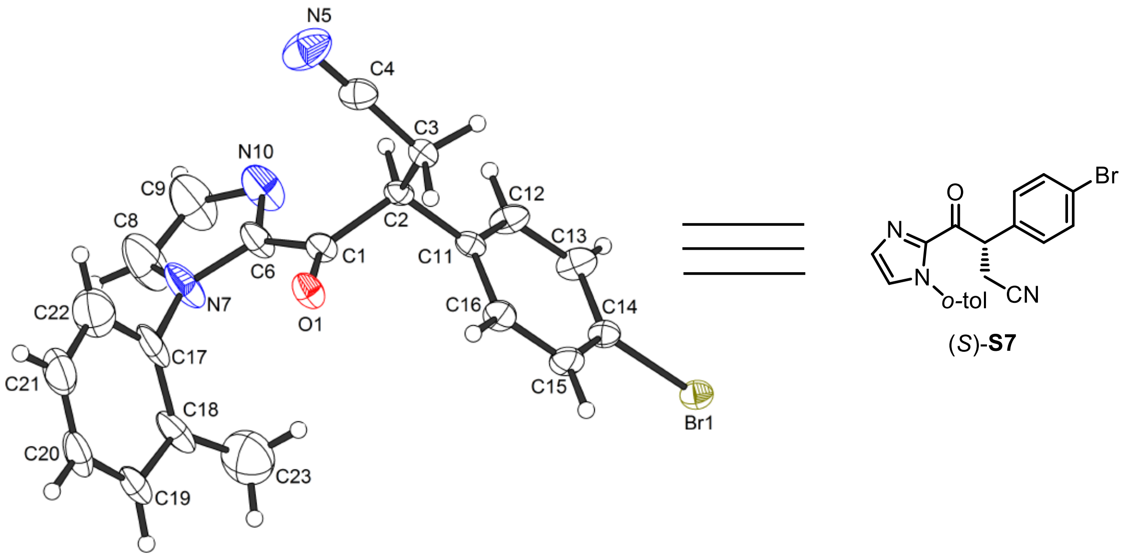

Figure S40. Crystal structure of (*S*)-**S7**. Solvent molecules are omitted for clarity. ORTEP drawing with 50% probability of thermal ellipsoids.

8 References

[1] X. Huang, X. Li, X. Xie, R. Riedel, E. Meggers, *Nat. Commun.* **2017**, *8*, 2245.

[2] E. M. Doherty, C. Fotsch, Y. Bo, P. P. Chakrabati, N. Chen, N. Gavva, N. Han, M. G. Kelly, J. Kincaid, L. Klionsky, Q. Liu, V. I. Ognyanov, R. Tamir X. Wang, J. Zhu, M. H. Norman, J. J. S. Treanor, *J. Med. Chem.* **2005**, *48*, 71-90.

[3] G. Bogonda, H. Y. Kim, K. Oh, *Org. Lett.* **2018**, *20*, 2711-2715.

[4] J. Ma, X. Shen, K. Harms, E. Meggers, *Dalton Trans.* **2016**, *45*, 8320-8323.

[5] J. Ma, X. Zhang, X. Huang, S. Luo, E. Meggers, *Nat. Protocols* **2018**, *13*, 605-632.

[6] X. Shen, K. Harms, M. Marsch, E. Meggers, *Chem. Eur. J.* **2016**, *22*, 9102-9105.

[7] C. Wang, Y. Zheng, H. Huo, P. Röse, L. Zhang, K. Harms, G. Hilt, E. Meggers, *Chem. Eur. J.* **2015**, *21*, 7335-7359.

[8] Y. Tan, W. Yuan, L. Gong, E. Meggers, *Angew. Chem. Int. Ed.* **2015**, *54*, 13045-13048.

[9] E. R. Welin, A. A. Warkentin, J. C. Conrad, D. W. C. MacMillan, *Angew. Chem. Int. Ed.* **2015**, *54*, 9668-9672.

[10] P. Dowd, C. Kaufman, P. Kaufman, *J. Org. Chem.* **1985**, *50*, 882-885.

[11] P. Molina, C. López-Leonardo, J. J. Llamas-Botía, C. Foces-Foces, C. Fernández-Castaño, *Tetrahedron,* **1996**, *52*, 9629-9642.

[12] E. D. Matveeva, T. A. Podrugina, E. V. Tishkovskaya, N. S. Zefirov, *Mendeleev Commun.* **2003**, *13*, 260-261.

[13] N. D. C. Tappin, M. Gnägni-Lux, P. Renaud, *Chem. Eur. J.* **2018**, *24*, 11498-11502.

[14] K. Hattori, K. Yamaguchi, J. Yamaguchi, K. Itami, *Tetrahedron* **2012**, *68*, 7605-7612.

[15] Y. Tan, W. Yuan, L. Gong, E. Meggers, *Angew. Chem. Int. Ed.* **2015**, *54*, 13045-13048.

[16] X. Huang, R. D. Webster, K. Harms, E. Meggers, *J. Am. Chem. Soc.* **2016**, *138*, 12636-12642.

[17] X. Huang, J. Lin, T. Shen, K. Harms, M. Marchini, P. Ceroni, E. Meggers, *Angew. Chem. Int. Ed.* **2018**, *57*, 5454-5458.

[18] *X-Area Pilatus3_SV*, STOE & Cie GmbH, Darmstadt, Germany, **2016**.

[19] *X-Area LANA*, STOE & Cie GmbH, Darmstadt, Germany, **2016**.

[20] *X-Area Integrate*, STOE & Cie GmbH, Darmstadt, Germany, **2016**.

[21] *X-Area Recipe*, STOE & Cie GmbH, Darmstadt, Germany, **2015**.

[22] G. M. Sheldrick, *Acta Cryst. A* **2015**, *71*, 3-8.

[23] G. M. Sheldrick, *Acta Cryst. C* **2015**, *71*, 3-8.

[24] S. Parsons, H. D. Flack, T. Wagner, *Acta Cryst. B* **2013**, *69*, 249-259.
